# An unnatural base pair for the detection of epigenetic cytosine modifications in DNA

**DOI:** 10.1038/s41557-025-01925-6

**Published:** 2025-08-20

**Authors:** David Schmidl, Sidney M. Becker, James M. Edgerton, Shankar Balasubramanian

**Affiliations:** 1 Yusuf Hamied Department of Chemistry, University of Cambridge, Cambridge, UK; 2 Cancer Research UK Cambridge Institute, Li Ka Shing Centre, University of Cambridge, Cambridge, UK; 3 School of Clinical Medicine, University of Cambridge, Cambridge, UK

**Keywords:** DNA, DNA sequencing, Nucleic acids, Methylation analysis, DNA

## Abstract

Natural, covalently modified cytosine bases within genomic DNA function as important epigenetic markers. Approaches for single-base-resolution sequencing of cytosine modifications typically deploy chemistry for modification-selective C-to-T code conversion and can require error-prone subtractive analysis of complex data. Here we report the sequencing of an epigenetic base by exploiting an unnatural base pair system. This approach relies on hydrogen-bonding complementarity between a malononitrile adduct of 5-formylcytosine and protonated 3,7-dideazaadenine. The specificity of this unnatural base pair was studied by biophysical DNA thermal melting analysis and by template-directed incorporation by DNA polymerase enzymes. Base pair selectivity was enhanced by controlling the protonation state of 3,7-dideazaadenine. We exemplify use of this unnatural base pair to sequence 5-formylcytosine in a DNA template using a Sanger-type format. There is scope for this base pair and the general concept to be implemented on further sequencing platforms that exploit Watson–Crick base pairing to directly identify epigenetic bases.

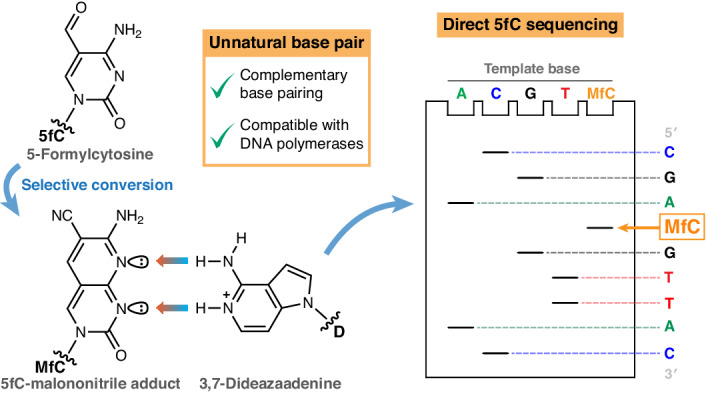

## Abstract (web summary)

Cytosine modifications within genomic DNA can serve as important epigenetic markers, but sequencing them at single-base resolution requires chemical modification-selective C-to-T code conversion with potential complex analysis. Now an unnatural base pair enables the sequencing of an epigenetic modification—5-formylcytosine—at single-base resolution within a Sanger sequencing format.

## Main

Naturally occurring cytosine modifications, 5-methylcytosine (5mC) and its oxidized derivatives 5-hydroxymethyl-, 5-formyl- and 5-carboxylcytosine (5hmC, 5fC and 5caC, respectively), constitute part of the epigenetic code in genomic DNA. They can be found at varying levels in different cell and tissue types^1,2^. 5mC and 5hmC are important epigenetic markers involved in the regulation of gene expression, particularly during cell differentiation and embryonal development^3–6^. They are also of clinical interest as disease biomarkers, and enzymes involved in their metabolism are considered drug targets^7,8^. 5fC and 5caC are known as key intermediates in the active demethylation of genomic DNA^9,10^, and a potential epigenetic role for 5fC has recently been described^11^. However, partly due to their low abundance in the genome, functionality as epigenetic markers has yet to be conclusively established, despite evidence for the existence of further selective protein readers^12,13^.

**Fig. 1:**
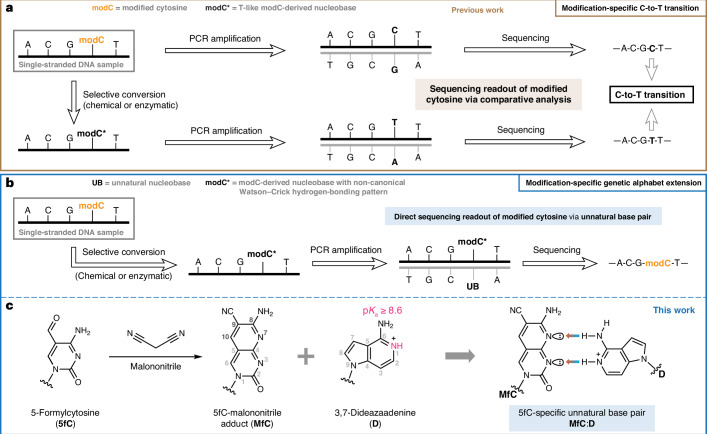
Concepts for the detection of cytosine modifications (modCs) among canonical nucleobases in epigenetic sequencing. **a**, Subtractive modification readout by C-to-T transition, as relied upon by common polymerase-based methods. modC, which is paired with G by a DNA polymerase, is chemically converted into an analogue (modC*) that is instead paired with A. Comparison of the sequencing readout after conversion with a native reference sequence identifies the modC position. **b**, Genetic alphabet extension as an alternative concept for non-subtractive modC readout, as explored in this work. modC is chemically converted into a modC* with a non-canonical Watson–Crick hydrogen-bonding pattern that is paired with an unnatural base (UB) by a DNA polymerase. Orthogonal amplification and sequencing of this unnatural base pair enables direct modC identification within the native genetic sequence. **c**, The 5fC-specific unnatural base pair investigated in this work, consisting of **MfC** and protonated **D** (predominantly existing below pH 8.6), for the purpose of direct 5fC detection by Sanger sequencing (in grey: atomic numbering based on conventional pyrimidine and purine numbering; Watson–Crick hydrogen bonds from donor (blue) to acceptor (red) are indicated by coloured arrows).

Several methods for sequencing cytosine modifications at base resolution have been developed. Most of them are compatible with next-generation sequencing (NGS) platforms such as Solexa-Illumina sequencing. Third-generation sequencing platforms have the potential to detect modified bases directly, but they suffer from higher error rates and require extensive machine learning^14^. Therefore, a common strategy for detecting modified cytosines relies on selective chemical transformation of one or more of the C derivatives into a nucleobase with T-like hydrogen-bonding pattern and a comparative readout by NGS (Fig. 1). Examples include bisulfite-^15^ or deaminase-induced^16^ hydrolytic deamination of cytosine to uracil, whereby the modified cytosine of interest is ‘protected’ and preserved as a ‘C’ in the sequencing readout. Conversely, the cytosine modification itself is specifically transformed, for instance, through (photo)reductive deamination to yield dihydrouracil^17,18^, or a Friedlaender-type chemical transformation generating an adduct that pairs with adenine^19,20^ (Fig. 1a). In all cases, the modification is identified by comparison of the sequencing readout after (bio)chemical conversion with a reference, and by observation of a C-to-T transition.

There are key limitations to such approaches. First, conversion of one canonical hydrogen-bonding pattern (for example, C) into another (for example, T) will cause some information loss. For example, the common naturally occurring C-to-T mutation^21^ will not be easily discernible from C-to-T transitions at epigenetic sites. Second, if unmodified cytosines are converted to ‘T’ (for example, upon bisulfite treatment), the sequenced genome is largely reduced in complexity to three bases, making comparative computational analysis difficult and error-prone. A recently described approach couples nucleobase conversion to a two-base coding system via a copy strand to sequence genetic and epigenetic bases next to each other^22^.

Expanding the base-pairing alphabet could enable direct readout of genetic bases and modified cytosines in parallel, avoiding the need for comparative analysis or loss of information (Fig. 1b). An unnatural base pair with a hydrogen-bonding pattern that is differentiated from the standard Watson–Crick pairs could facilitate such a strategy. Unnatural bases paired via complementary hydrogen-bonding patterns^23,24^ or shape complementarity^25–27^ have been developed for biotechnological and medicinal applications^28^. For instance, they have been employed for polymerase chain reaction (PCR)-based nucleic acid detection^29,30^ and in vitro evolution of DNA aptamers^31,32^, and have been implemented into in vitro transcription and translation processes as well as a semi-synthetic organism^24,33–36^. DNA and RNA polymerases process unnatural base pairs if the arrangement of the 2′-deoxynucleoside 5′-triphosphate (dNTP) and the templating nucleotide conforms with tight spatial constraints within the active site (‘Watson–Crick geometry’) that determine dNTP incorporation kinetics and selectivity^37,38^.

Polymerase-based sequencing using unnatural base pairs has previously been attempted for DNA lesions, such as abasic sites and 3-methyl-T, via Sanger sequencing^39,40^. However, although the utilized hydrophobic base pair is compatible with PCR amplification, this strategy only enabled the detection of a single lesion site due to complete chain termination at that position in the sequencing reaction. Similarly, 8-oxo-G could be identified at a targeted site using a size-expanded nucleobase in single-nucleotide primer elongation assays^41^. Consecutive, polymerase-dependent sequencing of naturally occurring modified nucleobases in DNA via an unnatural base pair has not yet been achieved, and to the best of our knowledge no such attempt has been described to read epigenetic cytosine modifications.

In this Article we report the development of an unnatural base pair (Fig. 1c) between a malononitrile adduct of 5fC and 3,7-dideazaadenine and demonstrate that this orthogonal base pairing can be used to detect 5fC consecutively at base resolution via a Sanger sequencing approach.

## Design rationale for the MfC:D base pair

Detection of an epigenetic modification by genetic code extension requires a base-pairing pattern that is differentiated from T:A and C:G Watson–Crick hydrogen bonding. The biocompatible Friedlaender-like conversion of 5fC with malononitrile^20^ yields a unique unnatural base (**MfC**) with three hydrogen-bond acceptor positions across the Watson–Crick edge (Fig. 1c). As routes exist to convert 5mC and 5hmC into 5fC^42–45^, we considered 5fC a useful exemplar for the readout of epigenetic information via an unnatural base pair, with **MfC** as the first element.

A suitable pairing partner should sterically complement **MfC** in the base-pairing geometry, forming two or three productive Watson–Crick hydrogen bonds. Thus, a purine base with two hydrogen-bond donors would be beneficial. Adenine pairs with **MfC** in the correct geometry^46^, but possesses a hydrogen-bond acceptor in N1. We hypothesized that conversion of N1 in adenine into a charged hydrogen-bond donor by protonation might provide a fully complementary base-pairing pattern to **MfC**. The structural adenine analogue 3,7-dideazaadenine (**D**) is more electron-rich, resulting in higher basicity (p*K*_a_ = 8.6 in 2′-deoxynucleoside **dD**^47^) than for adenine (p*K*_a_ = 3.8 in 2′-deoxyadenosine^48^). Considering that **D** should have an even higher p*K*_a_ in a negatively charged oligonucleotide or dNTP and most DNA polymerases operate most efficiently at neutral to alkaline pH, **D** could be protonated under polymerase-compatible conditions. We reasoned that protonated **D** would form a cognate base pair with **MfC** (Fig. 1c) that, enabled by hydrogen-bonding complementarity, could be recognized by DNA polymerases in the Watson–Crick geometry, extending the canonical four-letter genetic alphabet for the detection of a modified cytosine.

## Protonated D forms a stable base pair with MfC in DNA duplexes

**Fig. 2:**
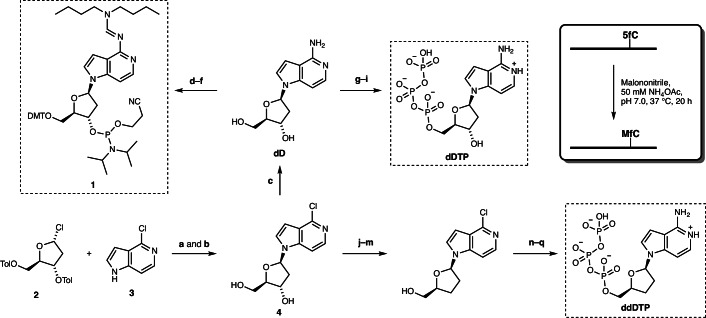
Chemical synthesis of D-containing phosphoramidite 1,2′-deoxynucleoside triphosphate dDTP and 2′,3′-dideoxynucleoside triphosphate ddDTP, as well as MfC-containing DNA oligonucleotides. **a**–**q**, Conditions: KOH, THF, room temperature (r.t.), 1 h, then THF, r.t., 15 min, 78% (**a**); NH_3_, MeOH, 55 °C, 3 days, 96% (**b**); N_2_H_4_**·**H_2_O, 125 °C, 4 h, then Raney-Ni, H_2_O, 100 °C, 1 h, 80% (**c**); ^*n*^Bu_2_NCH(OMe)_2_, DMF, 80 °C, 5 h (**d**); DMTCl, DMAP, pyridine, r.t., 24 h, 49% over 2 steps (**e**); ^*i*^Pr_2_NPCl(OC_2_H_4_CN), 1-methylimidazole, DIPEA, 0 °C to r.t., 5 h, 60% (**f**); POCl_3_, (EtO)_3_PO, 0 °C, 4 h, 52% (**g**); (pyrS)_2_, PPh_3_, morpholine, DMSO, r.t., 2 h (**h**); [(^*n*^Bu_3_NH)_2_](H_2_P_2_O_7_), 4,5-dicyanoimidazole, DMF, r.t., 6 h, 29% over 2 steps (**i**); DMTCl, DMAP, pyridine, 0 °C to r.t., 48 h, 77% (**j**); PhOC(S)Cl, DMAP, MeCN, r.t., 20 h, 72% (**k**); ^*n*^Bu_3_SnH, AIBN, toluene, 80 °C, 3 h (**l**); dichloroacetic acid, DCM, r.t., 20 min, 65% over 2 steps (**m**); N_2_H_4_**·**H_2_O, 125 °C, 5 h, then Raney-Ni, H_2_O, 100 °C, 6 h, 82% (**n**); POCl_3_, (EtO)_3_PO, 0 °C, 3 h, 48% (**o**); (pyrS)_2_, PPh_3_, morpholine, DMSO, r.t., 2.5 h (**p**); [(^*n*^Bu_3_NH)_2_](H_2_P_2_O_7_), 4,5-dicyanoimidazole, DMF, r.t., 2.5 h, 24% over 2 steps (**q**). Inset: conversion of 5fC into **MfC** on single-stranded DNA^20^.

To evaluate the specificity of the **MfC**:**D** base pairing, we carried out biophysical thermal ultraviolet (UV) melting studies on double-stranded DNA comprising **MfC** and **D**. To prepare **D**-containing oligonucleotides, we synthesized phosphoramidite **1** starting from Hoffer’s chlorosugar (**2**) and nucleobase precursor **3** (ref. ^47^; Fig. 2). Oligonucleotides comprising **MfC** were generated by chemically transforming 5fC-containing oligonucleotides through reaction with malononitrile^20^.

**Fig. 3:**
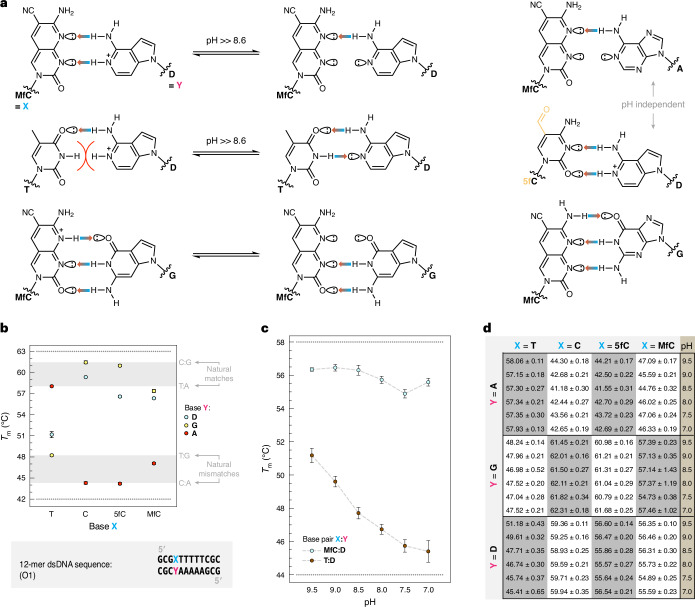
Base pairing properties of the unnatural bases MfC and D. Assessment of thermodynamic base-pair stabilities by DNA duplex melting experiments. **a**, Putative geometries of the selected base pairs. Interaction of **MfC**:**D** and T:**D** base pairs (as well as of the **MfC**:G base pair) can depend on pH. Favourable protonation of **D** (p*K*_a_ ≥ 8.6) strengthens **MfC**:**D** and destabilizes T:**D** due to steric clash of the endocyclic NH moieties. Structures of **MfC**:A and (5f)C:**D** are not easily influenced by pH. **b**, Melting temperatures (*T*_m_) of DNA 12-mer duplexes (sequence O1) possessing varying pyrimidine:purine (X:Y) pairings measured at pH 9.5, with indication of temperature ranges for canonical matched and mismatched base pairs. **c**, pH dependence of melting temperatures for **MfC**:**D** and T:**D** pairings between pH 9.5 and pH 7.0. **d**, Tabulated melting temperatures for all X:Y pairings shown in **b** (in °C), dependent on pH. Means of three independent experiments are shown with s.d. (*n* = 3). Source data

We assessed the relative stability of a series of pyrimidine:purine base pairs by UV thermal melting analysis of DNA double-stranded 12-mers (Supplementary Table 1). As the hydrogen-bonding pattern of **D** could complement either **MfC** or T, depending on its protonation state (Fig. 3a), melting temperatures (*T*_m_) were determined at various pH values around the p*K*_a_ of **dD** (pH 9.5–7.0). Interestingly, the melting temperature of a first duplex (O1) containing **MfC**:**D** at pH 9.5 (56.3 ± 0.1 °C; Fig. 3b) was markedly higher than that of an analogous **MfC**:A duplex (47.1 ± 0.2 °C) that possesses one hydrogen bond (Fig. 3a). Simultaneously, the *T*_m_ of a double strand with a cognate T:A (58.1 ± 0.1 °C) pair, which contains two hydrogen bonds (Fig. 3a), was only slightly higher than that of the **MfC**:**D** duplex (Fig. 3b). Given that DNA hybridization can favour nucleobase protonation if that leads to hydrogen bonding^49^, and probably also due to favourable secondary hydrogen bonding^50^, this suggests the formation of two productive hydrogen bonds between **MfC** and **D** already at this pH (Fig. 3a). In contrast, the T:**D** duplex (*T*_m_ = 51.2 ± 0.4 °C) was substantially less stable than the cognate T:A (*T*_m_ = 58.1 ± 0.1 °C) and also the **MfC**:**D** duplex (*T*_m_ = 56.3 ± 0.1 °C) at pH 9.5, despite the expected A-like appearance of unprotonated **D**. Notably, the **MfC**:G duplex melted at a similar temperature (57.4 ± 0.2 °C) as the **MfC**:**D** double strand. The **MfC**:G base pair has been reported to adopt the wobble geometry within double-stranded DNA, which provides enhanced stability through three hydrogen-bond contacts^46^ (Fig. 3a). Alternatively, protonation of the **MfC** base could provide a three-contact Watson–Crick base pair.

Lowering the pH progressively destabilized the T:**D** pair due to steric clash introduced upon protonation of **D** (Fig. 3a), resulting in *T*_m_ reduction from 51.2 ± 0.4 °C at pH 9.5 to 45.4 ± 0.6 °C at pH 7.0 (Fig. 3c). Conversely, the stability of **MfC**:**D** duplexes remained relatively constant (*T*_m_ = 56.5 ± 0.2 °C to 54.9 ± 0.3 °C) across the same pH range. The minor *T*_m_ reduction (~1 °C) towards pH 7.0 could be caused by more favourable hydration of the protonated **dD** nucleotide, which stabilizes the single strand after dissociation, slightly shifting the hybridization equilibrium^51^. No other base-pair combination showed substantial shifts in melting temperature with changing pH (Fig. 3d). However, stable C:**D** (*T*_m_ = 59.4 ± 0.1 °C at pH 9.5) and 5fC:**D** (*T*_m_ = 56.6 ± 0.1 °C at pH 9.5) pairs were formed, probably in wobble geometry, analogous to a protonated C:A pair^49^.

**Extended Data Fig. 1:**
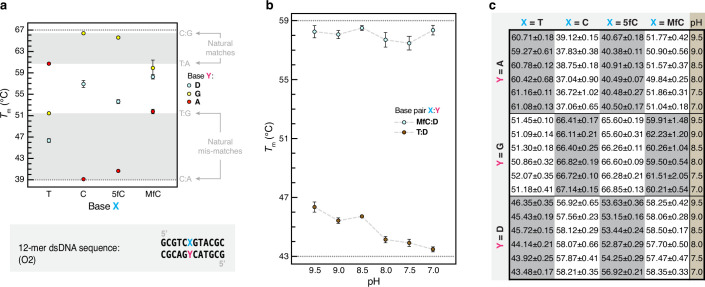
Base pairing properties of the unnatural bases MfC and D. Assessment of thermodynamic base pair stabilities by DNA duplex melting experiments in a second DNA duplex sequence (sequence O2; complementing Fig. 3). **a**: Melting temperatures (*T*_*M*_) of DNA 12-mer duplexes possessing varying pyrimidine:purine (X:Y) pairings measured at pH 9.5, with indication of temperature ranges for canonical matched and mismatched base pairs. **b**: pH dependence of melting temperatures for **MfC**:**D** and T:**D** pairings between pH 9.5 and pH 7.0. **c**: Tabulated melting temperatures for all X:Y pairings shown in **a** ([°C]), dependent on pH. The means of three independent experiments are shown with s.d. (n = 3), respectively. Source data

Comparable results were obtained with a second 12-mer DNA duplex (O2; Extended Data Fig. 1) that featured a higher GC content and the permutated base pair (X:Y) in a more central position, providing an **MfC**pG context in which 5fC is typically found in mammalian DNA. The O2 duplexes with fully complementary sequences were more stable than those of O1 at pH 9.5 (Extended Data Fig. 1a), but, as with O1, the **MfC**:**D** duplex (*T*_m_ = 58.3 ± 0.4 °C) and the **MfC**:G double strand (*T*_m_ = 59.9 ± 1.5 °C) melted slightly below the *T*_m_ of the T:A duplex (60.7 ± 0.2 °C). In contrast, the O2 T:**D** double strand was even less stable (*T*_m_ = 46.4 ± 0.4 °C at pH 9.5) than the O1 T:**D** duplex (*T*_m_ = 51.2 ± 0.4 °C; Fig. 3b), and it was further destabilized upon pH reduction (*T*_m_ = 43.5 ± 0.2 °C at pH 7.0; Extended Data Fig. 1b). No pH trend was found for the **MfC**:**D** duplex nor any other base-pair combinations in this case (Extended Data Fig. 1b,c).

These thermal melting experiments support our design rationale and confirm that protonation of **D**, even under basic conditions, enables formation of a stable **MfC**:**D** pair and destabilizes the alternative T:**D** Watson–Crick ‘mismatch’.

## DNA polymerases pair dDTP selectively with MfC at neutral pH

**Fig. 4:**
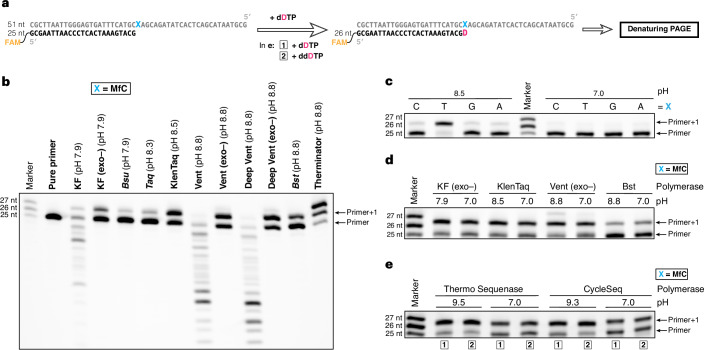
Compatibility of dDTP and template MfC with DNA polymerases and their selectivity. **a**, Schematic overview of the design of single-nucleotide incorporation experiments (oligonucleotide sequences and buffer conditions are presented in Supplementary Tables 1 and 2). **b**, Screen for polymerases to incorporate **dDTP** opposite **MfC** at alkaline pH (supplier-recommended). **c**, Undesired incorporation of **dDTP** opposite canonical template bases by KlenTaq polymerase at alkaline or neutral pH (reaction time: 30 s at pH 9.5, 90 s at pH 7.0). Extended Data Fig. 2a–c presents further polymerases. **d**, Incorporation of **dDTP** opposite **MfC** by DNA polymerases at alkaline and neutral pH. **e**, Incorporation of **dDTP** (**1**) and **ddDTP** (**2**) opposite **MfC** by Sanger sequencing polymerases, at alkaline or neutral pH. Primer+1 denotes primer extended by one nucleotide. Results of individual experiments are shown (*n* = 1, representative of two independent experiments with similar results), respectively. See Source data for further information regarding the uncropped and unprocessed images of the gels in **b**–**d**. Source data

To establish whether the cytosine modification-specific unnatural base pair is compatible with DNA polymerases, we explored DNA primer extension by the incorporation of 3,7-dideaza-2′-deoxyadenosine 5′-triphosphate (**dDTP**) templated by **MfC**. We first synthesized **dDTP** from **dD** by monophosphorylation followed by morpholidate activation and pyrophosphate substitution to install the 5′-triphosphate moiety^52,53^ (Fig. 2). We then performed single-nucleotide incorporation experiments using a 25-mer 5′-fluorescently labelled primer hybridized to a 51-mer template strand, with **MfC** as the first nucleotide after the primer binding site (Fig. 4a and Supplementary Table 1). We evaluated **MfC**-templated incorporation of **dDTP** by various DNA polymerases commonly employed for PCR (Fig. 4b). Several polymerases, each lacking 3′-to-5′ exonuclease activity, achieved primer elongation at their optimal alkaline pH (pH 7.9–8.8; Supplementary Table 2). These included the Klenow fragment of *Escherichia coli* polymerase I (KF (exo–)) as well as the thermostable KlenTaq, Vent (exo–), and *Bst* polymerases. This confirmed **dDTP** was a viable DNA polymerase substrate for incorporation opposite **MfC**. Notably, this represents an example of successful polymerase-mediated primer extension with a 3,7-dideazapurine building block^54^.

**Extended Data Fig. 2:**
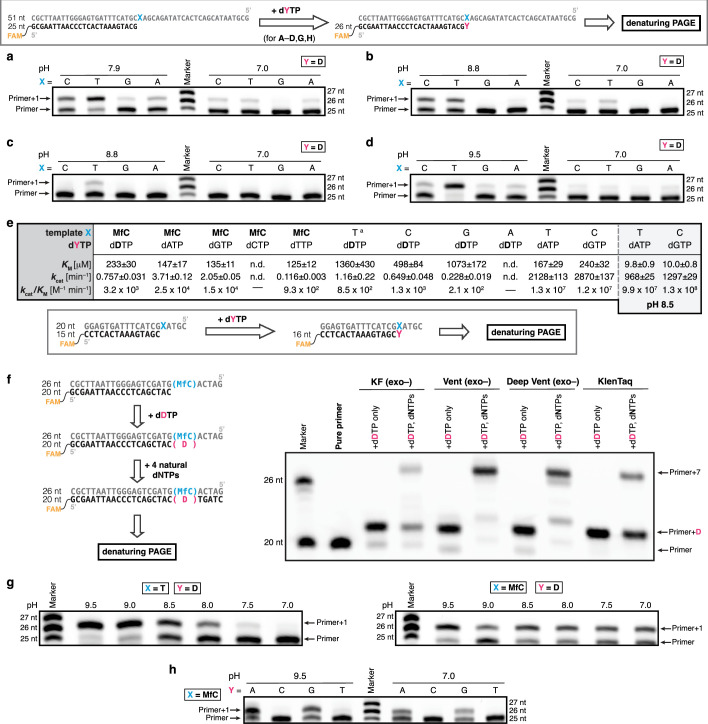
pH influence on polymerase selectivity and processivity in the presence of MfC and dDTP. **a**–**d**: Undesired incorporation of **dDTP** opposite canonical template bases by various polymerases at alkaline or neutral pH. **a**: KF (exo–) polymerase. **b**: Vent (exo–) polymerase. **c**: Bst polymerase. **d**: Thermo Sequenase. **e**: Michaelis-Menten steady-state kinetics of dNTP incorporation by KlenTaq polymerase at pH 7.0. *K*_M_ and *k*_cat_ values were determined by nonlinear regression based on 8–10 dNTP concentrations (5–1500 μM; means of three independent experiments with s.d. used for each concentration; n *=* 3; Supplementary Fig. 1). ^a^ As the protonation of **dDTP** has a big influence on its incorporation opposite T, apparent turnover rates determined at high **dDTP** concentrations (at which a substantial concentration of unprotonated **dDTP** is present even at pH 7.0), and thus the corresponding *k*_cat_, are likely inflated. n.d.: not determined because the polymerase reaction was too slow to accurately determine *K*_M_ and *k*_cat_ under Michaelis-Menten conditions. Values for the canonical pairings T:dATP and C:dGTP at the recommended pH 8.5 (highlighted in grey) are included for reference. **f**: Full primer extension beyond the unnatural **MfC**:**D** base pair by various polymerases at pH 7.0. **g**: **dDTP** incorporation by Thermo Sequenase between pH9.5–7.0. Incorporation opposite T (left) and **MfC** (right). **h**: Incorporation of canonical dYTPs opposite **MfC** by Thermo Sequenase. Results of individual experiments are shown (n *=* 1; representative for two independent experiments with similar results: **a**–**d**,**f**; single, unrepeated experiments: **g**-**h**). See readme source file for further information regarding the uncropped and unprocessed images of the gels in panels **a**-**d**,**g**-**h**. Source data

The polymerase-catalysed primer extension of DNA, in applications such as sequencing or PCR, typically involves the polymerase having access to a pool of dNTPs, each of which can initially bind the polymerase•DNA template–primer complex in rapid equilibrium. In the presence of a ‘correct’ dNTP, transition into the catalytically active ‘closed’ state is highly favoured, and nucleotidyl transfer occurs rapidly upon adoption of the Watson–Crick geometry. In contrast, an ‘incorrect’ substrate strongly slows this process^38,55,56^. We thus examined polymerase specificity when processing **dDTP** in the presence of each canonical template base. Due to its A-like hydrogen-bonding potential, unprotonated **D** can pair with T, whereas its protonated form (p*K*_a_ ≥ 8.6) should not, as demonstrated by our thermal melting experiments (Fig. 3). Indeed, the polymerases that had extended a primer with **dDTP** opposite **MfC** (Fig. 4b) readily incorporated **dDTP** opposite T at their optimal alkaline pH (pH 7.9–8.8) but not at pH 7.0 (Fig. 4c and Extended Data Fig. 2a–c). Primer elongation opposite the remaining canonical template bases was markedly less favourable at both pH 9.5 and pH 7.0. Crucially, however, pH reduction to pH 7.0 did not substantially change **dDTP** incorporation opposite **MfC** (Fig. 4d). This indicates that protonation of **D** generally determines polymerase selectivity.

These findings were supported by steady-state kinetic experiments (Extended Data Fig. 2e and Supplementary Fig. 1) at pH 7.0 with KlenTaq polymerase, which had previously been examined with the **MfC**:dATP substrate pair^46^. The catalytic efficiency (*k*_cat_/*K*_M_) for **dDTP** incorporation opposite **MfC** (3.2 × 10^3^ M^−1^ min^−1^) was lower, and the *K*_M_ (233 ± 30 μM) higher, than those of **MfC**-paired dATP (*k*_cat_/*K*_M_ = 2.5 × 10^4^ M^−1^ min^−1^, *K*_M_ = 147 ± 17 μM) and dGTP (*k*_cat_/*K*_M_ = 1.5 × 10^4^ M^−1^ min^−1^, *K*_M_ = 135 ± 11 μM), because the absence of N3 as a minor groove hydrogen-bond donor in **D** probably influences **dDTP** recognition in ways that might impair the polymerase reaction. However, **dDTP** incorporation opposite the canonical bases, including T (*k*_cat_/*K*_M_ = 8.5 × 10^2^ M^−1^ min^−1^, *K*_M_ = 1,360 ± 430 μM), was even less favourable. Additionally, **dDTP** incorporation opposite T and C (*k*_cat_/*K*_M_ = 1.3 × 10^3^ M^−1^ min^−1^) was markedly less efficient than the natural, cognate incorporation of dATP (*k*_cat_/*K*_M_ = 1.3 × 10^7^ M^−1^ min^−1^) and dGTP (*k*_cat_/*K*_M_ = 1.2 × 10^7^ M^−1^ min^−1^), respectively. Thus, although unnatural **dDTP** is a more challenging substrate than canonical dNTPs, KlenTaq showed selectivity towards pairing it with **MfC** at pH 7.0, in line with our single-nucleotide incorporation experiments (Fig. 4c,d). These experiments provided a kinetic insight into the properties of the unnatural **MfC**:**D** base pair.

We further assessed continuous primer extension beyond the unnatural base pair at pH 7.0 (Extended Data Fig. 2f). In a two-step procedure, polymerases were first exposed to a primer•template duplex and **dDTP** to elicit single-nucleotide incorporation opposite **MfC**. Then, the four canonical dNTPs were added to fully extend the primer. All tested polymerases produced full-length product, demonstrating that primer extension past the **MfC**:**D** base pair can be achieved. The most efficient extension was observed with Vent (exo–) and Deep Vent (exo–) polymerases, as they are reportedly largely unaffected by the absence of N3 in dNTP substrates^57^.

Encouraged by these results, we next investigated the utility of the **MfC**:**D** base pair for epigenetic sequencing, adopting the Sanger sequencing workflow. This approach identifies the nucleobase sequence from selective termination of primer elongation by low-level incorporation of specific 2′,3′-dideoxynucleoside 5′-triphosphates (ddNTPs) in the presence of all canonical dNTPs^58^. Previously, unnatural base pairs have been detected via Sanger sequencing without the requirement to use an unnatural ddNTP, by using one of two distinct approaches. In one approach, incorporation of an unnatural dNTP opposite its unnatural complementary base (or an abasic site) can itself lead to chain termination at this site^59^. In an alternative approach, if the unnatural base pair is highly orthogonal to the canonical base pairs^40,60,61^, intentional omission of the unnatural dNTP from the reaction solution can lead to chain termination at the unnatural template base. Our approach is unlike the first of these concepts because full primer extension beyond the **MfC** template base can be achieved in the presence of **dDTP** (Extended Data Fig. 2f), which makes it similar to the second concept. However, we do need to use the chain terminator 3,7-dideaza-2′,3′-dideoxyadenosine 5′-triphosphate (**ddDTP**) in our sequencing experiments because primer extension also occurs in the absence of **dDTP** as previously reported^20^.

Given ddNTPs can be poor substrates for native DNA polymerases^62^, we examined the incorporation of **dDTP** and **ddDTP** by polymerases optimized for Sanger sequencing. **ddDTP** was synthesized from chlorinated intermediate **4** via Barton–McCombie deoxygenation prior to amination and phosphorylation^47^ (Fig. 2). We identified two polymerases that incorporated **dDTP** and **ddDTP** opposite **MfC** under the standard alkaline conditions for these Sanger sequencing polymerases (pH 9.3–9.5; Fig. 4e and Supplementary Table 2). Thermo Sequenase, a truncated variant of *Taq* polymerase^63^, accommodated the unnatural base pair most efficiently. Like the related KlenTaq polymerase (Fig. 4c,d), Thermo Sequenase incorporated **dDTP** opposite T at its optimal basic pH but not at pH 7.0 (Extended Data Fig. 2d,g), whereas **dDTP** (and **ddDTP**) incorporation opposite **MfC** was independent of pH (Fig. 4e and Extended Data Fig. 2g). In line with previous reports^20,46^, Thermo Sequenase also paired **MfC** with both dATP and dGTP in the absence of **dDTP** (Extended Data Fig. 2h).

**Fig. 5:**
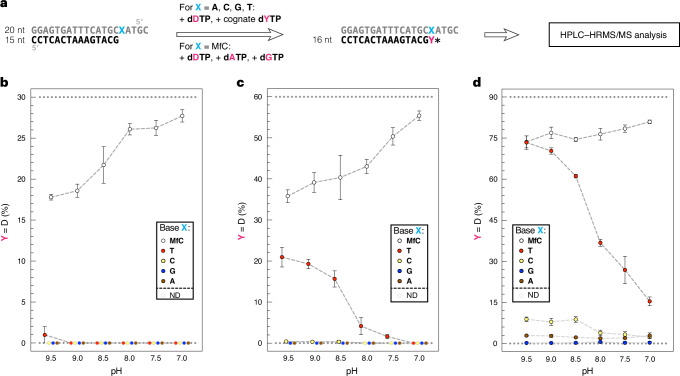
Competitiveness of dDTP against the canonical dYTPs as polymerase substrate. **a**, Schematic overview of the design for dNTP competition experiments (some 17-mer elongation products were also observed (*), particularly at higher **dDTP** concentrations, and included in the analysis; Supplementary Table 3). **b**, Proportions of **dDTP** elongation products in the presence of 1 equiv. **dDTP**. **c**, Proportions of **dDTP** elongation products in the presence of 10 equiv. **dDTP**. **d**, Proportions of **dDTP** elongation products in the presence of 100 equiv. **dDTP**. Percentages are normalized to the total amount of elongated primer. Means of 3 independent experiments are shown with s.d. (*n* = 3) (ND, not detected = <0.2%). Percentages of corresponding dATP and dGTP incorporation products for X = **MfC** templates are shown in Extended Data Fig. 3a,c,d. Source data

However, incorporation of the more favourable polymerase substrates dATP and dGTP, featuring a minor groove hydrogen-bond acceptor (N3), at **MfC** during Sanger sequencing could suppress **ddDTP** termination at true modified cytosine positions, whereas erroneous **ddDTP** incorporation at canonical template bases could produce false-positive signals. Other applications, such as PCR, would also be impacted by such undesired incorporation activity. Accordingly, we next analysed the substrate preferences of Thermo Sequenase in dNTP competition experiments (Fig. 5a), dependent on pH (pH 9.5–7.0). To assess incorporation specificity at **MfC** sites, we provided the polymerase with an equimolar mixture of dATP, dGTP and **dDTP**. Primer extension products were then identified and relatively quantified via HPLC–HRMS/MS (Supplementary Table 3 and Supplementary Figs. 2–18), similar to peptide characterization workflows^64^. We monitored potential undesired **dDTP** incorporation in dNTP competition reactions with the canonical template bases, supplying only the respective canonical cognate dNTP and **dDTP**.

**Extended Data Fig. 3:**
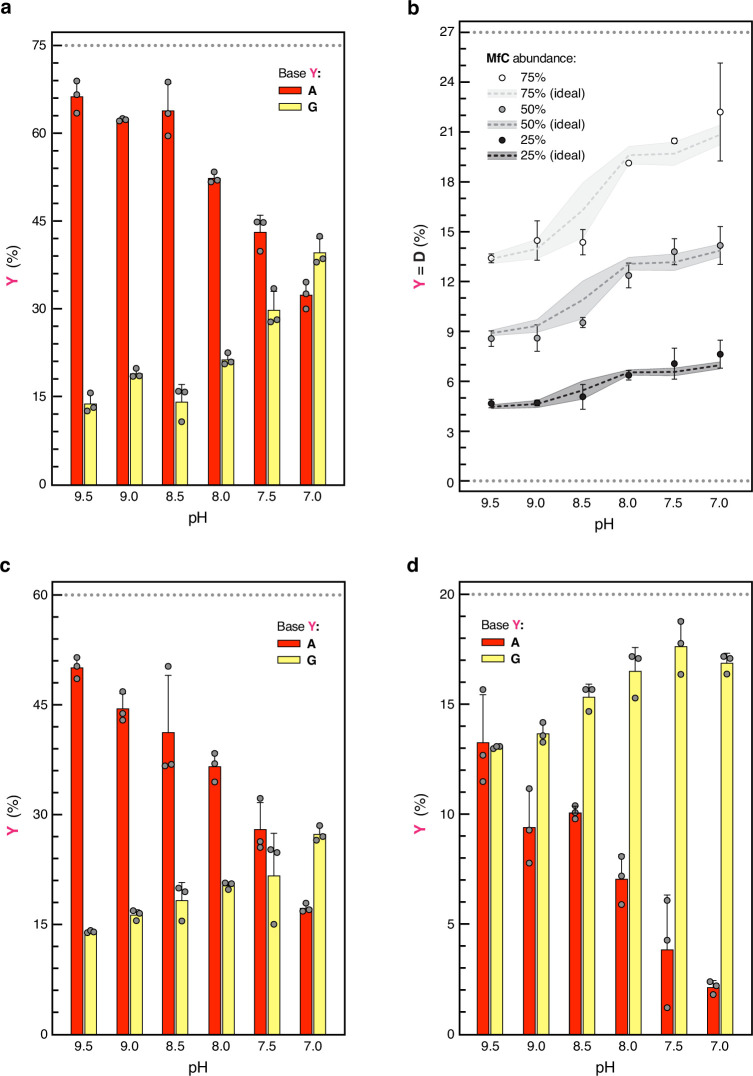
Competition of dDTP, dATP and dGTP for incorporation opposite MfC depending on pH. **a**: Incorporation of dATP and dGTP in the presence of 1 equivalent **dDTP** (see Fig. 5b). **b**: Incorporation of **dDTP** (1 equivalent) in the presence of an **MfC** template diluted with a C template; dashed lines represent expected results, based on the **dDTP** incorporation data with undiluted **MfC** (Fig. 5b; mean values), multiplied by the dilution factor (0.75, 0.5, 0.25, respectively); grey areas around the dashed lines represent the error of the **dDTP** incorporation data with undiluted **MfC** (Fig. 5b), multiplied by the dilution factor (0.75, 0.5, 0.25, respectively). **c**: Incorporation of dATP and dGTP in the presence of 10 equivalents **dDTP** (see Fig. 5c). **d**: Incorporation of dATP and dGTP in the presence of 100 equivalents **dDTP** (see Fig. 5d). Means of three independent experiments are shown with s.d. (n = 3), respectively. Source data

At pH 9.5, **dDTP** was incorporated at 17.8 ± 0.3% opposite **MfC** (Fig. 5b), with the rest of the primer being extended by dATP and dGTP (Extended Data Fig. 3a). Interestingly, **dDTP** incorporation became progressively more favourable upon lowering the pH, and rose to 27.7 ± 0.8% at pH 7.0 (Fig. 5b), at which **dDTP** incorporation was not observed opposite the canonical template bases (Fig. 5b), as was suggested by our steady-state experiments (Extended Data Fig. 2e). In line with our base-pair design, the increase in **dDTP** protonation might improve its pairing with the **MfC** template base in Watson–Crick geometry during polymerase incorporation in the enzyme active site, accelerating the reaction relative to the competing incorporation of dATP or dGTP. Notably, pH reduction to pH 7.0 also made dGTP incorporation more favourable (Extended Data Fig. 3a). This is consistent with a protonation event, such as the protonation of **MfC** to give a three-hydrogen-bonded Watson–Crick base pair with dGTP (Fig. 3a), also influencing substrate recognition by the polymerase. When the **MfC** template was mixed with a template featuring unmodified C in different ratios, **dDTP** incorporation was found to be reduced following the expected dilution trend (Extended Data Fig. 3b). This further supports that **dDTP** is only competitive for incorporation opposite **MfC** and not C under these conditions.

We then examined whether **dDTP** incorporation opposite (undiluted) **MfC** could be improved by raising its concentration relative to the natural dNTPs. Indeed, a tenfold higher **dDTP** concentration led to increased primer extension with **dDTP** opposite **MfC** across the entire pH range (pH 9.5–7.0), and it was dominant over dATP and dGTP incorporation at pH 7.0 (55.4 ± 1.1% **dDTP**; Fig. 5c and Extended Data Fig. 3c). Simultaneously, **dDTP** misincorporation at the canonical bases remained undetectable at neutral pH (Fig. 5c). This selectivity, also indicated by the *k*_cat_/*K*_M_ values obtained for KlenTaq polymerase (Extended Data Fig. 2e), is probably supported by the reduced favourability of the **dDTP** substrate, which lacks N3. **dDTP** misincorporation, at pH 7.0, was only observed when raising its concentration 100-fold (Fig. 5d and Extended Data Fig. 3d).

Overall, Thermo Sequenase can selectively incorporate **dDTP** opposite **MfC** at pH 7.0, at high levels that can be influenced through the dNTP concentration ratio. This suggests that Thermo Sequenase can be deployed to generate unambiguous **ddDTP** termination signals during Sanger sequencing in the presence of **MfC**, given it has been engineered to incorporate dNTPs and ddNTPs at similar rates^63^.

## Sanger sequencing of 5fC using the unnatural base pair

**Fig. 6:**
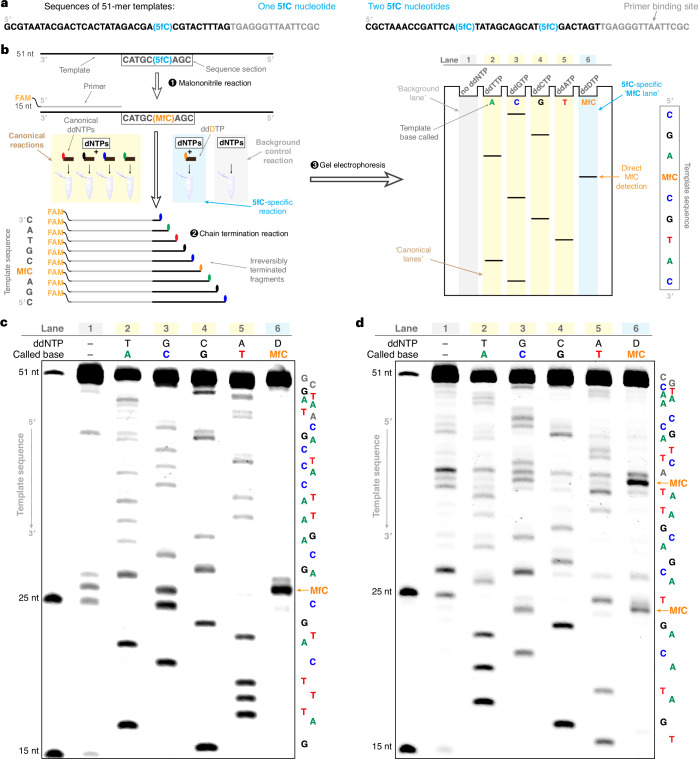
Sanger sequencing of 5fC-containing oligonucleotides with the unnatural MfC:D base pair. **a**, Sequences of 51-mer DNA templates. **b**, Overview of the design of a six-lane Sanger sequencing experiment: malononitrile treatment of the template (part of the sequence of the first template from **a** is shown for illustration), six parallel primer elongation reactions with Thermo Sequenase (coloured ellipses represent base-specific 3′-termination), and product separation by PAGE enable readout of the five-letter template sequence. **c**, Sanger sequencing gel for a template containing one 5fC base at position 26 (T3, Supplementary Table 1). The template sequence without primer binding site is shown next to the gel (grey bases: not callable from gel). **d**, Sanger sequencing gel for a template containing two 5fC bases at positions 24 and 36 (T4, Supplementary Table 1). Concentrations of dNTPs and ddNTPs: 37.5 μM dATP, 75 μM dCTP, 150 μM dGTP, 75 μM dTTP; 1.125 μM ddATP, 2.25 μM ddCTP, 4.5 μM ddGTP, 3.0 μM ddTTP, 112.5 μM **ddDTP**. Results of individual experiments are shown (*n* = 1, representative of 2 independent experiments with similar results). Source data

**Extended Data Fig. 4:**
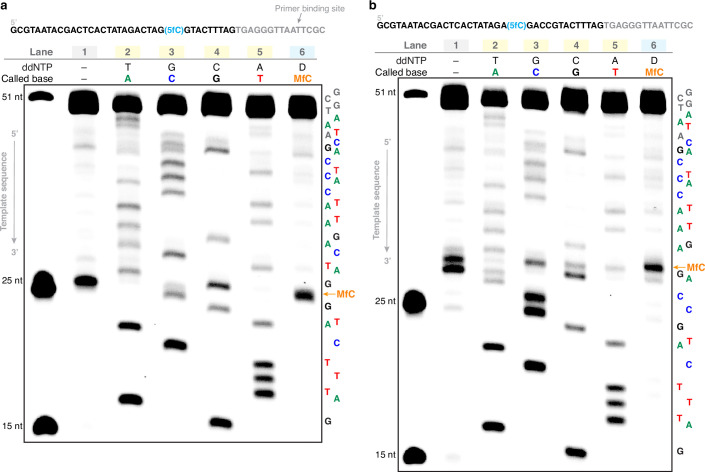
Sanger sequencing of 5fC-containing oligonucleotides implementing the unnatural MfC:D base pair in different (5fC)pG sequence contexts. **a**: Sanger sequencing gel for a template containing one 5fC nucleotide at position 25 (T9, Supplementary Table 1). **b**: Sanger sequencing gel for a template containing one 5fC nucleotide at position 29 (T10, Supplementary Table 1). Concentrations of dNTPs and ddNTPs: 37.5 mM dATP, 75 mM dCTP, 150 mM dGTP, 75 mM dTTP; 1.125 mM ddATP, 2.25 mM ddCTP, 4.5 mM ddGTP, 3.0 mM ddTTP, 112.5 mM **ddDTP**. Results of individual experiments are shown (n *=* 1; representative for two independent experiments with similar results), respectively. Source data

We then demonstrated direct 5fC sequencing using the unnatural base pair in a Sanger format. A DNA 51-mer oligonucleotide containing either one or two 5fC nucleotides (Fig. 6a and Extended Data Fig. 4) was treated with malononitrile, primed with a 15-mer 5′-fluorescently labelled DNA molecule, and then subjected to primer elongation by Thermo Sequenase in the presence of the four canonical dNTPs in five parallel extension reactions (Fig. 6b). Each reaction included either ddTTP, ddGTP, ddCTP, ddATP or **ddDTP** as chain terminator. Termination products were then separated and visualized by polyacrylamide gel electrophoresis (PAGE). An additional control reaction was performed with the dNTP mix alone (lane 1). The template sequence, in the 5′-to-3′ direction, can be determined following the gel ‘ladder’ from top to bottom. Lanes 2–5 allow readout of the canonical bases, and lane 6 serves as the **MfC**-specific lane indicating 5fC positions.

To ensure that strong **ddDTP** termination occurred selectively at true **MfC** sites, the reactions were conducted at pH 7.0 and at an optimized **ddDTP** concentration to suppress misincorporation opposite T (Supplementary Fig. 19). At this pH, dATP incorporation at the unnatural base caused polymerase pausing, as confirmed by HPLC–HRMS (Supplementary Figs. 20 and 21). The associated ddNTP-independent termination around **MfC** positions was thus minimized by adjusting concentrations in the dNTP mix (Supplementary Fig. 22) to increase dGTP incorporation at **MfC**.

Applying these optimized conditions, we achieved sequencing of templates containing a single (Fig. 6c and Extended Data Fig. 4a,b) or two (Fig. 6d) 5fC bases, at different positions and with distinct flanking sequences. **MfC** was unambiguously identified via strong and selective **ddDTP** termination signals at the correct positions (lane 6). Because Thermo Sequenase favourably paired **MfC** with dGTP at pH 7.0 (Extended Data Fig. 3a,c,d) and the dGTP concentration in the reaction was increased, **MfC** also caused a degree of ddGTP termination (lane 3). All other bands in the **MfC** lane (lane 6), around the **MfC** site caused by dATP misincorporation, and the faint bands that could be low-level DNA damage artefacts are ddNTP-independent as they also occurred in the control lane (lane 1).

Importantly, our approach allowed the successful identification of two 5fC bases within the same template (Fig. 6d) that had not been achieved in previous attempts at DNA lesion sequencing via unnatural base pairs^39–41^. Our results suggest that this strategy can be employed to successively detect multiple modified base positions within one DNA template.

Overall, these experiments illustrate that the unnatural **MfC**:**D** base pair can be utilized to directly and consecutively read an epigenetic cytosine modification via a polymerase-based sequencing approach.

## Epigenetic sequencing using the unnatural base pair strategy

Implementation of the **MfC**:**D** base pair in Sanger sequencing allowed qualitative identification of 5fC in synthetic oligonucleotides, to demonstrate proof-of-principle utility of an unnatural base pair to sequence an epigenetic DNA base modification. There are key areas where the system would need further development to enable practical utility. An engineered polymerase with improved fidelity and efficiency of copying sample DNA using the unnatural base pair together with further structural optimization of the **MfC**:**D** pair would enable PCR amplification of biological sample DNA, which has been achieved for other unnatural base pairs^24,26,27^. This would allow us to generate a sufficient quantity of DNA while preserving the unnatural base pair for sequencing via standard sequencing platforms, such as automated Sanger sequencing and NGS. For modern fluorescence capillary Sanger sequencing, the unnatural ddNTPs would need suitable fluorescent labelling at wavelengths compatible with the detection system and the other labelled ddNTPs. NGS would require an unnatural dNTP to be adapted with a reversible 3′-*O*-protecting group and the attachment of a suitable fluorophore via a cleavable linker^65^. The unnatural base pair strategy may be particularly suited to the emerging single-molecule ‘Sequencing by Expansion’ (SBX) technology (Roche)^66^, for which it would be necessary to synthesize the appropriate expanded dNTP for the unnatural base. Note that SBX would not require PCR amplification and it has the potential to detect low levels of base modifications in DNA via unnatural base pairs. It should be possible to combine the sequencing of 5fC via the **MfC**:**D** pair (Fig. 1b) with detection of one other epigenetic mark, such as 5mC, 5hmC or 5caC, via a C-to-T transition^15–18,67^ (Fig. 1a). Given the availability of selective chemistry to convert both 5mC and 5hmC into 5fC^42–45^, the principle of **MfC**:**D** pairing could also be applicable to sequence any one of these more abundant derivatives. To simultaneously sequence all four epigenetic derivates of C would necessitate the development of four orthogonal pairing combinations.

## Conclusions

We have demonstrated the development and use of a cognate unnatural base pair between the chemically transformed 5fC derivative **MfC** and the rationally designed protonated unnatural base **D** to enable single-nucleotide-resolution sequencing of 5fC along with A, C, G and T. This represents a distinct example of exploiting unnatural base pairing to sequence an epigenetic DNA base modification. As an approach that is differentiated from other methods to detect epigenetic modifications, it could be combined with established methods to read more than one modification at a time. By considering other unnatural base-pairing combinations, the general concept has the potential to be adapted and applied more broadly to detect modifications of DNA and RNA nucleobases.

## Methods

### Oligonucleotides

The sequences of all used templates and primers are listed in Supplementary Table 1. All templates containing only the four natural unmodified nucleobases were purchased from IDT as HPLC-purified oligonucleotides. The 15-mer and 25-mer primers were purchased with a 5′-FAM label from IDT as HPLC-purified oligonucleotides. The 51-mer 5fC-containing templates for single-nucleotide incorporation experiments and Sanger sequencing experiments were purchased from ATDBio as HPLC-purified oligonucleotides. The 20-mer 5fC-containing template was synthesized on an Applied Biosystems 394 DNA/RNA Synthesizer, using the 5-Formyl-dC III CE Phosphoramidite (Glen Research), and purified by HPLC. Concentrations were determined on a NanoDrop One system (Thermo Scientific).

The 12-mer 5fC-containing template for melting experiments was purchased from ATDBio as double HPLC-purified oligonucleotide. The 12-mer **D**-containing template was synthesized by ATDBio with ultra-mild phosphoramidites, including **dD** phosphoramidite **1** (synthesized in-house), 10-min coupling time and deprotection using ammonia and heating of the oligo at 55 °C for 1 h. Concentrations were determined on an Agilent Cary 3500 UV–vis spectrophotometer.

### (Di)deoxynucleoside triphosphates

Natural dNTPs were purchased from Jena Biosciences as 100 mM aqueous solutions (sodium salt) and diluted with water to give 10 mM stock solutions, then stored at −20 °C. Natural ddNTPs were purchased from Jena Bioscience as 10 mM aqueous solutions (lithium salt) and stored at −20 °C. These stock solutions were then freshly diluted with water to give appropriate 10× working solutions for each experiment. The unnatural compounds **dDTP** and **ddDTP** were synthesized as outlined in Supplementary Information, dissolved in storage buffer (20 mM Tris-HCl pH 8.6, 1 mM ethylenediaminetetraacetic acid (EDTA)) to give 10 mM stock solutions, and stored at −80 °C. These were then freshly diluted with water to give appropriate working solutions for each experiment.

### 5-Formylcytosine modification reaction with malononitrile

According to a published protocol^20^, typically, the fC-containing oligonucleotide was diluted in 50 mM ammonium acetate, pH 7.0, and 30,000 equiv. of malononitrile (Thermo Scientific) were added (as a 1.5 M solution in water). The reaction mixture was incubated in a thermoshaker at 37 °C and 1,000 r.p.m. for 21 h, then purified by HPLC, lyophilized and redissolved in water. The concentration was determined on a NanoDrop One system (Thermo Scientific). Full conversion was confirmed by HPLC-electrospray ionization (ESI^−^)-HRMS/MS analysis.

### DNA duplex melting experiments

Typically, the forward and reverse oligonucleotides (3 pmol each) were annealed in 100 μl of buffer containing 10 mM NaH_2_PO_4_ and 200 mM NaCl at the desired pH. The duplex DNA was annealed via heating at 95 °C for 2 min and then cooling to 4 °C at a rate of 2 °C min^−1^. The annealed samples were transferred to quartz cuvettes (path length 10 mm) and covered with mineral oil (300 μl) to prevent evaporation. Technical triplicates of DNA duplex melting curves were measured via UV–vis spectroscopy on an Agilent Cary 3500 UV–vis spectrophotometer (with a Multicell Peltier element), by heating the samples from 20 °C to 80 °C at a rate of 5 °C min^−1^ while recording the absorbance at 260 nm. UV–vis data were processed in RStudio software, and melting temperatures were calculated by Prism 10 software by approximation using a nonlinear fit (sigmoidal, 4PL, X is concentration) with symmetrical approximate confidence interval. Melting temperatures are presented as mean values with s.d., of three independent replicates.

### General procedure for polymerase experiments

Primer and a slight excess of template (Supplementary Table 1) were annealed in an 8-μl or 7-μl volume, including 1 μl of a 10× reaction buffer solution, as recommended by the supplier (Supplementary Table 2) and adjusted to the desired pH. Annealing was achieved via heating at 80 °C for 2 min followed by cooling to 4 °C at a rate of 0.1 °C s^−1^. The desired (d)dNTPs (1 μl of a 10× stock solution in water, respectively) were added to the appropriate concentration followed by the respective amount (in units, U) of DNA polymerase (1 μl of respective stock). The final 10-μl reaction volume was then incubated at either 37 °C or 60 °C (for thermostable polymerases) and was stopped after the desired time either by adding 10 μl of EDTA-containing gel loading buffer (Gel Loading Buffer II, Invitrogen) for denaturing PAGE (1× TBE buffer, 22-cm gel length/0.75-mm gel thickness, 16 W constant) or by the addition of 5 μl of 100 mM EDTA (pH 8.0) for HPLC-ESI^−^-MS/MS analysis. Gels were imaged on a BioRad ChemiDoc MP imager (Alexa 488 Blot settings).

### Single-nucleotide incorporation experiments

Typically, a 5′-FAM-labelled 25-mer primer (4 pmol) was annealed to a 51-mer template (5 pmol) that contained the target nucleobase at position 26, in an 8-μl volume. The desired (d)dNTP was added to a final concentration of 25 μM, followed by 0.5 U of the respective DNA polymerase (1 U for CycleSeq polymerase). Generally, reactions with **MfC**-containing templates were incubated for 5 min. For **dDTP** incorporation experiments opposite natural template bases (Fig. 4c and Extended Data Fig. 1), incubation times were as follows: KF (exo–) polymerase (0.1 U, 30 s); Vent (exo–) polymerase (0.2 U, 30 s); *Bst* polymerase (0.5 U, 2 min at pH 8.8, 5 min at pH 7.0); Thermo Sequenase (0.5 U, 30 s at pH 9.5, 90 s at pH 7.0). After reaction quenching, the oligonucleotide mixture was analysed by 12% denaturing PAGE.

### Michaelis–Menten steady-state kinetic experiments

Typically, a 5′-FAM-labelled 15-mer primer (4 pmol) was annealed to a 20-mer template (5 pmol) that contained the desired template base X at position 16, in a 4-μl volume at pH 7.0 (or pH 8.5) and incubated at 60 °C with KlenTaq polymerase in a 5-μl volume for 5 min. A pre-heated (60 °C) solution of the desired dNTP substrate (5 μl) was added, and samples were taken from the resulting 10-μl reaction volume after the desired reaction times. The oligonucleotide mixtures were analysed by 12% denaturing PAGE. The amount of enzyme used (2.5–250 nM), the reaction time (1–90 min) and the gradient concentration of dNTP (5–1,500 μM) were adjusted to give reaction extents of 25% or less. Relative primer conversion ratios were calculated based on quantification of the corresponding gel band intensities using ImageJ (version 1.53t). For the combinations **MfC**:dATP, **MfC**:dGTP, **MfC**:**dDTP**, T:**dDTP** and C:**dDTP**, initial velocities (*v*_0_) were calculated from the slope of timecourse experiments with six data points. For the combinations **MfC**:dTTP, G:**dDTP**, T:dATP and C:dGTP, initial velocities were calculated as the ratio of converted primer and the reaction time based on individual time points. All experiments were performed as three independent replicates. The initial velocities were then normalized to the enzyme concentration used in each experiment to give the apparent turnover rates (*k*_cat, app_; Supplementary Fig. 1). These data were then fit to the Michaelis–Menten equation (${k}_{\rm{{cat},{app}}}={\frac{{k}_{\rm{cat}}[S]}{{K}_{\rm{M}}+[S]}}$, where [*S*] is substrate concentration) via nonlinear regression (performed in GraphPad Prism 10) to obtain *K*_M_ and *k*_cat_ (for all fits: *R*^2^ > 0.91).

### Primer extension experiments

Typically, a 5′-FAM-labelled 20-mer primer (4 pmol) was annealed to a 26-mer template (5 pmol) that contained **MfC** at position 21, in a 6-μl volume. **dDTP** was added to a final concentration of 100 μM, followed by 2 U of the respective DNA polymerase. The reaction mixture was then incubated at 60 °C (37 °C for Klenow (exo–)) for 20 min, after which a 3-μl sample was taken. To the remaining reaction solution was added a mix of the four canonical dNTPs (final concentration 100 μM), followed by another 2 U of polymerase. The reaction mixture was incubated for another 3 h. The oligonucleotide mixtures were analysed by 12% denaturing PAGE.

### dNTP incorporation competition experiments

Typically, a 5′-FAM-labelled 15-mer primer (2.5 pmol) was annealed to a 20-mer template (3 pmol) that contained the target nucleobase at position 16, in a 7-μl volume. The desired natural dNTP (for experiments with **MfC**-containing templates, an equimolar mix of dATP and dGTP) was added to a final concentration of 10 μM each. Unnatural **dDTP** was added to a final concentration of 10 μM, 100 μM or 1 mM (1, 10 or 100 equiv. relative to dNTP), respectively. After addition of 0.5 U of Thermo Sequenase, the reaction mixtures were incubated at 60 °C and the reaction was stopped after 1 min (natural templates) or 5 min (**MfC**) with EDTA and diluted with 15 μl of buffer A (100 mM 1,1,1,3,3,3-hexafluoro-2-propanol (HFIP), 30 mM triethylamine in water, pH 9.0) for HPLC-ESI^−^-HRMS/MS analysis. Reactions with a template containing one of the canonical target bases included the corresponding complementary dNTP and **dDTP**, whereas reactions with **MfC**-containing templates included dATP, dGTP and **dDTP**. Dilutions of the **MfC** template base were performed with a mix of an **MfC**-containing and a C-containing template.

### HPLC-ESI^−^-HRMS/MS analysis

HPLC-ESI^−^-HRMS/MS analysis of polymerase reaction samples was performed on an Orbitrap Exploris 120 system (Thermo Scientific) coupled to a Vanquish HPLC system (Thermo Scientific), with mixtures of 100 mM HFIP, 30 mM triethylamine in water, pH 9.0 (buffer A) and methanol (buffer B) as solvents. Product separation was achieved on a DNAPac RP column (2.1/100 mm, 4 μm; Thermo Scientific) with the following gradient of buffer B in buffer A at a flow of 0.5 ml min^−1^ (column temperature 80 °C): 2% B for 2.0 min, then 2% to 10% B over 12.0 min (followed by a washing and equilibration step: 10% to 70% B over 0.5 min; 70% B for 2.5 min; 70% to 2% B over 0.5 min; 2% B for 2.5 min; total method duration 20.0 min). MS/MS analysis was conducted with a full scan (550–2,000 *m*/*z*) in negative ion mode (Orbitrap Resolution 60000) followed by an intensity filter (1.0 × 10^−4^), a dynamic exclusion filter (auto mode) and a data-dependent fragment scan (Orbitrap Resolution 30000) with stepped collision energies (normalized; 14, 16, 18%). Sequence detection and relative quantification of oligonucleotide species was carried out with the Oligonucleotide Analysis tool of the BioPharma Finder software (version 4.1) in the ‘find all masses in the run’ setting, applying an absolute MS signal threshold of 4,000 (signal/noise 3) and a minimum confidence level of 0.70 for sequence matching at 6-ppm mass accuracy. The input sequences for the detected oligonucleotides are listed in Supplementary Table 3. Corresponding MS spectra are shown in Supplementary Figs. 2–13 and representative MS chromatograms are shown in Supplementary Figs. 14–18. Relative amounts of primer elongation products are presented as mean values with s.d. of three independent replicates of polymerase reactions.

### Six-lane Sanger sequencing experiments

Typically, a 5′-FAM-labelled 15-mer primer (18 pmol) was annealed to a malononitrile-treated 51-mer template (24 pmol) in a 24-μl volume, including 6 μl of 10× reaction buffer (Supplementary Table 2) adjusted to pH 7.0. The annealed duplex DNA solution was split into six samples to which a solution of the four natural dNTPs (1 μl of 10× stock) was added at final concentrations of 37.5 μM dATP, 75 μM dCTP, 150 μM dGTP and 75 μM dTTP. To the control sample, 1 μl of water was added. To four samples, one of the four canonical ddNTPs was added each (1 μl of 10× stock) to a total concentration of 3% of the corresponding dNTP (4% for ddTTP). To the last sample, **ddDTP** was added (1 μl of 10× stock) to a total concentration of 112.5 μM (300% of dATP). 2 U of Thermo Sequenase (2 μl of 1 U μl^−1^ stock) were added each and the final 10-μl volumes were incubated at 60 °C for 2 h. The polymerization reaction was stopped by the addition of 10 μl of EDTA-containing gel loading buffer (Gel Loading Buffer II, Invitrogen) and the oligonucleotide mixture was analysed by 12% denaturing PAGE (1× TBE buffer, 22-cm gel length/0.75-mm gel thickness, 16 W constant). Gels were imaged on a BioRad ChemiDoc MP imager (Alexa 488 Blot settings).

### Synthesis of phosphoramidite 1, and (di)deoxynucleoside triphosphates dDTP and ddDTP

Synthetic procedures and spectroscopic characterization for new intermediates and products are described in Supplementary Information.

### Reporting Summary

Further information on research design is available in the Nature Portfolio Reporting Summary linked to this article.

## Online content

Any methods, additional references, Nature Portfolio reporting summaries, source data, extended data, supplementary information, acknowledgements, peer review information; details of author contributions and competing interests; and statements of data and code availability are available at 10.1038/s41557-025-01925-6.

## Supplementary information

Supplementary Information Materials, Supplementary Tables 1–3 and Figs. 1–22, synthetic procedures, NMR spectra, and R code used for processing of UV melting data.

Reporting Summary

## Source data

Source Data Fig. 3 Statistical source data.

Source Data Fig. 4 Unprocessed gels (Fig. 4b–e) and readme file providing context regarding no visible edges on gels in Fig. 4b–e.

Source Data Fig. 5 Statistical source data.

Source Data Fig. 6 Unprocessed gels (Fig. 6c,d).

Source Data Extended Data Fig. 1 Statistical source data.

Source Data Extended Data Fig. 2 Unprocessed gels for Extended Data Fig. 2a–d and f–h and readme file providing context regarding no visible edges on gels in panels a–d, g and h, and statistical source data for Extended Data Fig. 2e.

Source Data Extended Data Fig. 3 Statistical source data.

Source Data Extended Data Fig. 4 Unprocessed gels.

## Data Availability

Raw data for the oligonucleotide MS analysis (Fig. 5 and Extended Data Fig. 3) have been deposited in Apollo, the University of Cambridge Repository (10.17863/CAM.119812)^68^. Source data are provided with this paper.

## Code availability

The R script utilized to analyse the UV melting data is available within Supplementary Information. No further code was generated to support or analyse the findings of this study.
