# Supplementary material for: An unnatural base pair for the detection of epigenetic cytosine modifications in DNA
Source: Nat Chem. 2025 Aug 20;17(11):1732–41. doi: 10.1038/s41557-025-01925-6 (PMC12580329; doi:10.1038/s41557-025-01925-6)
Supplement: Supplementary file 1 — Materials, Supplementary Tables 1–3 and Figs. 1–22, synthetic procedures, NMR spectra, and R code used for processing of UV melting data. [file 41557_2025_1925_MOESM1_ESM.pdf]

nature chemistry

Supplementary information

https://doi.org/10.1038/s41557-025-01925-6

An unnatural base pair for the detection of epigenetic cytosine modifications in DNA

In the format provided by the authors and unedited

Table of Contents

Oligonucleotides and polymerases **2**

Steady-State Kinetics **4**

HPLC-HRMS/MS analysis **5**

- **Sequences of detected oligonucleotides** 5
- **Full scan MS spectra and MS^2-based sequence coverage** 5
- **Representative MS chromatograms for all templates** 12

Sanger sequencing experiments **16**

- **Concentration dependence of ddDTP misincorporation at natural template bases** 16
- **Polymerase pausing after incorporation of dATP opposite MfC** 17

Synthetic procedures **20**

- **General experimental details and instrumentation** 20
- **Synthesis of dD nucleoside** 21
- **Synthesis of dD phosphoramidite** 23
- **Synthesis of dDTP 2'-deoxynucleoside triphosphate** 25
- **Synthesis of ddDTP 2',3'-dideoxynucleoside triphosphate** 27

Supplementary references **32**

NMR spectra **33**

Supplementary Information Source Data **51**

R script for the processing of UV melting data **53**

Oligonucleotides and polymerases

Supplementary Table 1. Sequences of DNA oligonucleotides used in this study.

Experiment	Type (length)	#	Sequence (5'-to-3')	Additional info
dsDNA melting (UV-Vis)	forward (12 nt)	O1	GCG**X**TTTTTCGC	**X** = C, 5fC, T
	reverse (12 nt)		GCGAAAAA**Y**CGC	**Y** = D, A, G
	forward (12 nt)	O2	GCGTC**X**GTACGC	**X** = C, 5fC, T
	reverse (12 nt)		GCGTAC**Y**GACGC	**Y** = D, A, G
single nucleotide incorporation (denat. PAGE)	primer (25 nt)	P1	*FAM*–GCG AAT TAA CCC TCA CTA AAG TAC G	
	template (51 nt)	T1	GCG TAA TAC GAC TCA CTA TAG ACG A**X**C<br>GTA CTT TAG TGA GGG TTA ATT CGC	**X** = C, G, T, or 5fC
	template (51 nt)		GCG TAA TAC GAC TCA CTA TAG ACG TAC<br>GTA CTT TAG TGA GGG TTA ATT CGC	
Steady-state kinetics (denat. PAGE)	primer (15 nt)	P4	*FAM*–CCT CAC TAA AGT AGC	
	template (20 nt)	T7	CGT A**X**G CTA CTT TAG TGA GG	**X** = A, C, G, T, or 5fC
primer extension (denat. PAGE)	primer (20 nt)	P5	*FAM*–GCG AAT TAA CCC TCA GCT AC	
	template (26 nt)	T8	GAT CA**fC** GTA GCT GAG GGT TAA TTC GC	
incorporation competition (MS)	primer (15 nt)	P2	*FAM*–CCT CAC TAA AGT ACG	
	template (20 nt)	T2	CGT A**X**C GTA CTT TAG TGA GG	**X** = C, G, T, or 5fC
	template (20 nt)		CGT GAC GTA CTT TAG TGA GG	
Sanger sequencing (denat. PAGE)	primer (15 nt)	P3	*FAM*–GCG AAT TAA CCC TCA	
	template (51 nt)	T3	GCG TAA TAC GAC TCA CTA TAG ACG A**fC**C<br>GTA CTT TAG TGA GGG TTA ATT CGC	1 5fC
	template (51 nt)	T4	CGC TAA ACC GAT TCA **fC**TA TAG CAG<br>CAT **fC**GA CAT AGT TGA GGG TTA ATT CGC	2 5fC
	template (51 nt)	T5	GCG TAA TAC GAC TCA CTA TAG ACG ACC<br>GTA CTT TAG TGA GGG TTA ATT CGC	5fC-free control template
	template (51 nt)	T6	CGC TAA ACC GAT TCA CTA TAG CAG<br>CAT **fC**GA CAT AGT TGA GGG TTA ATT CGC	1 5fC
	template (51 nt)	T9	GCG TAA TAC GAC TCA CTA TAG ACT AG**fC**<br>GTA CTT TAG TGA GGG TTA ATT CGC	1 5fC
	template (51 nt)	T10	GCG TAA TAC GAC TCA CTA TAG A**fC**G ACC<br>GTA CTT TAG TGA GGG TTA ATT CGC	1 5fC

Supplementary Table 2. List of DNA Polymerases used in this study. Including recommended reaction buffer conditions.

Polymerase	Supplier	Reaction buffer recommended by supplier
KlenTaq	Jena Biosciences	20 mM Tris-HCl (pH 8.5), 40 mM KCl, 2.5 mM $MgCl_2$
Thermo Sequenase	Applied Biosystems/ Affymetrix	30 mM Tris-HCl (pH 9.5), 7.5 mM $MgCl_2$
Vent (exo–)	NEB	20 mM Tri-HCl (pH 8.8), 10 mM $(NH_4)_2SO_4$, 10 mM KCl, 2 mM $MgSO_4$
Deep Vent (exo–)	NEB	20 mM Tri-HCl (pH 8.8), 10 mM $(NH_4)_2SO_4$, 10 mM KCl, 2 mM $MgSO_4$
Bst LF	NEB	20 mM Tri-HCl (pH 8.8), 10 mM $(NH_4)_2SO_4$, 10 mM KCl, 2 mM $MgSO_4$
KF (exo–)	NEB	10 mM Tris-HCl (pH 7.9), 50 mM NaCl, 10 mM $MgCl_2$, 1 mM DTT
CycleSeq Pol	Applied Biosystems/ Affymetrix	25 mM TAPS (pH 9.3), 50 mM KCl, 2 mM MgCl2, 1 mM β-ME
E. coli Pol I KF	NEB	10 mM Tris-HCl (pH 7.9), 50 mM NaCl, 10 mM $MgCl_2$, 1 mM DTT
Bsu LF	NEB	10 mM Tris-HCl (pH 7.9), 50 mM NaCl, 10 mM $MgCl_2$, 1 mM DTT
Taq	NEB	10 mM Tris-HCl (pH 8.3), 50 mM KCl, 1.5 mM $MgCl_2$
Vent	NEB	20 mM Tri-HCl (pH 8.8), 10 mM $(NH_4)_2SO_4$, 10 mM KCl, 2 mM $MgSO_4$
Deep Vent	NEB	20 mM Tri-HCl (pH 8.8), 10 mM $(NH_4)_2SO_4$, 10 mM KCl, 2 mM $MgSO_4$
Therminator	NEB	20 mM Tri-HCl (pH 8.8), 10 mM $(NH_4)_2SO_4$, 10 mM KCl, 2 mM $MgSO_4$

Steady-State Kinetics

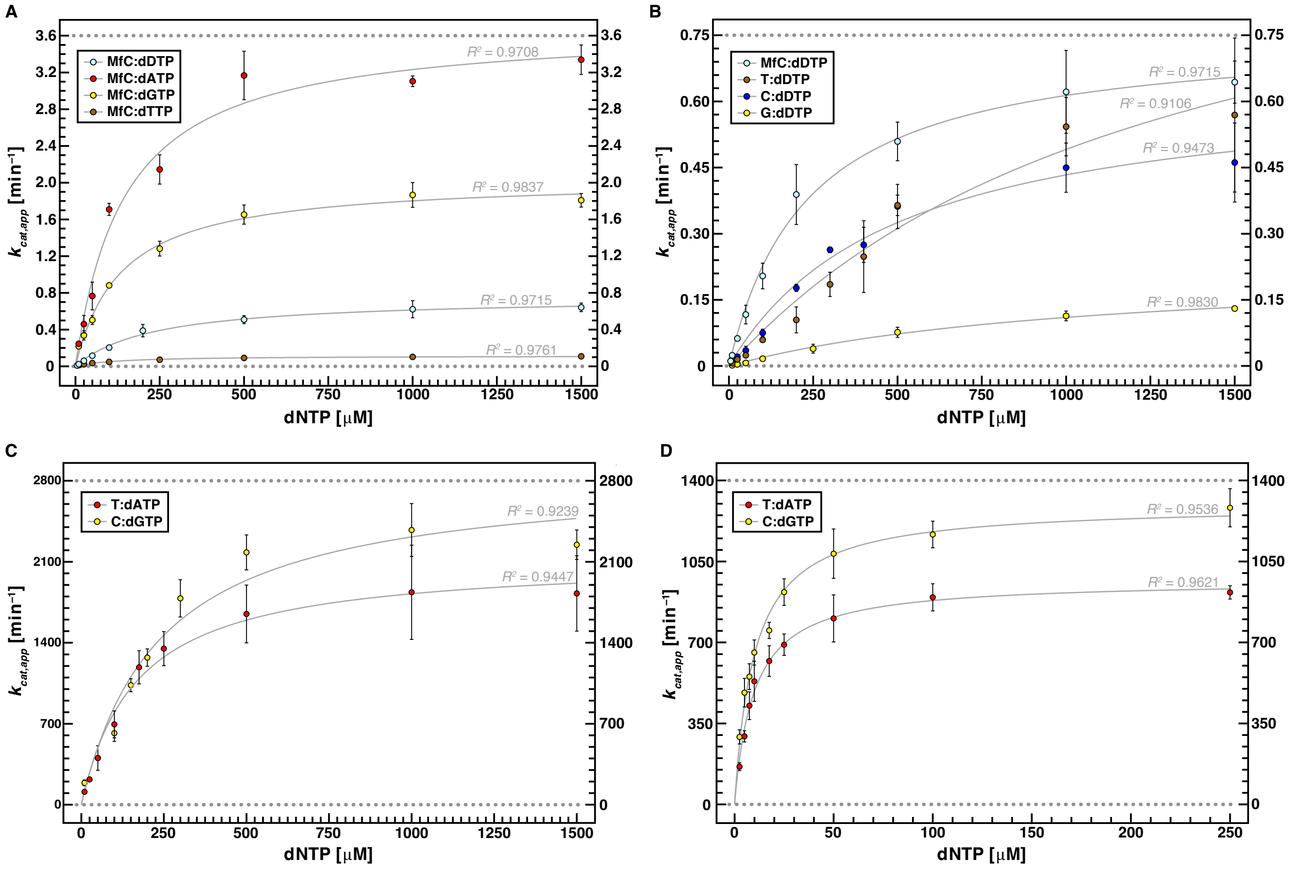

Supplementary Fig. 1|Steady-state kinetic analysis of KlenTaq DNA polymerase at pH 7.0. Examination of dNTP incorporation opposite **MfC**, as well as **dDTP** incorporation opposite various template bases. Means of three independent experiments are shown with standard deviations (n = 3). The corresponding k_{cat} and K_M values are listed in Extended Data Fig. 2**E**. **A**: Determination of apparent rate constants ($k_{cat,app}$) for incorporation of different dNTP substrates opposite **MfC**, including a non-linear fit to the Michaelis-Menten equation (grey) to obtain the corresponding K_M and k_{cat} values. **B**: Determination of apparent rate constants ($k_{cat,app}$) for incorporation of **dDTP** substrates opposite various template bases, including a non-linear fit to the Michaelis-Menten equation (grey) to obtain the corresponding K_M and k_{cat} values. **C**: Determination of apparent rate constants ($k_{cat,app}$) for incorporation of dATP opposite T and dGTP opposite C, including a non-linear fit to the Michaelis-Menten equation (grey) to obtain the corresponding K_M and k_{cat} values. **D**: Determination of apparent rate constants ($k_{cat,app}$) for incorporation of dATP opposite T and dGTP opposite C at pH 8.5, including a non-linear fit to the Michaelis-Menten equation (grey) to obtain the corresponding K_M and k_{cat} values.

HPLC-HRMS/MS analysis

This section contains details of the MS analysis for the single nucleotide incorporation competition experiments conducted with Thermo Sequenase. The 15-mer, 16-mer and 17-mer oligonucleotides observed in those experiments, and their sequences, are listed in Supplementary Table 3. The corresponding full scan spectra and the MS^2-based sequence coverage maps for each oligonucleotide are shown in Supplementary Fig. 2–13. We further provide chromatograms (r-BPC and SIC for each compound present) of representative reaction mixtures in Supplementary Fig. 14–18, to illustrate product separation and distribution. Using the chosen HPLC-HRMS/MS method, the BioPharmaFinder^{TM} software captured oligonucleotides down to 0.2% of total DNA molecules in a sample (ca 5 fmol).

Sequences of detected oligonucleotides

Supplementary Table 3. Sequences of primer and product oligonucleotides identified and analysed in HPLC-ESI$^-$-HRMS/MS experiments. Respective full scan mass spectra and MS^2-based sequence coverage are shown in Supplementary Fig. 1–12.

Description	Sequence	Description	Sequence
primer	*FAM*–CCT CAC TAA AGT ACG	primer+A+D	*FAM*–CCT CAC TAA AGT ACG AD
primer+D	*FAM*–CCT CAC TAA AGT ACG D	primer+G+D	*FAM*–CCT CAC TAA AGT ACG GD
primer+A	*FAM*–CCT CAC TAA AGT ACG A	primer+C+D	*FAM*–CCT CAC TAA AGT ACG CD
primer+G	*FAM*–CCT CAC TAA AGT ACG G	primer+T+D	*FAM*–CCT CAC TAA AGT ACG TD
primer+C	*FAM*–CCT CAC TAA AGT ACG C	primer+A+A	*FAM*–CCT CAC TAA AGT ACG AA
primer+T	*FAM*–CCT CAC TAA AGT ACG T	primer+G+A	*FAM*–CCT CAC TAA AGT ACG GA

Full scan MS spectra and MS^2-based sequence coverage

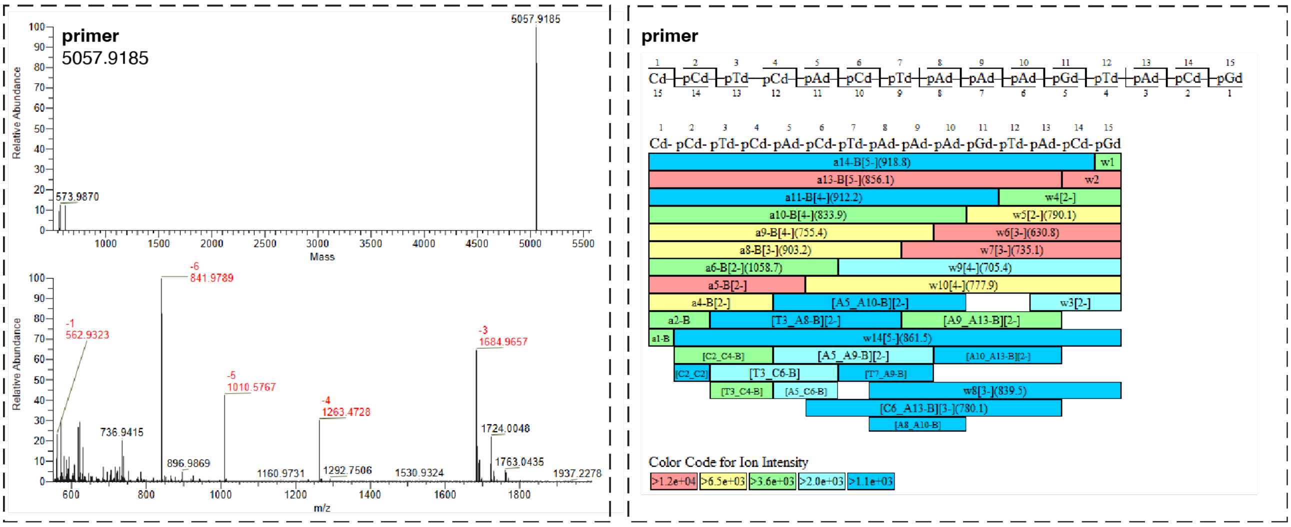

Supplementary Fig. 2 | MS-based identification of the 15-mer primer oligonucleotide (see Supplementary Table 3). Left: Full scan MS spectra (top: deconvoluted spectrum; bottom: charge state-resolved spectrum). **Right:** Sequence coverage based on the fragments found in the MS^2 scan. Results of a single experiment (n = 1, not repeated but representative for all HPLC-MS/MS experiments) are shown.

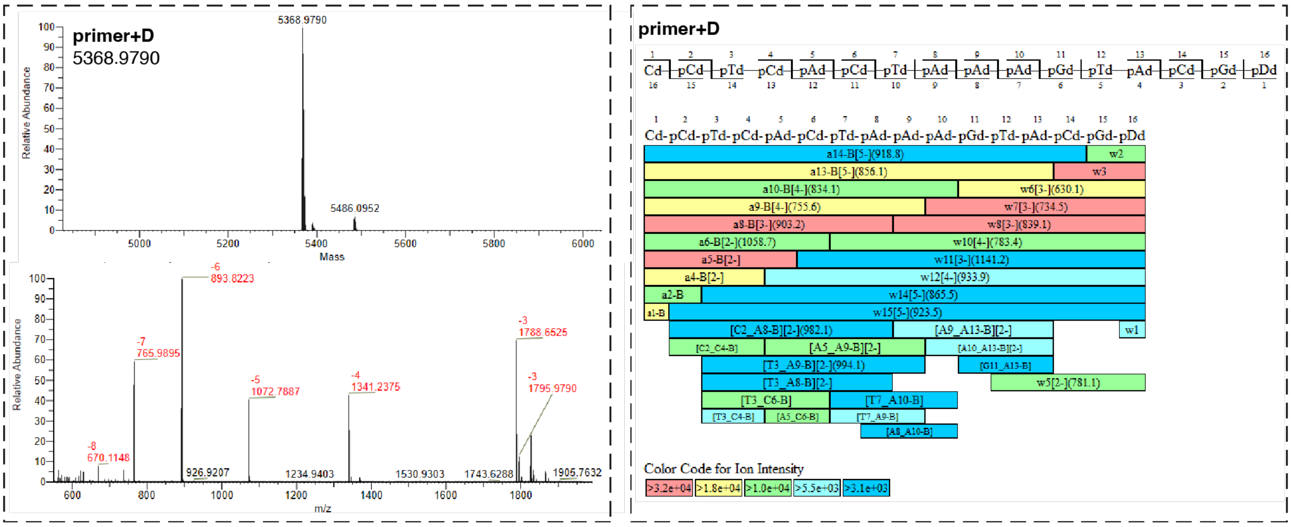

Supplementary Fig. 3 | MS-based identification of the 16-mer primer+D oligonucleotide (see Supplementary Table 3). Left: Full scan MS spectra (top: deconvoluted spectrum; bottom: charge state-resolved spectrum). **Right:** Sequence coverage based on the fragments found in the MS^2 scan. Results of a single experiment (n = 1, not repeated but representative for all HPLC-MS/MS experiments) are shown.

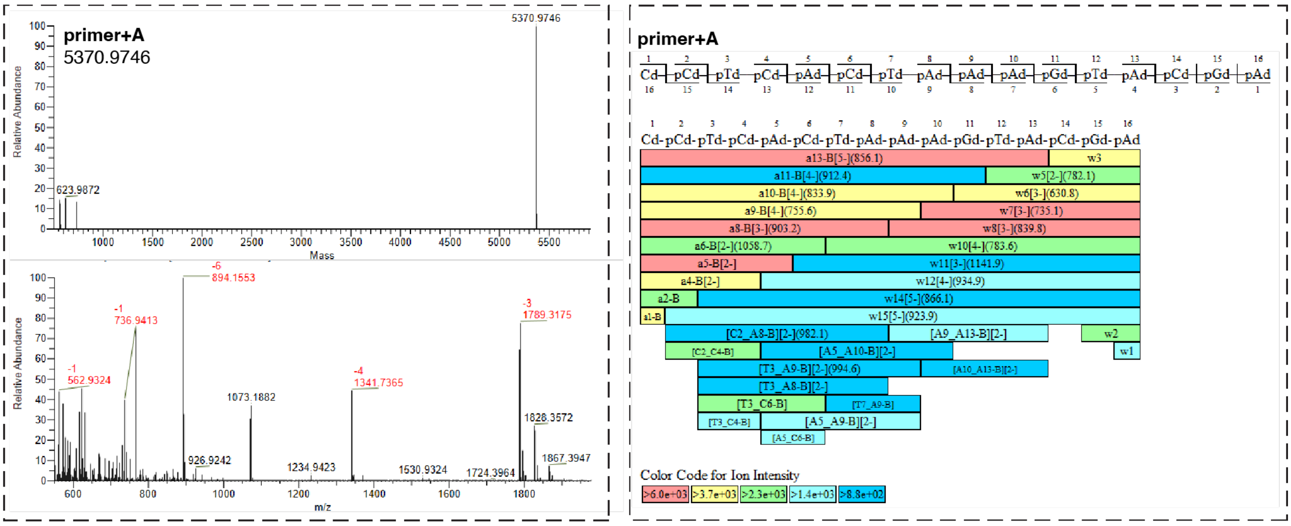

Supplementary Fig. 4 | MS-based identification of the 16-mer primer+A oligonucleotide (see Supplementary Table 3). Left: Full scan MS spectra (top: deconvoluted spectrum; bottom: charge state-resolved spectrum). **Right:** Sequence coverage based on the fragments found in the MS^2 scan. Results of a single experiment (n = 1, not repeated but representative for all HPLC-MS/MS experiments) are shown.

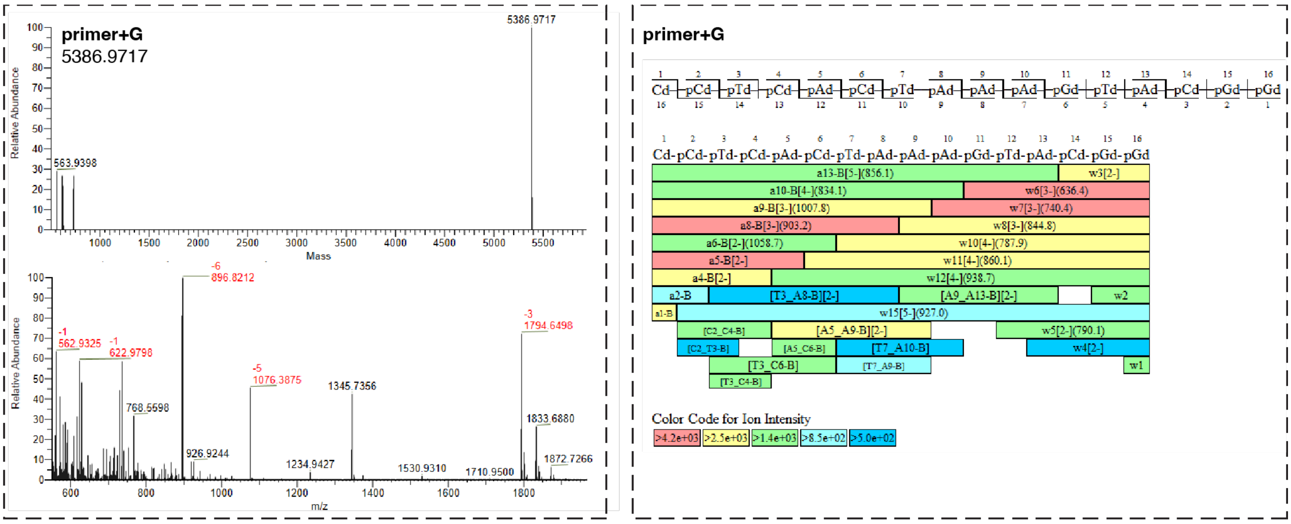

Supplementary Fig. 5 | MS-based identification of the 16-mer primer+G oligonucleotide (see Supplementary Table 3). Left: Full scan MS spectra (top: deconvoluted spectrum; bottom: charge state-resolved spectrum). **Right:** Sequence coverage based on the fragments found in the MS² scan. Results of a single experiment (n = 1, not repeated but representative for all HPLC-MS/MS experiments) are shown.

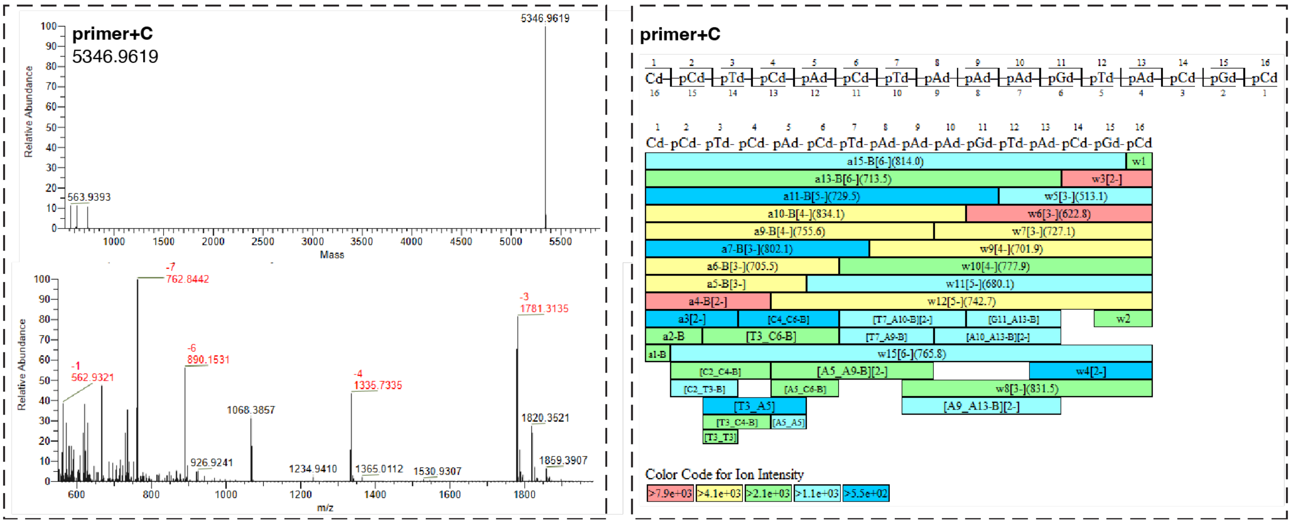

Supplementary Fig. 6 | MS-based identification of the 16-mer primer+C oligonucleotide (see Supplementary Table 3). Left: Full scan MS spectra (top: deconvoluted spectrum; bottom: charge state-resolved spectrum). **Right:** Sequence coverage based on the fragments found in the MS² scan. Results of a single experiment (n = 1, not repeated but representative for all HPLC-MS/MS experiments) are shown.

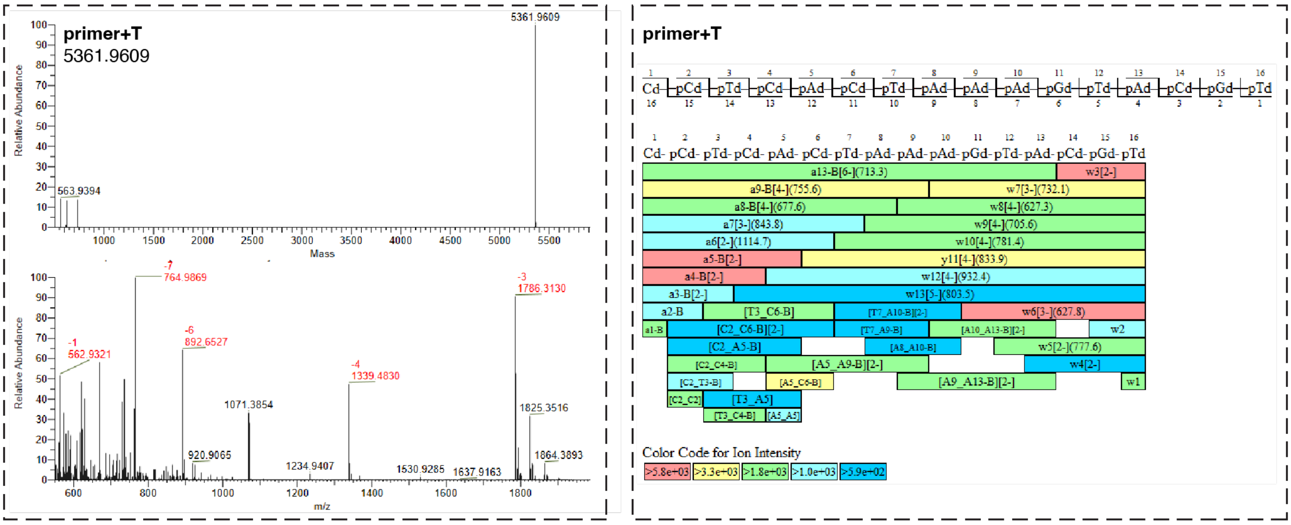

Supplementary Fig. 7 | MS-based identification of the 16-mer primer+T oligonucleotide (see Supplementary Table 3). Left: Full scan MS spectra (top: deconvoluted spectrum; bottom: charge state-resolved spectrum). **Right:** Sequence coverage based on the fragments found in the MS^2 scan. Results of a single experiment (n = 1, not repeated but representative for all HPLC-MS/MS experiments) are shown.

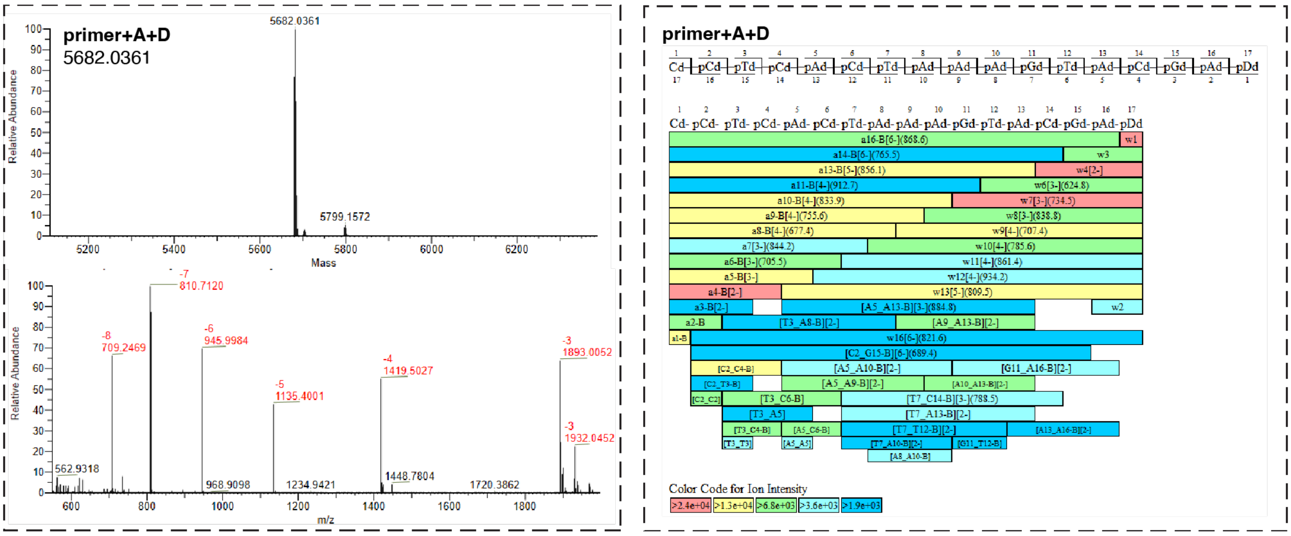

Supplementary Fig. 8 | MS-based identification of the 17-mer primer+A+D oligonucleotide (see Supplementary Table 3). Left: Full scan MS spectra (top: deconvoluted spectrum; bottom: charge state-resolved spectrum). **Right:** Sequence coverage based on the fragments found in the MS^2 scan. Results of a single experiment (n = 1, not repeated but representative for all HPLC-MS/MS experiments) are shown.

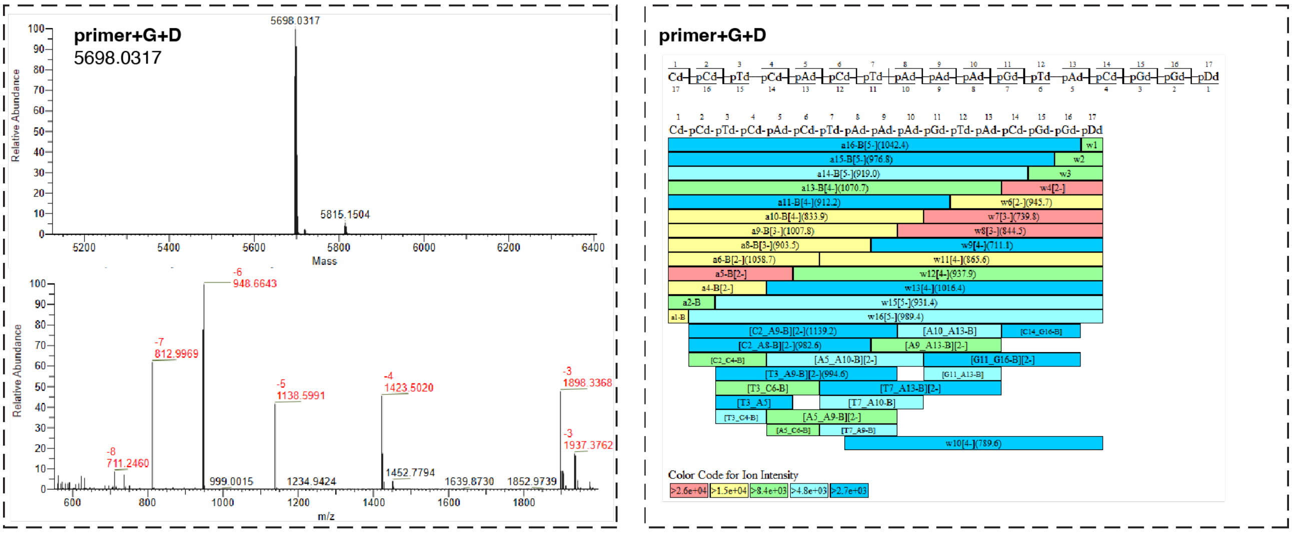

Supplementary Fig. 9 | MS-based identification of the 17-mer primer+G+D oligonucleotide (see Supplementary Table 3). Left: Full scan MS spectra (top: deconvoluted spectrum; bottom: charge state-resolved spectrum). **Right:** Sequence coverage based on the fragments found in the MS² scan. Results of a single experiment (n = 1, not repeated but representative for all HPLC-MS/MS experiments) are shown.

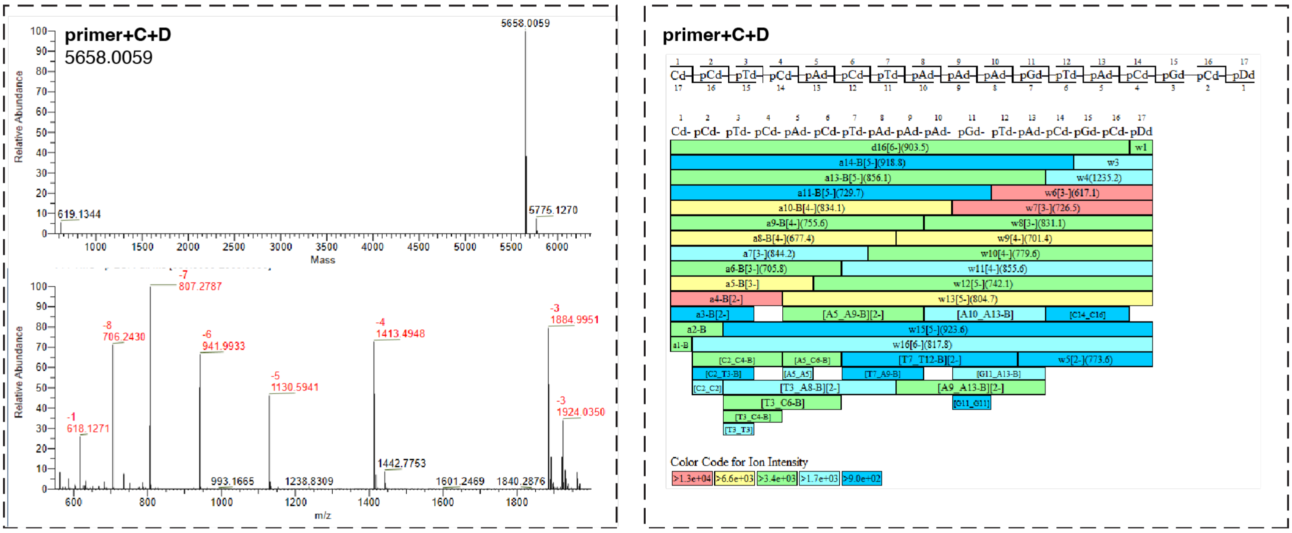

Supplementary Fig. 10 | MS-based identification of the 17-mer primer+C+D oligonucleotide (see Supplementary Table 3). Left: Full scan MS spectra (top: deconvoluted spectrum; bottom: charge state-resolved spectrum). **Right:** Sequence coverage based on the fragments found in the MS² scan. Results of a single experiment (n = 1, not repeated but representative for all HPLC-MS/MS experiments) are shown.

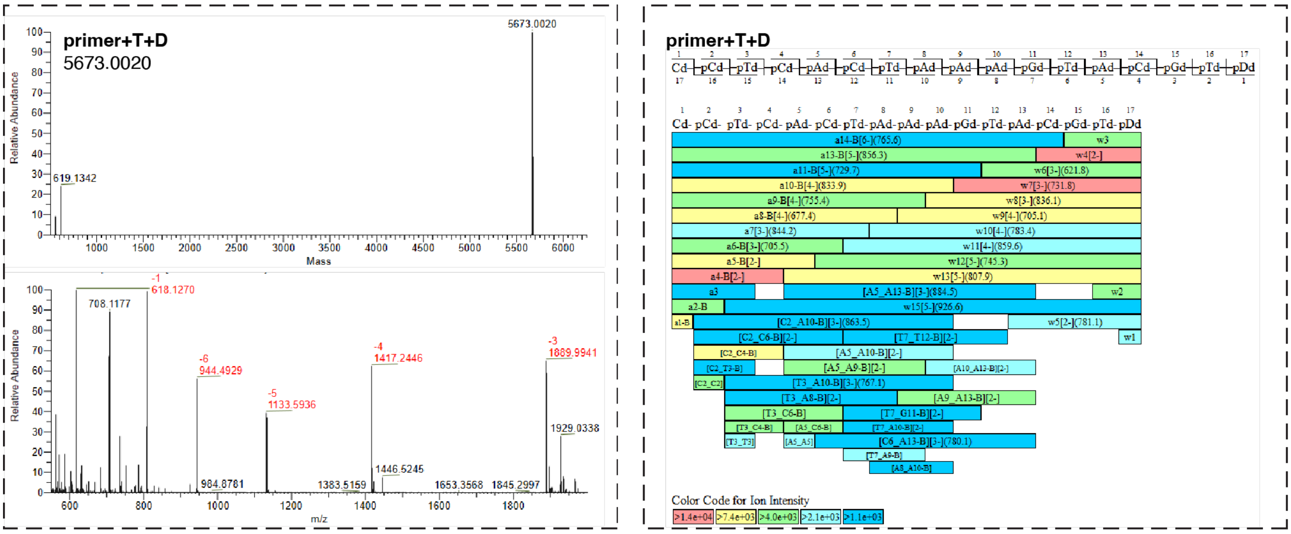

Supplementary Fig. 11|MS-based identification of the 17-mer primer+T+D oligonucleotide (see Supplementary Table 3). Left: Full scan MS spectra (top: deconvoluted spectrum; bottom: charge state-resolved spectrum). **Right:** Sequence coverage based on the fragments found in the MS² scan. Results of a single experiment (n = 1, not repeated but representative for all HPLC-MS/MS experiments) are shown.

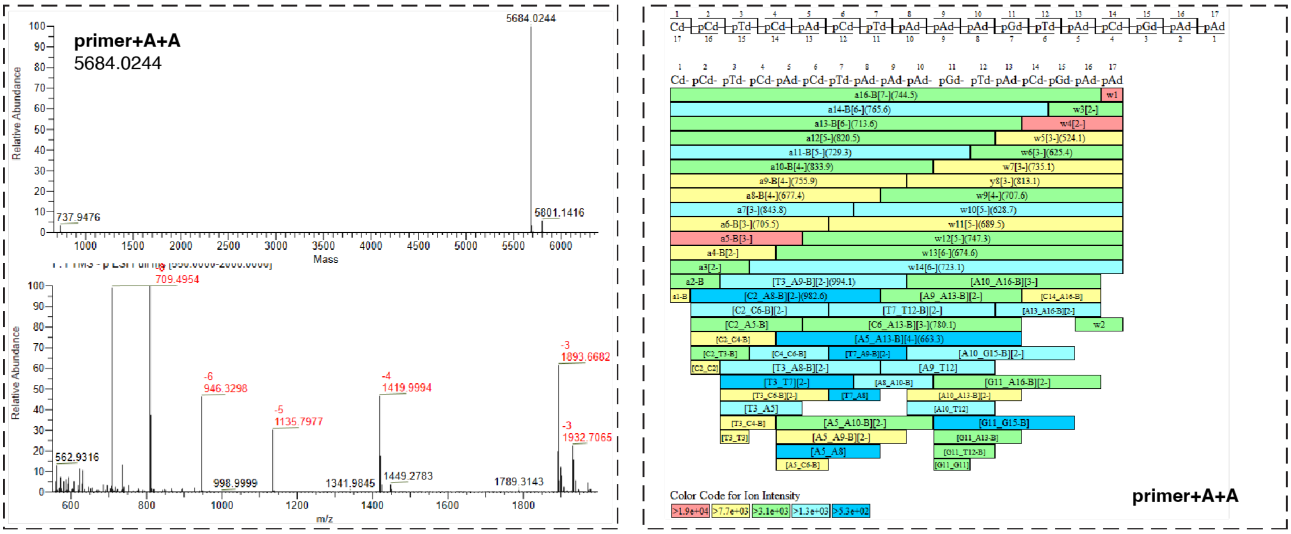

Supplementary Fig. 12|MS-based identification of the 17-mer primer+A+A oligonucleotide (see Supplementary Table 3). Left: Full scan MS spectra (top: deconvoluted spectrum; bottom: charge state-resolved spectrum). **Right:** Sequence coverage based on the fragments found in the MS² scan. Results of a single experiment (n = 1, not repeated but representative for all HPLC-MS/MS experiments) are shown.

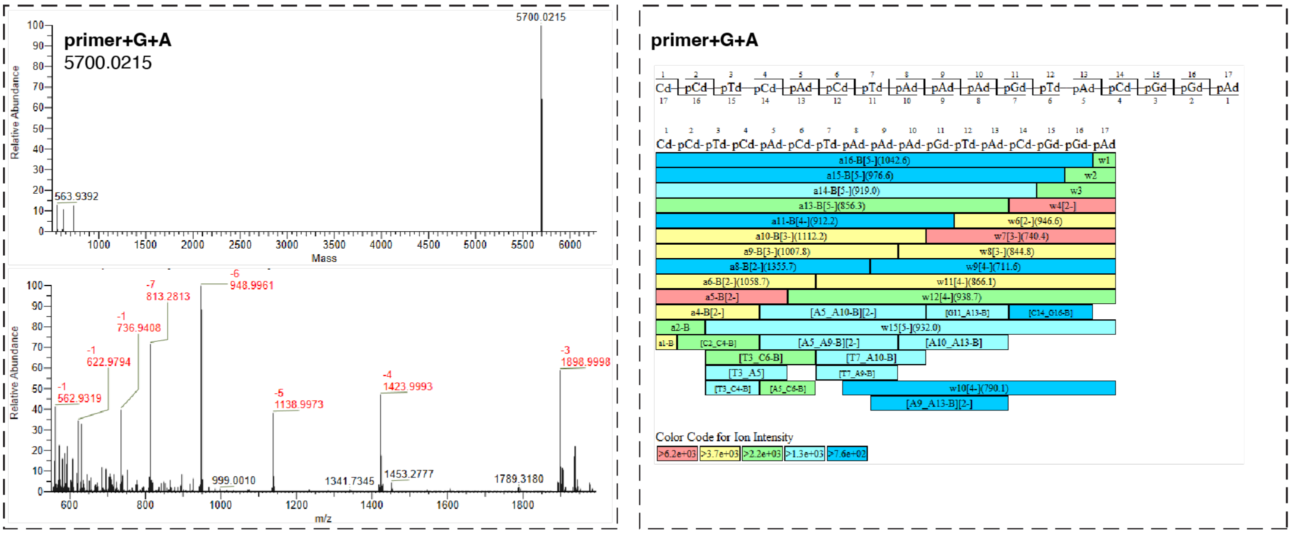

Supplementary Fig. 13|MS-based identification of the 17-mer primer+G+A oligonucleotide (see Supplementary Table 3). Left: Full scan MS spectra (top: deconvoluted spectrum; bottom: charge state-resolved spectrum). **Right:** Sequence coverage based on the fragments found in the MS^2 scan. Results of a single experiment (n = 1, not repeated but representative for all HPLC-MS/MS experiments) are shown.

Representative MS chromatograms for all templates

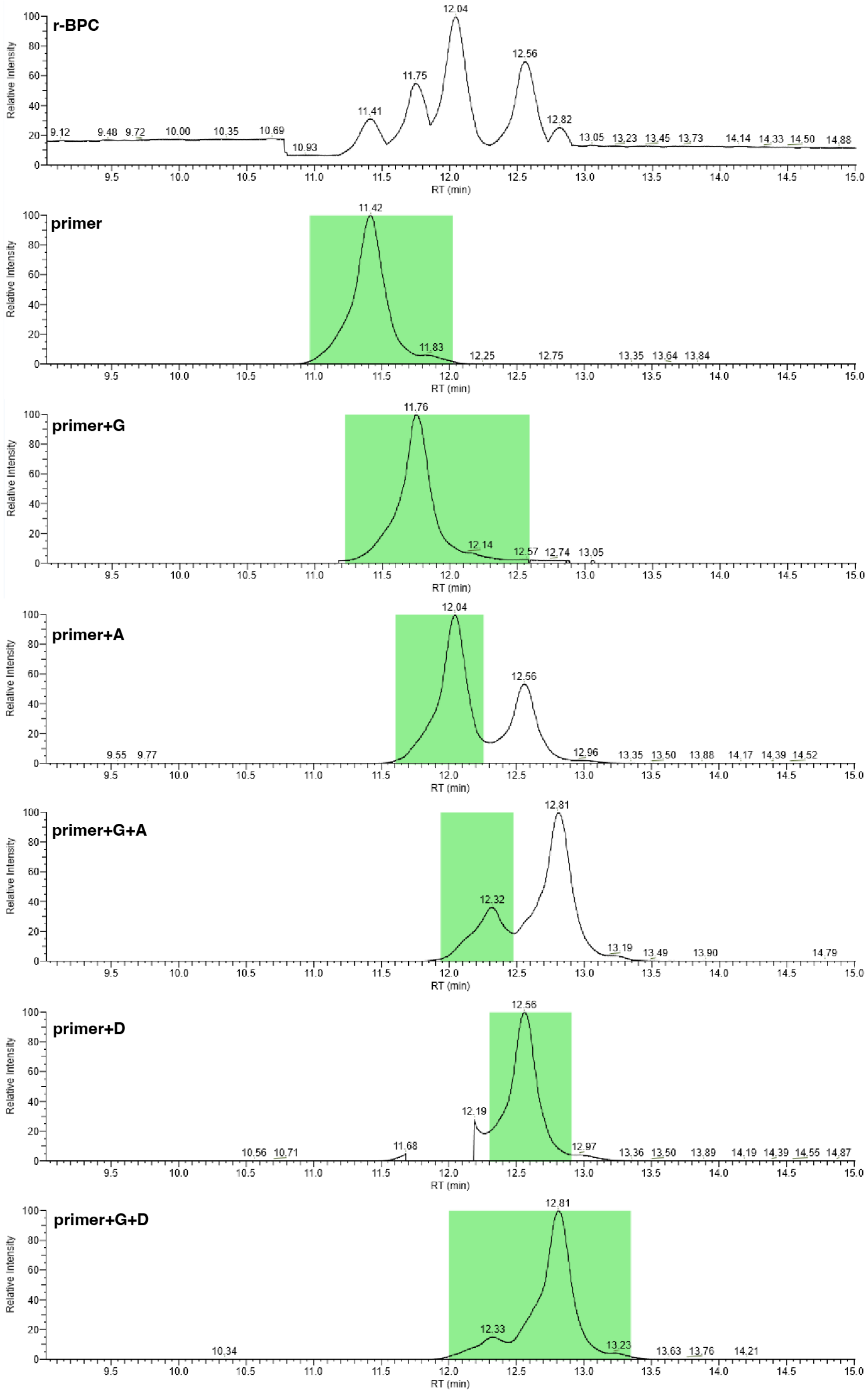

Supplementary Fig. 14 | Chromatogram of a representative competition reaction between dATP, dGTP and dDTP (1 equivalent) for incorporation opposite MfC at pH 7.0. Top: reduced BPC, showing all the observed component peaks. **Below**: SICs of the individual components detected (sequences in Supplementary Table 3). Results of a single experiment (n = 1, not repeated but representative for all HPLC-MS/MS experiments) are shown.

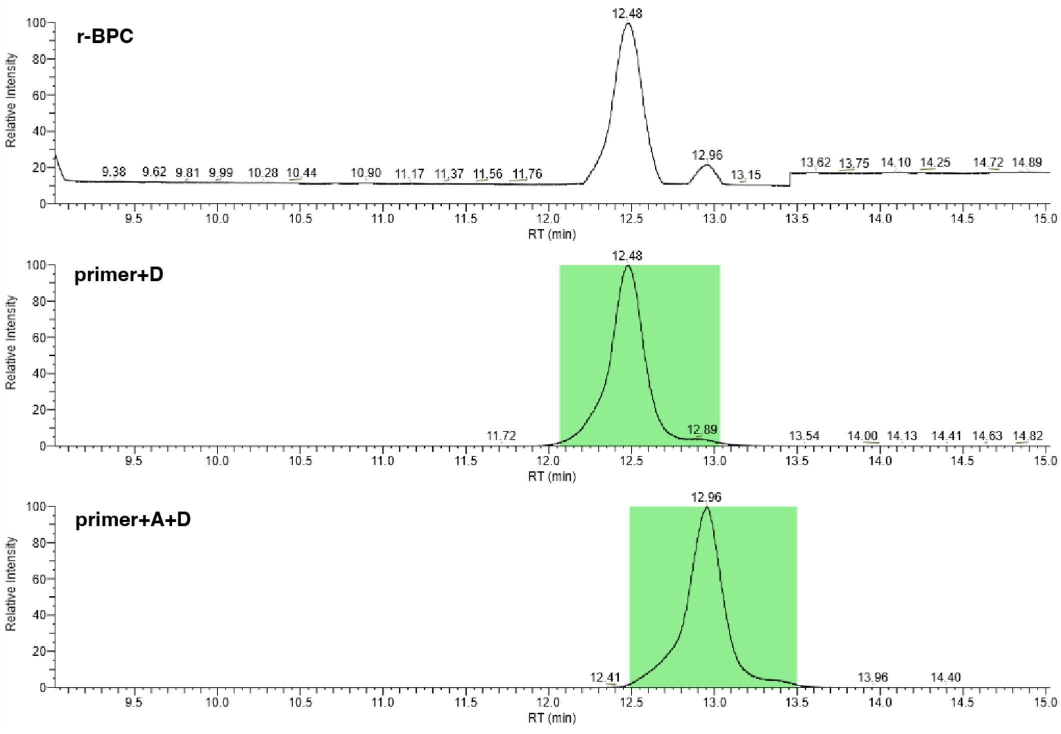

Supplementary Fig. 15|Chromatogram of a representative competition reaction between dATP and dDTP (100 equivalents) for incorporation opposite T at pH 9.5. Top: reduced BPC, showing all the observed component peaks. **Below**: SICs of the individual components detected (sequences in Supplementary Table 3). Results of a single experiment (n = 1, not repeated but representative for all HPLC-MS/MS experiments) are shown.

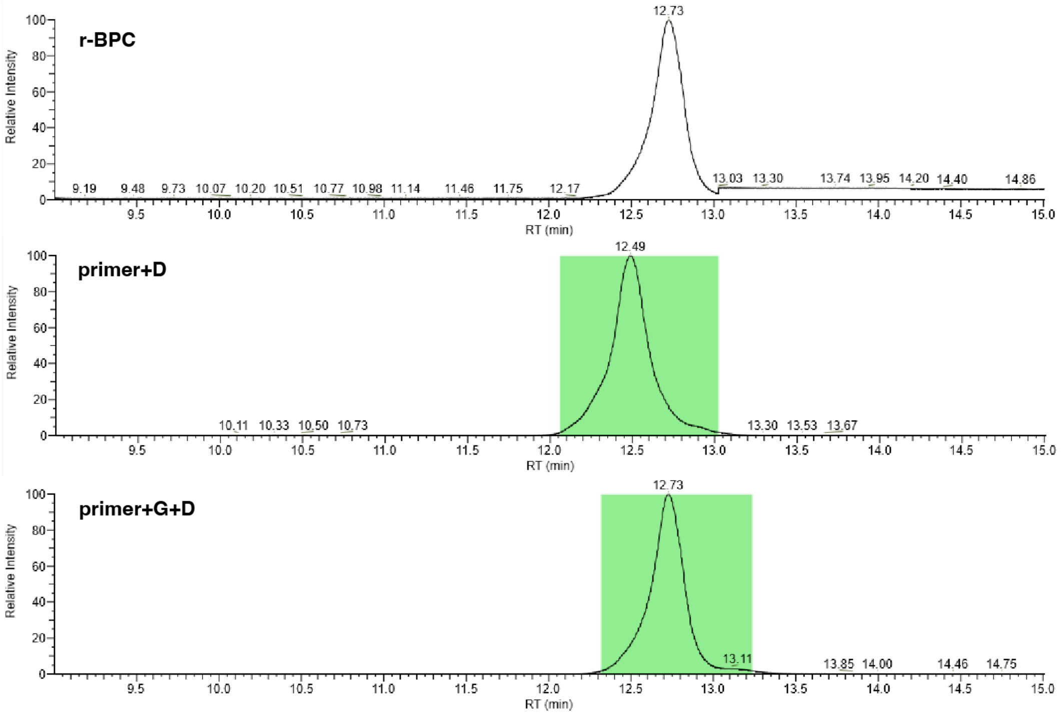

Supplementary Fig. 16|Chromatogram of a representative competition reaction between dGTP and dDTP (100 equivalents) for incorporation opposite C at pH 9.5. Top: reduced BPC, showing all the observed component peaks. **Below**: SICs of the individual components detected (sequences in Supplementary Table 3). Results of a single experiment (n = 1, not repeated but representative for all HPLC-MS/MS experiments) are shown.

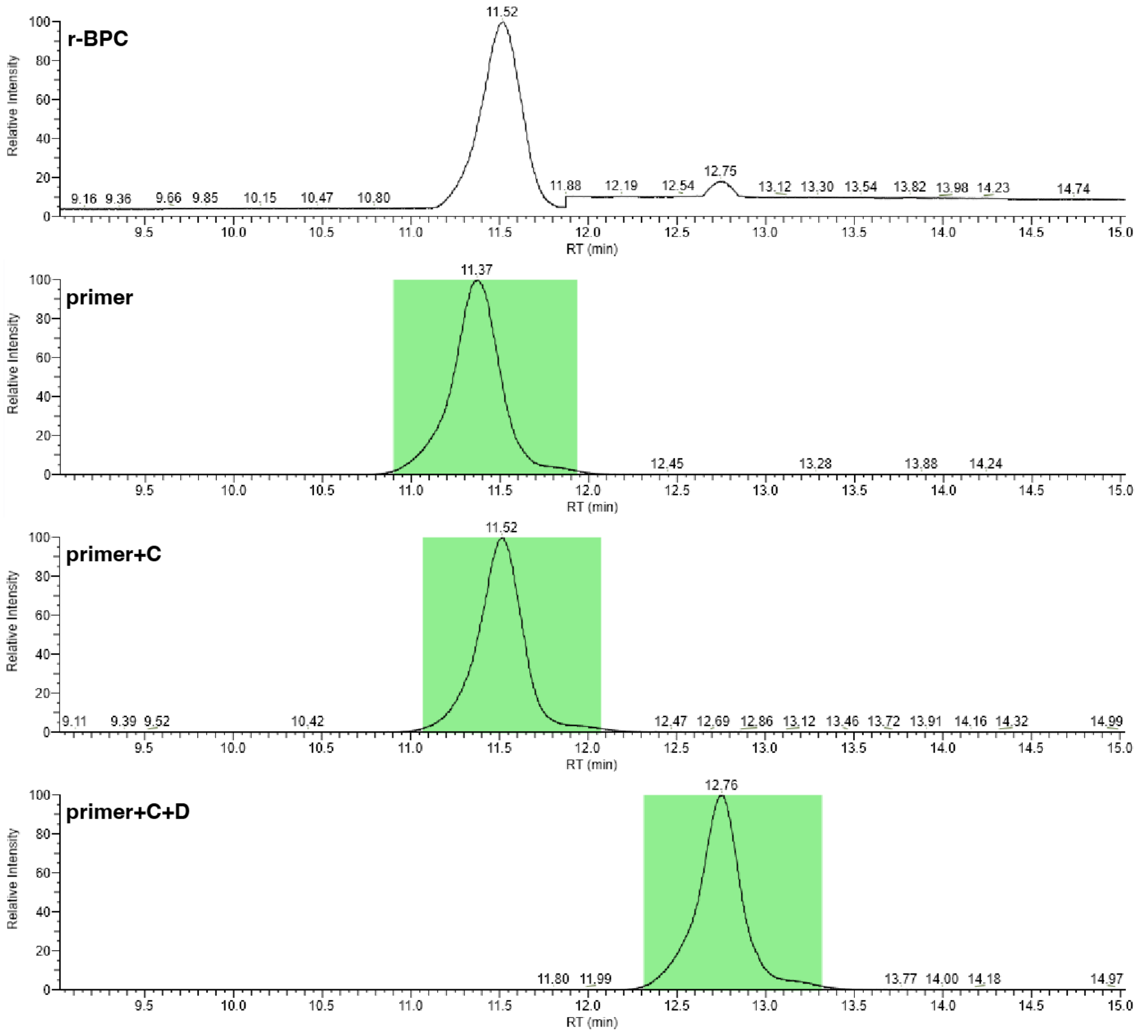

Supplementary Fig. 17|Chromatogram of a representative competition reaction between dCTP and dDTP (100 equivalents) for incorporation opposite G at pH 7.0. Top: reduced BPC, showing all the observed component peaks. **Below**: SICs of the individual components detected (sequences in Supplementary Table 3). Results of a single experiment (n = 1, not repeated but representative for all HPLC-MS/MS experiments) are shown.

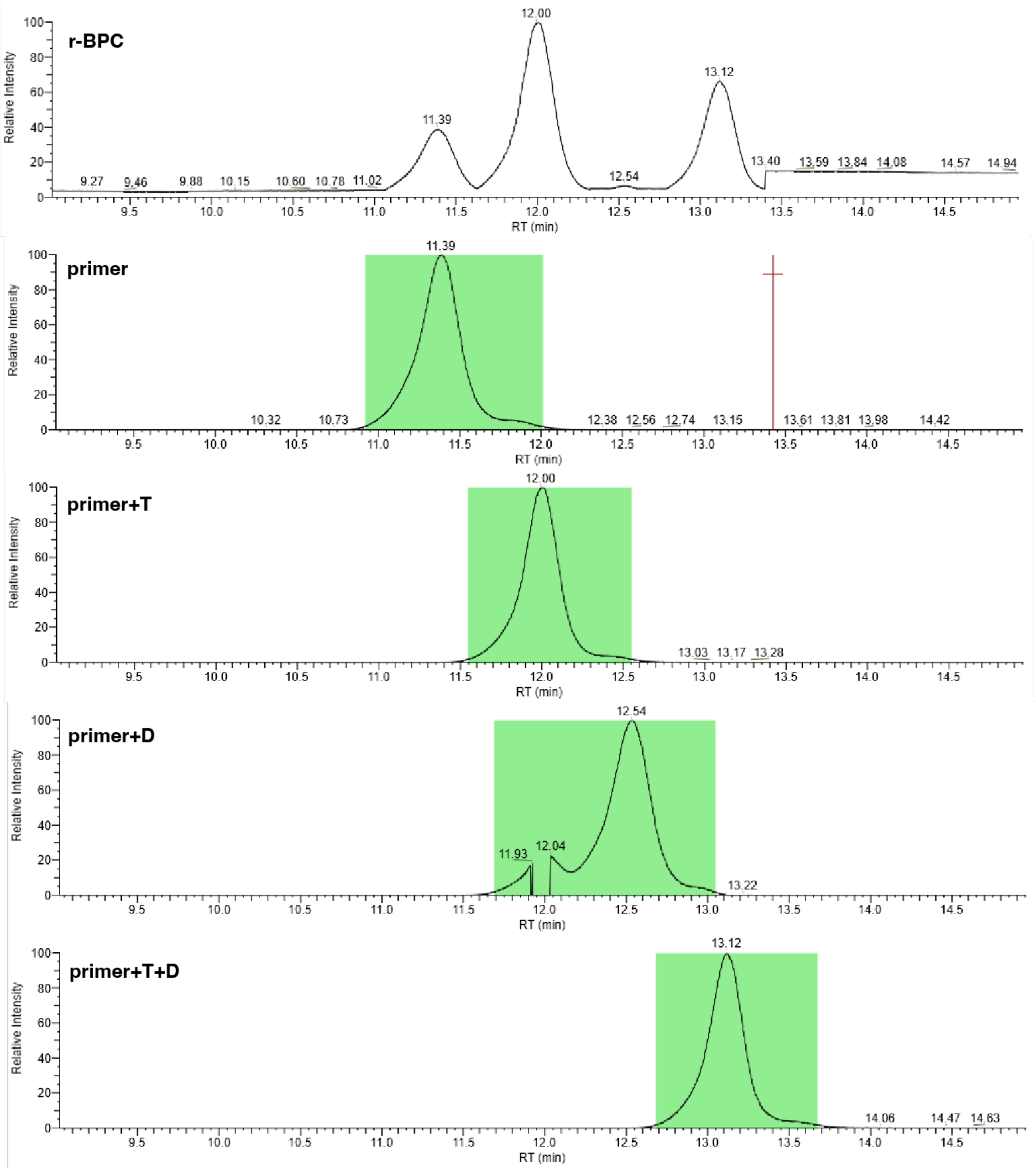

Supplementary Fig. 18|Chromatogram of a representative competition reaction between dTTP and dDTP (100 equivalents) for incorporation opposite A at pH 7.0. Top: reduced BPC, showing all the observed component peaks. **Below**: SICs of the individual components detected (sequences in Supplementary Table 3). Results of a single experiment (n = 1, not repeated but representative for all HPLC-MS/MS experiments) are shown.

Sanger sequencing experiments

This section contains additional data about the optimisation of the Sanger sequencing experiments. This includes experiments to determine a viable **ddDTP** concentration (Supplementary Fig. 19), and to rationalise the concentrations of dATP and dGTP in the dNTP mix to reduce background termination around the **MfC** base (Supplementary Fig. 20–22).

Concentration dependence of ddDTP misincorporation at natural template bases

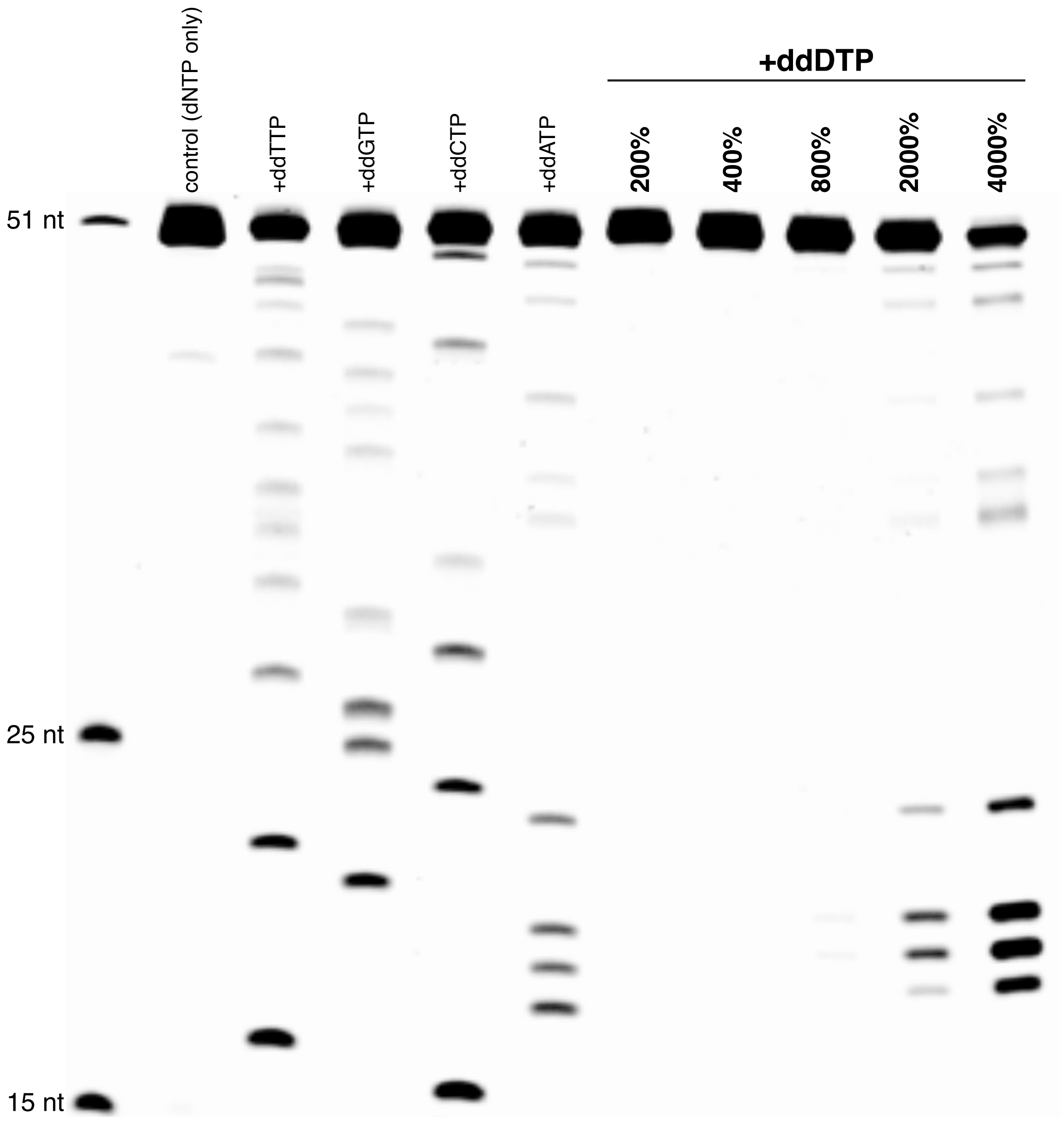

Supplementary Fig. 19 | Sanger sequencing gel to investigate ddDTP misincorporation depending on its concentration with a MfC-free template (T5; see Supplementary Table 1). No **ddDTP** termination bands are observed up to 800% **ddDTP** (relative to dATP); above 800%, termination at T occurs visibly. Conditions: 2 U Thermo Sequenase, pH 7.0; 37.5 µM dATP, 75 µM dCTP, 150 µM dGTP, 75 µM dTTP; 3% ddATP, 3% ddCTP, 3% ddGTP, 4% ddTTP (relative to corresponding dNTP); **ddDTP** concentration between 75–1500 µM (200%–4000% relative to dATP); 60°C, 2 h. Results of a single experiment (n = 1, not repeated) are shown.

Polymerase pausing after incorporation of dATP opposite MfC

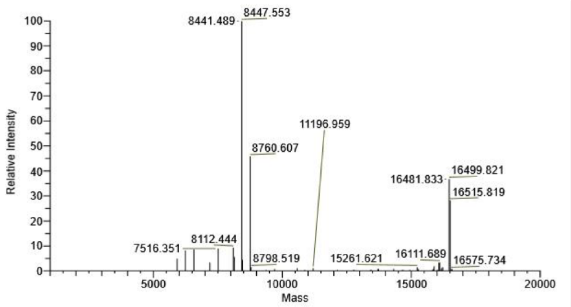

Sequence of 26mer ddDTP termination product:

FAM–GCGAATTAACCCTCACTAAAGTACG**D(3'-deoxy)**

Sequence of 27mer ddDTP termination product:

FAM–GCGAATTAACCCTCACTAAAGTACG**AD(3'-deoxy)**

Results

		Level	Sequence Name	Modification	Monoisotopic Mass	Theoretical Mass (Da)	Matched Mass Error (ppm)	Average Mass	Sum Intensity	Relative Abundance	Fractional Abundance	Number of Charge States	Charge State Distribution	Number of Detected Intervals	Delta Mass	Scan Range	Start Time (min)	Stop Time (min)	Apex RT
11	☑	Component	**26-mer pdt: 25mer+ddD**		8447.553	0.000	0.0	8,451.50	2.17E+06	100.00	25.66	8	5 - 12	6	0.000	7833 - 8001	21.952	22.399	22.15
12	☑	Component	26-mer pdt: 25mer+dA		8465.499	0.000	0.0	8,469.45	2.70E+04	1.24	0.32	3	7 - 9	4	17.946	7426 - 7549	20.799	21.149	20.95
13	☑	Component	26-mer pdt: 25mer+ddD+Na		8469.494	0.000	0.0	8,473.45	1.03E+05	4.76	1.22	6	5 - 10	5	21.940	7833 - 7982	21.952	22.350	22.15
14	☐	Component	26-mer pdt: 25mer+dG		8481.464	0.000	0.0	8,485.42	1.66E+04	0.77	0.20	3	7 - 9	3	33.911	7356 - 7478	20.601	20.950	20.8
15	☑	Component	26-mer pdt: 25mer+ddD+K		8485.465	0.000	0.0	8,489.43	9.75E+04	4.49	1.15	3	7 - 9	5	37.911	7833 - 7982	21.952	22.350	22.15
16	☑	Component	**27-mer pdt: 25mer+dA+ddD**		8760.607	0.000	0.0	8,764.69	1.00E+06	46.05	11.82	8	5 - 12	7	313.054	7945 - 8131	22.250	22.750	22.5
17	☑	Component	27-mer pdt: 25mer+dA+dA		8778.552	0.000	0.0	8,782.65	8.16E+04	3.76	0.96	7	5 - 11	6	330.999	7497 - 7655	21.001	21.449	21.25
18	☐	Component	27-mer pdt: 25mer+dA+ddD+Na		8782.552	0.000	0.0	8,786.65	3.91E+04	1.80	0.46	6	5 - 11	4	334.998	7964 - 8095	22.299	22.649	22.5
19	☑	Component			8794.551	0.000	0.0	8,798.65	3.42E+04	1.58	0.40	3	7 - 9	5	346.998	7444 - 7584	20.850	21.250	21.05

Supplementary Fig. 20|MS analysis of ddDTP termination products with 51-mer Sanger sequencing template T3 (Supplementary Table 1) which contains MfC at position 26. The main components (in red) are the desired 26-mer **ddDTP** termination product and a 27-mer formed by incorporation of dATP opposite **MfC** upon which the polymerase stalled and incorporated **ddDTP** before dissociating. Fragments containing dGTP are only found in traces. Assay conducted with high dGTP/low dATP conditions as shown in Supplementary Fig. 22 lanes 5+6 (150 μM dGTP, 37.5 μM dATP, 112.5 μM **ddDTP** (300% relative to dATP)). MS analysis with the Intact Deconvolution Tool of the BioPharma Finder™ software (version 4.1). **Top:** deconvoluted spectrum showing masses of all fragments found (masses in the 16 kDa region correspond to fully elongated 51-mer or 52-mer oligonucleotides). **Bottom:** details on the fragments found around 8.4–8.8 kDa which corresponds to 26-mer or 27-mer oligonucleotides). Main components in red. Results of a single experiment (n = 1, not repeated) are shown.

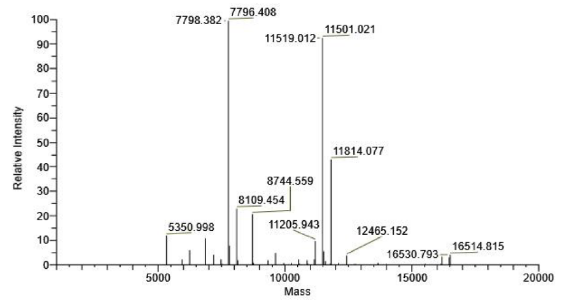

Sequence of 24mer ddDTP termination product:

FAM–GCGAATTAACCCTCAACTATGTC**D(3'-deoxy)**

Sequence of 25mer ddDTP termination product:

FAM–GCGAATTAACCCTCAACTATGTC**AD(3'-deoxy)**

Sequence of 36mer ddDTP termination product:

FAM–GCGAATTAACCCTCAACTATGTC**[A/G]**ATGCTGCTATA**D(3'-deoxy)**

Sequence of 37mer ddDTP termination product:

FAM–GCGAATTAACCCTCAACTATGTC**[A/G]**ATGCTGCTATA**AD(3'-deoxy)**

Results

#	☐	Level	Sequence Name	Modification	Monoisotopic Mass	Theoretical Mass (Da)	Matched Mass Error (ppm)	Average Mass	Sum Intensity	Relative Abundance	Fractional Abundance	Number of Charge States	Charge State Distribution	Number of Detected Intervals	Delta Mass	Scan Range	Start Time (min)	Stop Time (min)	Apex RT
9	✓	Component	**24-mer pdt: 23mer+ddD**		7796.408	0.000	0.0	7,800.05	1.93E+06	100.00	20.57	8	4 - 11	7	0.000	7734 - 7913	21.700	22.201	21.9
10		Component			7798.382	0.000	0.0	7,802.03	3.55E+04	1.84	0.38	5	4 - 9	5	1.975	7399 - 7539	20.752	21.150	20.95
11	✓	Component	24-mer pdt: 23mer+ddD+Na		7818.372	0.000	0.0	7,822.03	1.55E+05	8.07	1.66	7	4 - 10	6	21.965	7734 - 7895	21.700	22.150	21.9
12		Component	24-mer pdt: 23mer+ddD+K		7834.342	0.000	0.0	7,838.00	4.29E+04	2.22	0.46	5	4 - 10	4	37.934	7752 - 7877	21.750	22.100	21.9
13	✓	Component	**25-mer pdt: 23mer+dA+ddD**		8109.454	0.000	0.0	8,113.24	4.47E+05	23.18	4.77	7	5 - 11	7	313.047	7896 - 8073	22.150	22.650	22.4
14		Component			8111.441	0.000	0.0	8,115.23	1.37E+05	7.11	1.46	6	5 - 10	6	315.033	7487 - 7645	20.999	21.450	21.25
15	✓	Component	25-mer pdt: 23mer+dG+ddD		8125.444	0.000	0.0	8,129.24	1.46E+04	0.76	0.16	3	7 - 9	3	329.036	7824 - 7930	21.950	22.249	22.1
16		Component	25-mer pdt: 23mer+dA+ddD+Na		8131.414	0.000	0.0	8,135.21	8.03E+03	0.42	0.09	3	8 - 10	3	335.006	7931 - 8037	22.249	22.549	22.4
17	✓	Component	25-mer pdt: 23mer+dG+dA		8143.434	0.000	0.0	8,147.24	3.67E+04	1.90	0.39	5	5 - 9	4	347.026	7363 - 7504	20.649	21.051	20.8

Results

#	☐	Level	Sequence Name	Modification	Monoisotopic Mass	Theoretical Mass (Da)	Matched Mass Error (ppm)	Average Mass	Sum Intensity	Relative Abundance	Fractional Abundance	Number of Charge States	Charge State Distribution	Number of Detected Intervals	Delta Mass	Scan Range	Start Time (min)	Stop Time (min)	Apex RT
40	✓	Component	**36-mer pdt: 35mer+ddD**		11517.000	0.000	0.0	11,522.38	1.24E+05	6.45	1.33	10	6 - 15	4	0.000	8287 - 8433	23.250	23.650	23.45
41		Component			11519.012	0.000	0.0	11,524.39	1.35E+05	6.98	1.44	8	8 - 15	5	2.012	8002 - 8144	22.450	22.850	22.65
42		Component			11522.975	0.000	0.0	11,528.35	9.04E+04	4.69	0.96	9	6 - 15	3	5.975	8376 - 8494	23.499	23.801	23.65
43	✓	Component	36-mer pdt: 35mer+dA		11534.995	0.000	0.0	11,540.38	1.11E+05	5.75	1.18	9	6 - 14	4	17.995	7949 - 8092	22.301	22.702	22.55
44	✓	Component	36-mer pdt: 35mer+ddD+Na		11538.943	0.000	0.0	11,544.33	6.94E+04	3.60	0.74	6	10 - 15	3	21.943	8376 - 8494	23.499	23.801	23.65
45		Component			11617.116	0.000	0.0	11,622.54	3.25E+04	1.69	0.35	5	6 - 14	4	100.117	8358 - 8494	23.449	23.801	23.6
46		Component			11798.068	0.000	0.0	11,803.57	7.55E+03	0.39	0.08	3	12 - 14	3	281.068	8474 - 8583	23.748	24.049	23.9
47		Component	**37-mer pdt: 35mer+dA+ddD**		11814.077	0.000	0.0	11,819.59	8.35E+05	43.33	8.91	11	6 - 16	7	297.078	8376 - 8566	23.499	24.001	23.75
48	✓	Component	**37-mer pdt: 35mer+dA+ddD**		11830.053	0.000	0.0	11,835.57	8.78E+04	4.55	0.94	5	11 - 15	5	313.053	8340 - 8494	23.399	23.801	23.6
49	✓	Component			11832.051	0.000	0.0	11,837.57	1.17E+05	6.08	1.25	7	7 - 14	6	315.051	8038 - 8198	22.549	23.001	22.75

Supplementary Fig. 21|MS analysis of ddDTP termination products with 51-mer Sanger sequencing template T4 (Supplementary Table 1) which contains MfC at positions 24 and 36. The main components (in red) are the respective desired 24-mer and 36-mer **ddDTP** termination products, as well as a 25-mer and 37-mer formed by incorporation of dATP opposite **MfC** upon which the polymerase stalled and incorporated **ddDTP** before dissociating. Fragments containing dGTP are only found in traces. Assay conducted with high dGTP/low dATP conditions as shown in Supplementary Fig. 22 lanes 5+6 (150 µM dGTP, 37.5 µM dATP, 112.5 µM **ddDTP** (300% relative to dATP)). MS analysis with the Intact Deconvolution Tool of the BioPharma Finder™ software (version 4.1). **Top:** deconvoluted spectrum showing masses of all fragments found (masses in the 16 kDa region correspond to fully elongated 51-mer or 52-mer oligonucleotides). **Centre:** details on the fragments found around 7.8–8.0 kDa which corresponds to 24-mer or 25-mer oligonucleotides). **Bottom:** details on the fragments found around 11.5–11.9 kDa which corresponds to 36-mer or 37-mer oligonucleotides). 37-mers in lines 47 and 48 contain **dA** or **dG** at position 24, respectively. Main components in red. Results of a single experiment (n = 1, not repeated) are shown.

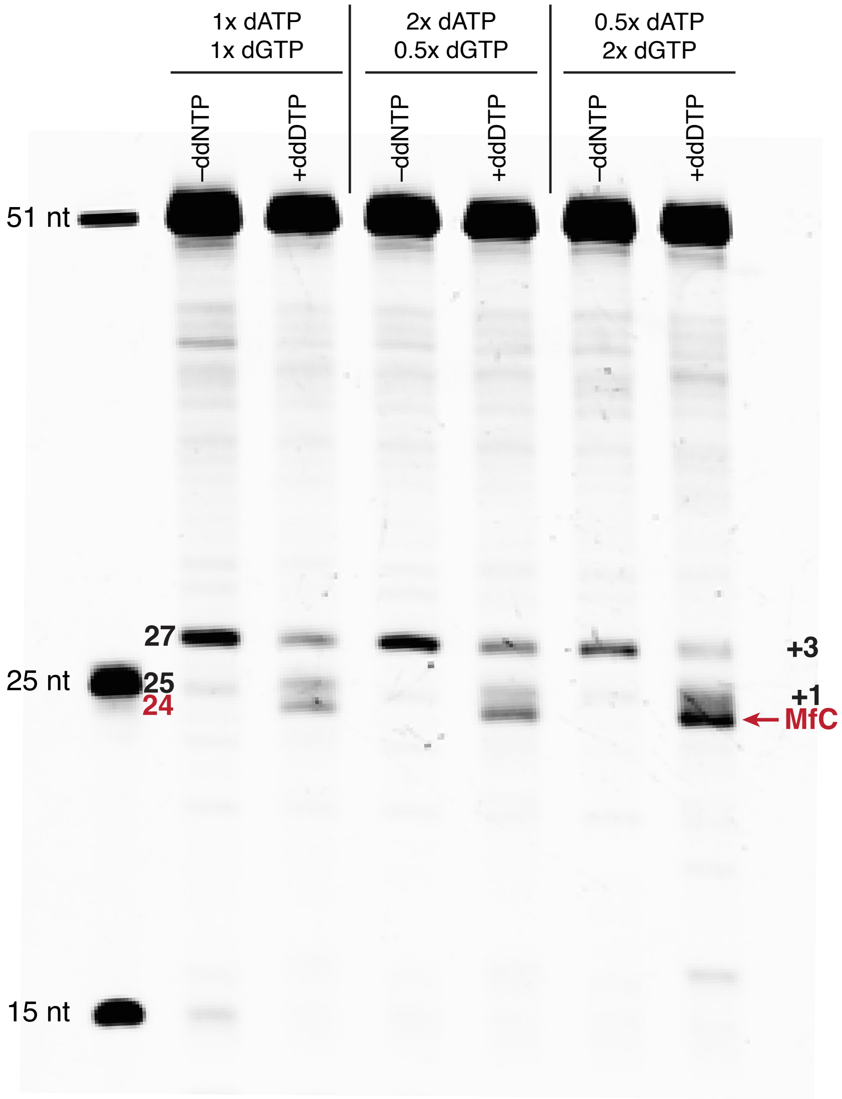

Supplementary Fig. 22|Influence of incorporation of dGTP and dATP opposite MfC on polymerase processivity (template T6; see Supplementary Table 1). Incorporation of dATP partially leads to stalling of the polymerase 3 nucleotides past MfC which can be mitigated by reducing dATP and increasing dGTP concentrations. Assay conditions: 2 U Thermo Sequenase, pH 7.0, 60°C, 2 h. **Lanes 1+2**: equal concentrations of dATP and dGTP (75 µM each); lane 1: control lane with only the four natural dNTPs (all 75 µM); lane 2: four natural dNTPs plus ddDTP (75 µM). **Lanes 3+4**: lower dGTP concentration (37.5 µM) and higher dATP concentration (150 µM); lane 3: control lane with only the four natural dNTPs (75 µM dCTP and dTTP); lane 4: four natural dNTPs plus ddDTP (150 µM). **Lanes 5+6**: lower dATP concentration (37.5 µM) and higher dGTP concentration (150 µM); lane 5: control lane with only the four natural dNTPs (75 µM dCTP and dTTP); lane 6: four natural dNTPs plus ddDTP (150 µM). Results of a single experiment (n = 1, not repeated) are shown.

Synthetic procedures

General experimental details and instrumentation

All reactions for nucleoside and nucleotide synthesis were carried out in flame-dried glassware under a positive pressure of argon gas and magnetically stirred unless otherwise stated. Air- and moisture-sensitive liquids were transferred *via* syringe under argon. All reagents were purchased from commercial suppliers and used without further purification. Reaction solvents were purchased from Acros Organics® as "extra dry" reagents and stored under argon over molecular sieves (4 Å). Chloroform, ethanol and methanol for extraction and flash column chromatography were purchased from Fisher Scientific or VWR in analytical reagent grade and used without further purification. Dichloromethane, ethyl acetate and hexanes were purchased from Fisher Scientific in laboratory reagent grade and distilled under reduced pressure prior to use. Acetonitrile for HPLC purification was purchased from Fisher Scientific in HPLC grade. Water as solvent for reactions, extraction and HPLC purification was obtained as deionised and filtrated water (MilliQ grade).
All reactions were monitored by ESI-LC-MS on a Bruker amaZon X Ion Trap MS, with a Supelcosil LC-18-S nucleoside column (Sigma-Aldrich, 25 cm x 4.6 mm, 5 μm). Flash column chromatography was performed on a Teledyne Combiflash 300 instrument employing pre-packed RediSep High Performance Gold Silica column cartridges (4 g to 120 g) from Teledyne as stationary phase and a forced flow of eluent as mentioned in the specific experiments unless otherwise stated. HPLC purification was performed on an Agilent 1200 Series analytical HPLC equipped with an autosampler, a heated column compartment (40°C), a diode-array detector (monitoring λ = 254 nm and λ = 280 nm). Separation was achieved on a VP NUCLEODUR PolarTec (250 mm/10 mm; 5 μm) from Macherey-Nagel with a gradient of buffer **B** (100 mM triethylammonium acetate in 80% acetonitrile/water) in buffer **A** (100 mM triethylammonium acetate in water, pH 7.0 or pH 9.5) as mentioned in the specific experiments unless otherwise stated.
^{1}H, ^{13}C and ^{31}P NMR characterisation was performed by Andrew Mason, Duncan Howe and Dr Peter Gierth at the Department of Chemistry, either on a Bruker Avance III HD Smart Probe Spectrometer (400 MHz for ^{1}H, 101 MHz for ^{13}C, 162 MHz for ^{31}P) or on a Bruker Avance III Smart Probe Spectrometer (500 MHz for ^{1}H, 126 MHz for ^{13}C, 202 MHz for ^{31}P). ^{1}H-Chemical shifts are reported in ppm (δ scale) relative to $CDCl_3$ (δ_H = 7.26 ppm), D_2O (δ_H = 4.79 ppm) or DMSO-*d6* (δ_H = 2.50 ppm).[1] ^{13}C-Chemical shifts are reported in ppm (δ scale) relative to $CDCl_3$ (δ_C = 77.16 ppm) or DMSO-*d6* (δ_C = 39.52 ppm).[1] HR MS (ESI$^{+/-}$) characterisation was performed by Asha Bodhun, Dr Dijana Matak-Vinkovic and Dr Roberto Canales at the Department of Chemistry, on a Waters LCT Premier Spectrometer, equipped with a Time of Flight analyser. The yields refer to chromatographically and spectroscopically (^{1}H, ^{13}C NMR) pure material.

Synthesis of dD nucleoside

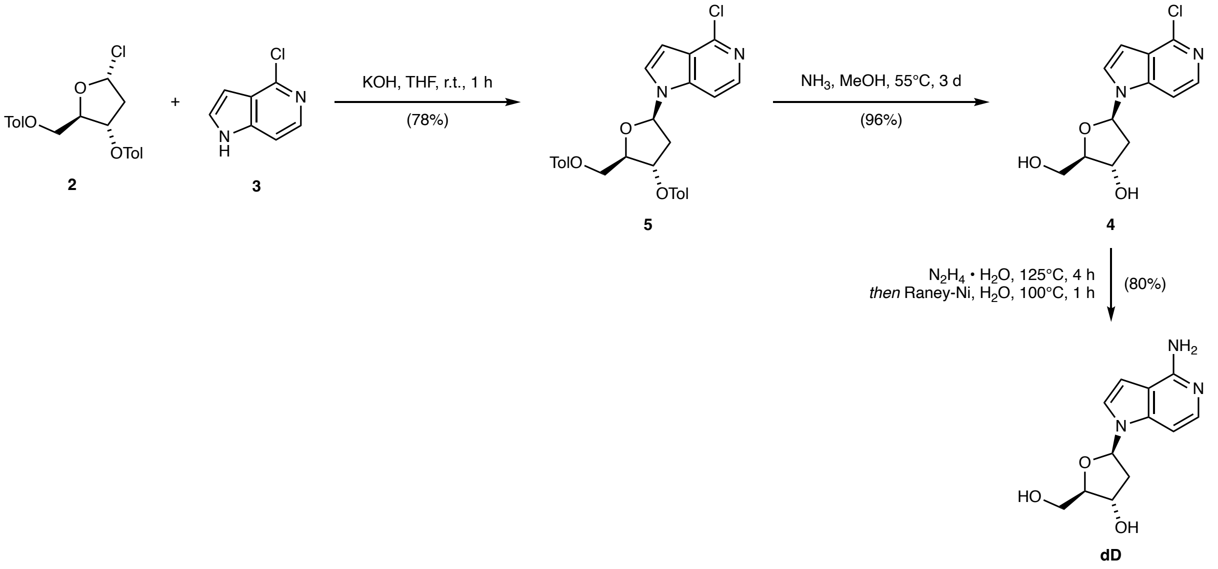

6-Chloro-1-(2-deoxy-*b*-D-*erythro*-pentofuranosyl)-3',5'-di-(*O*-*p*-toluoyl)-1*H*-pyrrolo[3,2-*c*]pyridine (**5**)

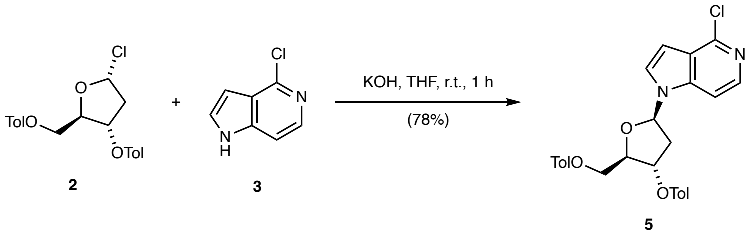

According to a modified literature procedure[2].

Potassium hydroxide (5.90 g, 105 mmol, 6.9 equiv.) was ground into a fine powder and dried under high vacuum overnight. The dried powder was suspended in anhydrous tetrahydrofuran (120 mL) and 4-chloro-1*H*-pyrrolo[3,2-*c*]pyridine (**3**; 2.33 g, 15.3 mmol, 1 equiv.) was added. The mixture was stirred at room temperature for 1 h. 1-Chloro-2-deoxy-3,5-di-*O*-*p*-toluoyl-α-D-ribose **2** (6.02 g, 15.4 mmol, 1.0 equiv.) was added and the reaction suspension stirred at room temperature for another 15 min. The mixture was filtered through a plug of celite, washed with ethyl acetate (500 mL) and the filtrate was purified by flash column chromatography (0% to 10% ethyl acetate in dichloromethane) to afford protected nucleoside **5** (6.00 g, 11.9 mmol, 78%) as a colourless solid.

^{1}H NMR (400 MHz, $CDCl_3$): δ = 8.03 (d, *J* = 5.9 Hz, 1H), 7.97 (d, *J* = 8.3 Hz, 2H), 7.88 (d, *J* = 3.2 Hz, 2H), 7.41 (d, *J* = 6.0 Hz, 1H), 7.38 (d, *J* = 3.5 Hz, 1H), 7.29 (d, *J* = 8.0 Hz, 2H), 7.24 (d, *J* = 7.9 Hz, 2H), 6.68 (d, *J* = 3.5 Hz, 1H), 6.41 (dd, *J* = 8.2, 5.6 Hz, 1H), 5.73 (dt, *J* = 6.4, 2.5 Hz, 1H), 4.75 – 4.68 (m, 1H), 4.66 – 4.58 (m, 2H), 2.83 (ddd, *J* = 14.4, 8.2, 6.4 Hz, 1H), 2.73 (ddd, *J* = 14.2, 5.7, 2.5 Hz, 1H), 2.45 (s, 3H), 2.43 (s, 3H).

^{13}C NMR (101 MHz, $CDCl_3$): δ = 166.3, 166.1, 146.7, 144.8, 144.5, 140.9, 139.7, 130.3, 129.9, 129.7, 129.5, 129.5, 129.3, 126.7, 126.5, 126.2, 124.5, 105.6, 103.4, 86.2, 82.5, 74.8, 64.0, 38.5, 21.9, 21.9.

HR-MS (ESI): calcd for $(C_{28}H_{26}ClN_2O_5)^+$ $[M+H]^+$: 505.1525; found: 505.1547.

6-Chloro-1-(2-deoxy-*b*-D-*erythro*-pentofuranosyl)-1*H*-pyrrolo[3,2-*c*]pyridine (**4**)

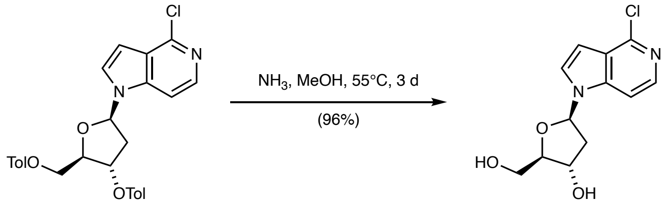

According to a modified literature procedure[2].

Toluoylated nucleoside **5** (3.45 g, 6.83 mmol, 1 equiv.) was dissolved in a solution of ammonia in methanol (7 M, 60 mL) and heated at 55°C in a pressure tube for 3 d. The red solution was then concentrated *in vacuo* and the residue purified by flash column chromatography (0% to 15% methanol in dichloromethane + 1% triethylamine) to afford nucleoside **4** (1.76 g, 6.55 mmol, 96%) as a colourless solid.

^{1}H NMR (400 MHz, DMSO-*d₆*): δ = 8.01 (d, *J* = 5.8 Hz, 1H), 7.83 (d, *J* = 3.5 Hz, 1H), 7.71 (dd, *J* = 5.9, 0.9 Hz, 1H), 6.63 (dd, *J* = 3.3, 0.8 Hz, 1H), 6.41 (dd, *J* = 7.5, 6.1 Hz, 1H), 5.33 (d, *J* = 4.1 Hz, 1H), 4.96 (t, *J* = 5.3 Hz, 1H), 4.38 (dq, *J* = 6.7, 3.3 Hz, 1H), 3.85 (td, *J* f= 4.3, 2.9 Hz, 1H), 3.63 – 3.47 (m, 2H), 2.46 (ddd, *J* = 13.7, 7.5, 6.0 Hz, 1H), 2.28 (ddd, *J* = 13.3, 6.1, 3.2 Hz, 1H).

^{13}C NMR (101 MHz, DMSO-d_6): δ = 142.3, 140.5, 139.8, 128.0, 123.2, 106.3, 101.1, 87.5, 85.2, 70.6, 61.6, 40.0.

HR-MS (ESI): calcd for $(C_{12}H_{14}ClN_2O_3)^+$ [M+H]$^+$: 269.0687, found: 269.0700.

3,7-Dideaza-2'-deoxyadenosine (**dD**)

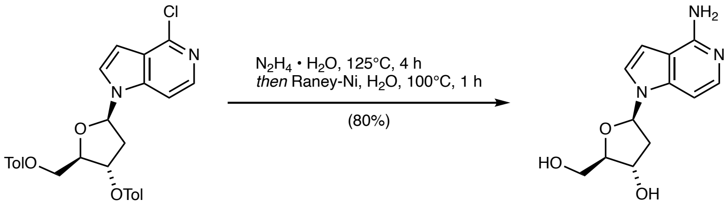

According to a modified literature procedure[2].

Chloronucleoside **4** (503 mg, 1.87 mmol, 1 equiv.) was suspended in hydrazine monohydrate (20 mL) and heated at 125°C for 4 h. The resulting yellow solution was concentrated to ¼ of the original volume under reduced pressure and diluted with degassed water (30 mL). A slurry of Raney-nickel in water (2 mL) was added and the suspension was refluxed for 1 h. The reddish reaction mixture was filtered through a plug of celite, and the filter cake was washed with hot ethanol (60°C, 150 mL). The filtrate was concentrated *in vacuo* and purified by flash column chromatography (0% to 100% **B** in dichloromethane; **B**: 80% chloroform, 19% methanol, 1% concentrated aqueous ammonium hydroxide) to afford **dD** nucleoside (376 mg, 1.51 mmol, 80%) as a slightly brown solid.

^{1}H NMR (400 MHz, DMSO-d_6): δ = 7.55 (d, *J* = 6.0 Hz, 1H), 7.34 (d, *J* = 3.3 Hz, 1H), 6.75 (dd, *J* = 6.0, 0.8 Hz, 1H), 6.64 (d, *J* = 3.3 Hz, 1H), 6.22 (dd, *J* = 7.8, 6.0 Hz, 1H), 6.06 (s, 2H), 5.28 (s, 1H), 4.88 (s, 1H), 4.32 (dt, *J* = 6.3, 3.0 Hz, 1H), 3.80 (td, *J* = 4.7, 3.0 Hz, 1H), 3.51 (qd, *J* = 11.6, 4.9 Hz, 2H), 2.42 (ddd, *J* = 13.6, 7.8, 6.1 Hz, 1H), 2.19 (ddd, *J* = 13.2, 6.0, 3.1 Hz, 1H).

^{13}C NMR (101 MHz, DMSO-d_6): δ = 153.6, 139.9, 139.6, 122.4, 110.7, 101.4, 96.8, 87.0, 84.4, 70.7, 61.9, 39.7.

HR-MS (ESI): calcd for $(C_{12}H_{16}N_3O_3)^+$ [M+H]$^+$: 250.1186, found: 250.1190.

Synthesis of dD phosphoramidite

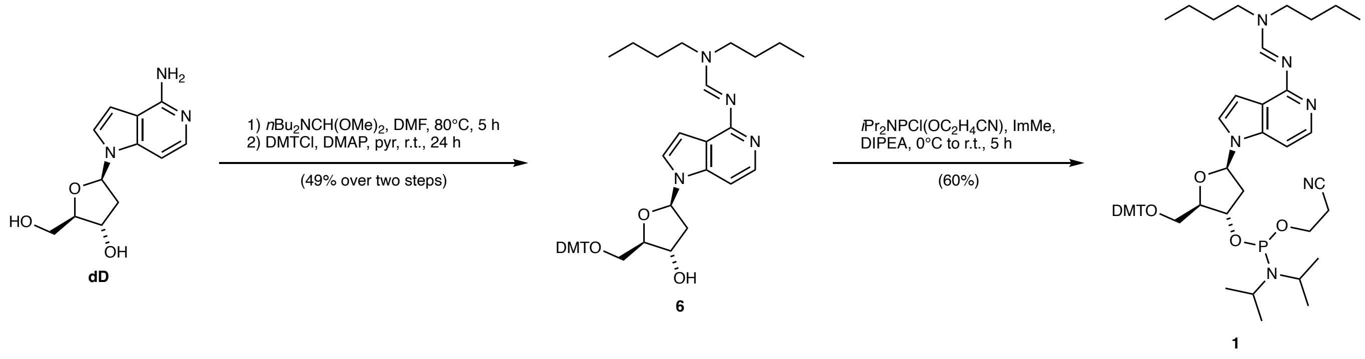

5′-*O*-(4,4′-Dimethoxyltrityl)-6-*N*-(di-*n*-butylaminomethylene)-3,7-dideaza-2′-deoxyadenosine (**6**)

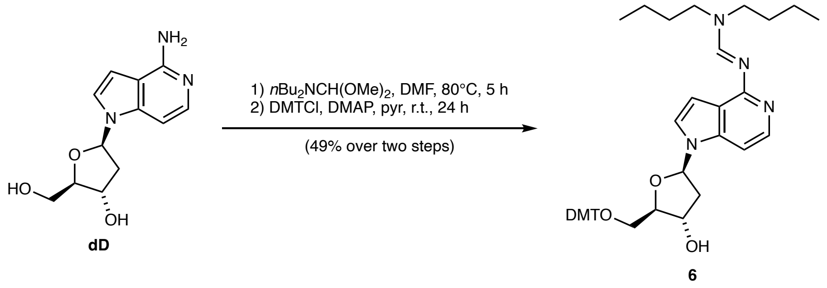

According to modified literature procedures[2,3].
N,N-Dibutylformamide dimethylacetal was synthesised prior to use according to a published protocol[4].

Amidine protection[3]: dD nucleoside (250 mg, 1.00 mmol, 1 equiv.) was dissolved in anhydrous *N,N*-dimethylformamide (10 mL) and di-*n*-butylformamide dimethyl acetal (680 µL, 2.96 mmol, 3.0 equiv.) was added. The reaction mixture was heated at 80°C for 5 h, then allowed to cool to room temperature, and the solvent was removed *in vacuo*. The crude product was purified by flash column chromatography (0% to 100% **B** in dichloromethane; **B**: 85% chloroform, 14% methanol, 1% concentrated ammonium hydroxide) to afford *N*-amidine-protected **dD** (276 mg) as a colourless solid.
DMT protection[2]: The colourless solid (assumed as 1 equiv.) and *N,N*-dimethylaminopyridine (25.0 mg, 0.205 mmol, 0.30 equiv.) were dissolved in anhydrous pyridine (10 mL) and 4,4′-dimethoxytrityl chloride (240 mg, 0.708 mmol, 1.0 equiv.) was added portion-wise at room temperature. The orange reaction mixture was stirred at room temperature overnight and more 4,4′-dimethoxytrityl chloride (116 mg, 0.342 mmol, 0.50 equiv.) was added. After a total of 24 h, the reaction mixture was diluted with methanol (10 mL), upon which the solution turned colourless, and stirred at room temperature for 15 min. Saturated aqueous sodium bicarbonate solution (10 mL) was added until precipitate formed. The mixture was concentrated *in vacuo*, and diluted with ethyl acetate (50 mL), water (20 mL) and saturated aqueous sodium bicarbonate solution (10 mL). The phases were separated, and the aqueous phase was extracted with ethyl acetate (3 x 20 mL). The combined organic layers were washed with saturated aqueous ammonium chloride solution (2 x 30 mL) as well as saturated aqueous sodium chloride solution (30 mL), and dried over magnesium sulfate. The dried solution was filtered, and the filtrate concentrated *in vacuo*. The crude product was purified *via* flash column chromatography (0% to 5% methanol in dichloromethane) to afford protected **dD** nucleoside **6** (338 mg, 0.488 mmol, 49% over two steps) as a colourless solid.

^{1}H NMR (400 MHz, DMSO-*d₆*): δ = 8.60 (s, 1H), 7.77 (d, *J* = 5.8 Hz, 1H), 7.40 (d, *J* = 6.8 Hz, 1H), 7.33 – 7.31 (m, 1H), 7.29 – 7.17 (m, 7H), 7.15 (d, *J* = 5.9 Hz, 1H), 7.13 – 7.05 (m, 1H), 6.86 – 6.69 (m, 4H), 6.51 (d, *J* = 3.3 Hz, 1H), 6.34 (t, *J* = 6.4 Hz, 1H), 5.36 (d, *J* = 4.7 Hz, 1H), 4.38 (p, *J* = 4.7 Hz, 1H), 3.93 (q, *J* = 4.4 Hz, 1H), 3.71 (s, 3H), 3.70 (s, 3H), 3.52 (t, *J* = 7.4 Hz, 2H), [second N-CH2 signal beneath water peak], 3.16 – 3.06 (m, 2H), 2.60 – 2.52 (m, 1H), 2.29 (ddd, *J* = 13.3, 6.5, 4.6 Hz, 1H), 1.66 – 1.48 (m, 4H), 1.40 – 1.21 (m, 4H), 0.92 (tq, *J* = 11.5, 7.5 Hz, 6H).

^{13}C NMR (101 MHz, DMSO-d_6): δ = 158.0, 158.0, 155.8, 154.5, 144.9, 140.7, 139.4, 135.5, 135.5, 131.8, 129.8, 129.7, 127.8, 127.7, 126.6, 123.7, 118.9, 113.1, 101.9, 101.2, 85.4, 85.1, 84.5, 70.5, 64.0, 55.0, 55.0, 50.5, 44.1, 39.9, 30.8, 28.7, 19.7, 19.3, 13.8, 13.7.

HR-MS (ESI): calcd for $(C_{42}H_{51}N_4O_5)^+$ $[M+H]^+$: 691.3854, found: 691.3841.

5'-*O*-(4,4'-Dimethoxyltrityl)-6-*N*-(di-*n*-butylaminomethylene)-3,7-dideaza-2'-deoxyadenosine-3'-(*O*-(2-cyanoethyl)-*N*-di-*iso*-propyl)phosphoramidite (**1**)

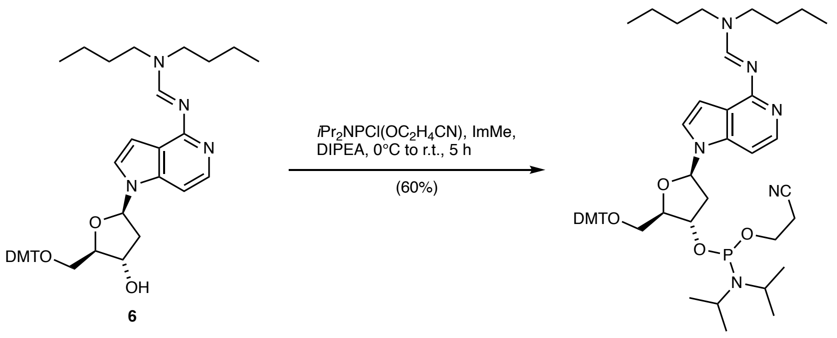

According to a modified literature procedure[5].

Protected **dD** nucleoside **6** (94.6 mg, 0.137 mmol, 1 equiv.) was dried in a vacuum oven overnight and was then dissolved in anhydrous dichloromethane (2.0 mL). Di-*iso*-propylethylamine (100 µL, 0.58 mmol, 4.0 equiv.) and 1-methylimidazole (6.0 µL, 72 µmol, 0.5 equiv.) were added and the solution cooled to 0°C. 2-Cyanoethyl-*N,N*-di-*iso*-propylchlorophosphoramidite (37 µL, 0.17 mmol, 1.2 equiv.) was added and the reaction mixture stirred at 0°C for 10 min. The solution was allowed to warm to room temperature and stirred for another 5 h. Methanol (10 mL) was added and the mixture was stirred for 30 min upon which the solvents were removed *in vacuo* and the residue re-dissolved in dichloromethane (200 mL). The organic solution was washed with saturated aqueous sodium bicarbonate solution (200 mL) and dried over sodium sulfate. The dried solution was filtered and the filtrate concentrated *in vacuo*. The crude product was purified by flash column chromatography (0% to 5% methanol in dichloromethane + 0.1% triethylamine) to afford phosphoramidite **1** (76.0 mg, 85.3 µmol, 60%) as a yellow oil.

^{1}H NMR (500 MHz, $CDCl_3$): not assigned as complicated due to presence of diastereomers on phosphorus.

^{13}C NMR (126 MHz, $CDCl_3$): not assigned as complicated due to presence of diastereomers on phosphorus.

^{31}P NMR (203 MHz, $CDCl_3$): δ = 148.8, 148.6.

HR-MS (ESI): calcd for $(C_{51}H_{68}N_6O_6P)^+$ $[M+H]^+$: 891.4932, found: 891.4897.

Synthesis of dDTP 2'-deoxynucleoside triphosphate

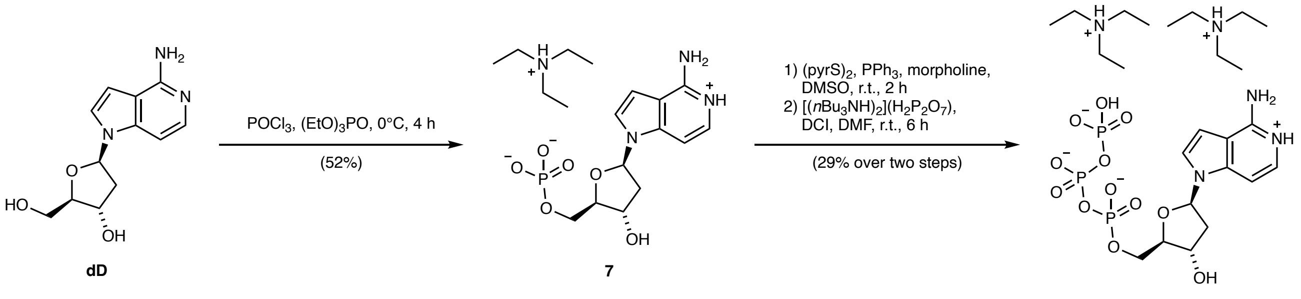

3,7-Dideaza-2'-deoxyadenosine-5'-monophosphate triethylammonium salt (**7**)

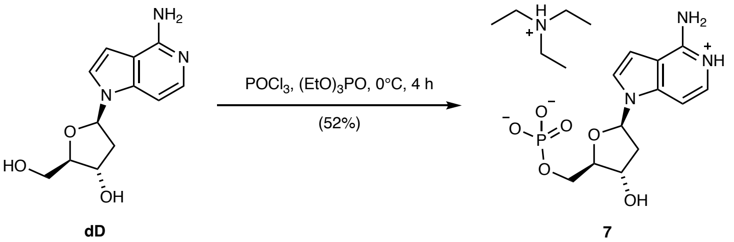

According to a modified literature procedure[6].

3,7-Dideaza-2'-deoxyadenosine (99.7 mg, 0.400 mmol, 1 equiv.) was dissolved in triethyl phosphate (1.6 mL) and the solution cooled to 0°C. Phosphoryl chloride (55 μL, 0.60 mmol, 1.5 equiv.) was mixed with triethyl phosphate (55 μL) and then added dropwise to the reaction solution, and the mixture was stirred at 0°C. After 1.5 h and again after 3 h, more phosphoryl chloride in triethyl phosphate (50%, 0.11 mL, 0.60 mmol, 1.5 equiv.) was added, respectively. After 4 h in total, the reaction mixture was neutralised by addition of aqueous triethylammonium acetate buffer pH 7.0 (0.1 M, 30 mL) and stirred for 15 min before being allowed to warm to room temperature. The solvents were removed *via* lyophilisation and the remaining crude product purified *via* HPLC (5% to 8% buffer **B** over 12 min; buffer **A** at pH 7.0) to afford 3,7-dideaza-2'-deoxyadenosine-5'-monophosphate triethylammonium salt **7** (79 wt%, 141 mg, 0.210 mmol, 52%) with minor contaminations of triethylammonium acetate.

^{1}H NMR (500 MHz, D$_2$O): δ = 7.62 (d, J = 3.6 Hz, 1H), 7.38 (d, J = 7.2 Hz, 1H), 7.07 (d, J = 7.3 Hz, 1H), 6.79 (d, J = 3.6 Hz, 1H), 6.41 (dd, J = 7.8, 6.2 Hz, 1H), 4.70 (dt, J = 6.2, 3.1 Hz, 1H), 4.24 (qd, J = 3.4, 1.7 Hz, 1H), 4.06 (dd, J = 5.2, 3.6 Hz, 2H), 3.20 (q, J = 7.3 Hz, 12H), 2.68 (ddd, J = 14.0, 7.8, 6.2 Hz, 1H), 2.49 (ddd, J = 14.0, 6.2, 3.1 Hz, 1H), 1.28 (t, J = 7.3 Hz, 18H).

^{13}C NMR (126 MHz, D$_2$O): δ = 148.9, 139.8, 127.0, 126.0, 109.9, 103.5, 99.0, 85.5 (d), 85.4, 71.3, 64.7 (d), 46.6, 39.3, 8.2.

^{31}P NMR (202 MHz, D$_2$O): δ = 0.72.

HR-MS (ESI): calcd for $(C_{12}H_{17}N_3O_6P)^+$ $[M+H]^+$: 330.0849, found: 330.0849.

3,7-Dideaza-2′-deoxyadenosine-5′-triphosphate triethylammonium salt (**dDTP**)

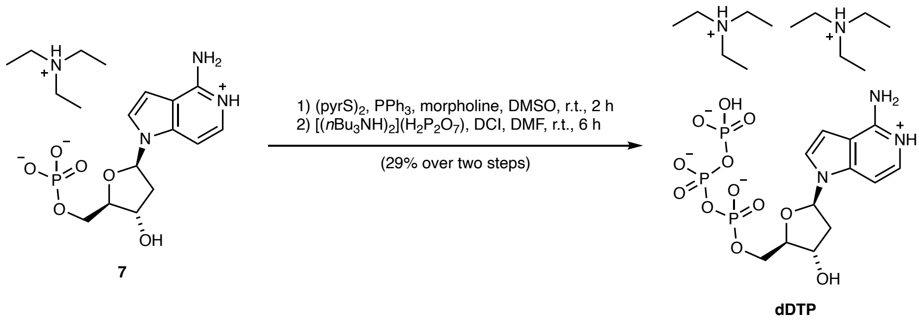

According to modified literature procedures[6,7].

Morpholidation[6]: 3,7-Dideaza-2′-deoxyadenosine-5′-monophosphate **7** (79 wt%, 32.8 mg, 48.8 µmol, 1 equiv.) was suspended in anhydrous dimethylsulfoxide (0.5 mL) and the suspension was co-evaporated with anhydrous *N,N*-dimethylformamide (2 x 0.4 mL). The remaining solid was re-suspended in anhydrous dimethylsulfoxide (0.5 mL), anhydrous morpholine (65 µL, 0.75 mmol, 15 equiv.) was added, and the suspension was stirred at room temperature for 5 min. Triphenylphosphine (128 mg, 0.488 mmol, 10.0 equiv.) and 2,2′-dipyridyl disulphide (108 mg, 0.488 mmol, 10.0 equiv.) were added and the resulting yellow solution was stirred at room temperature. After 2 h, a solution of sodium iodide in acetone (0.1 M, 10 mL) was added and the mixture was cooled at –80°C overnight. The formed precipitate was collected *via* centrifugation and washed with –20°C-cold acetone (2 x 3 mL). The crude product was dried under high vacuum for one hour and directly used for the next step.
Pyrophosphorylation[7]: The crude 3,7-dideaza-2′-deoxyadenosine-5′-phosphomorpholidate was dissolved in anhydrous *N,N*-dimethylformamide (0.5 mL) and bis(triethylammonium) dihydrogenpyrophosphate (67.0 mg, 0.122 µmol, 2.5 equiv.) followed by 4,5-dicyanoimidazole (34.6 mg, 0.293 mmol, 6.0 equiv.) were added. The reaction solution turned turbid after 5 min and was further stirred at room temperature for 6 h. The crude product was dried under high vacuum overnight. The crude product was re-dissolved in aqueous sodium chloride solution (0.3 M, 1.0 mL), ethanol (10 mL) was added and the resulting suspension cooled to –80°C for 1.5 h for precipitation. The precipitate was collected *via* centrifugation and purified *via* HPLC (5% to 8% buffer **B** over 12 min; buffer **A** at pH 7.0) to afford 3,7-dideaza-2′-deoxyadenosine-5′-triphosphate triethylammonium salt **dDTP** (63 wt% neutral triphosphoric acid, 11.0 mg, 14.1 µmol, 29% over 2 steps) with minor contaminations of triethylammonium acetate.

^{1}H NMR (500 MHz, D$_2$O): δ = 7.69 (d, J = 3.6 Hz, 1H), 7.49 (d, J = 7.2 Hz, 1H), 7.17 (d, J = 7.2 Hz, 1H), 6.90 (d, J = 3.5 Hz, 1H), 6.46 (t, J = 6.8 Hz, 1H), 4.83 – 4.80 (m, 1H), 4.29 – 4.22 (m, 2H), 4.18 (ddd, J = 13.0, 5.8, 3.1 Hz, 1H), 3.27 (q, J = 7.3 Hz, 4H), 3.21 (q, J = 7.3 Hz, 12H), 2.70 (ddd, J = 14.0, 7.6, 6.3 Hz, 1H), 2.49 (ddd, J = 14.0, 6.3, 3.4 Hz, 1H), 1.28 (t, J = 7.4 Hz, 20H), 1.25 (t, J = 7.2 Hz, 6H).

^{13}C NMR (126 MHz, D$_2$O): δ = 149.2, 139.9, 127.1, 126.2, 110.1, 103., 99.2, 85.5, 85.4, 70.9, 65.3 (d), 58.9, 46.6, 39.2, 8.2, 7.4.

^{31}P NMR (162 MHz, D$_2$O): δ = –8.5 (br s), –11.3 (d, J = 19.7 Hz), –22.8 (t, J = 19.8 Hz).

HR-MS (ESI): calcd for $(C_{12}H_{19}N_3O_{12}P_3)^+$ $[M+H]^+$: 490.0176, found: 490.0168.

Synthesis of ddDTP 2',3'-dideoxynucleoside triphosphate

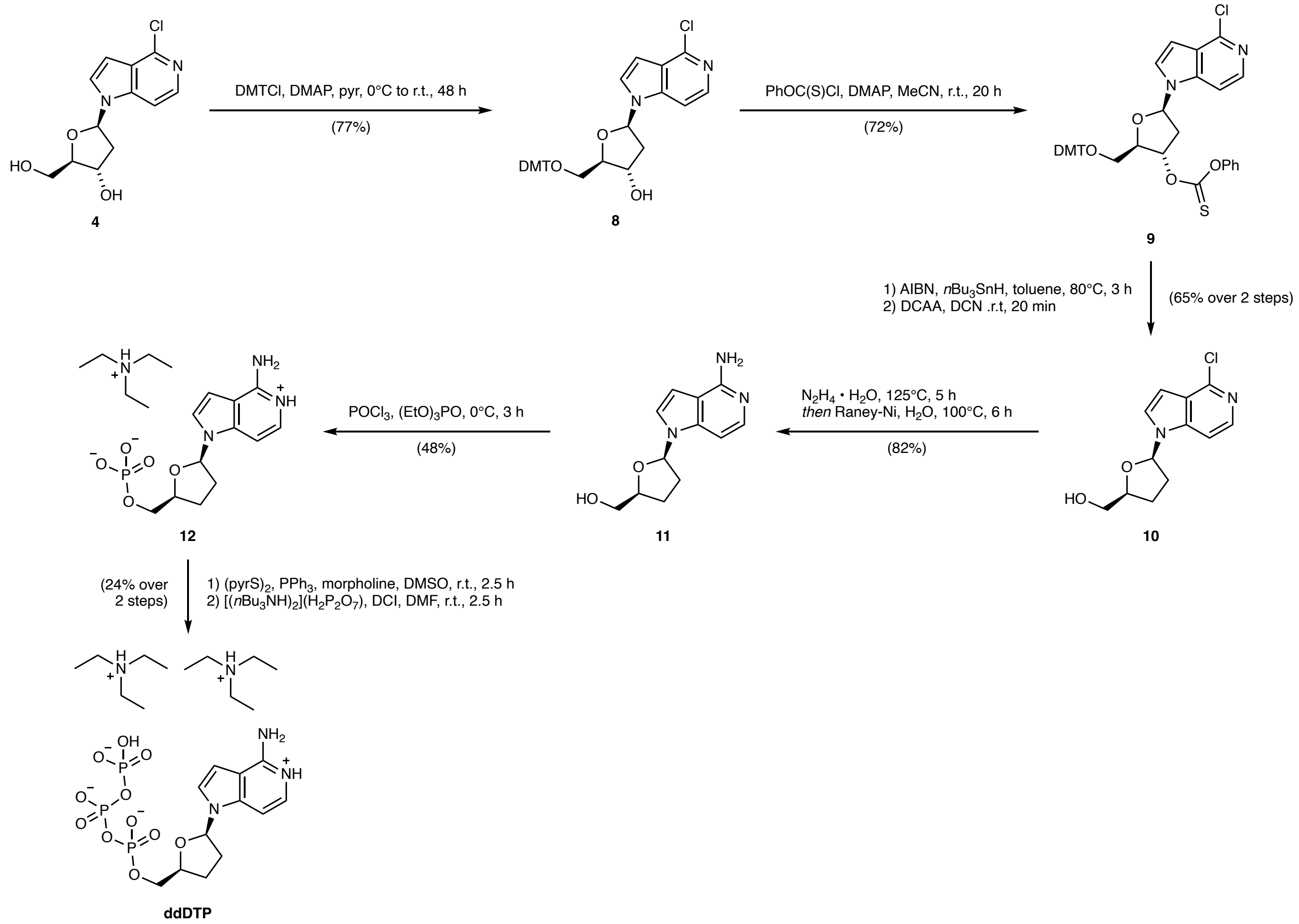

6-Chloro-1-(2-deoxy-*b*-D-*erythro*-pentofuranosyl)-5'-*O*-(4,4'-dimethoxytrityl)-1*H*-pyrrolo[3,2-*c*]pyridine (**8**)

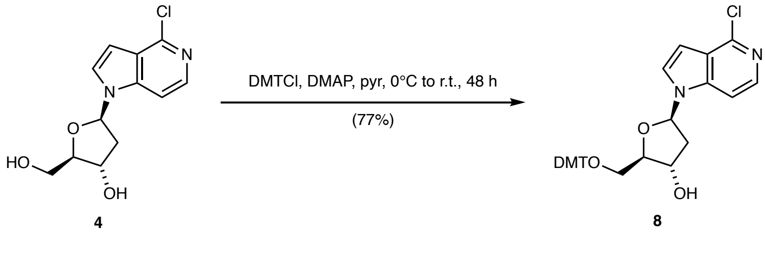

According to a modified literature procedure[2].

Chloronucleoside **4** (446 mg, 1.66 mmol, 1 equiv.) was dissolved in anhydrous pyridine (60 mL), *N,N*-dimethylaminopyridine (30.4 mg, 0.249 mmol, 0.15 equiv.) was added, and the solution was cooled to 0°C. 4,4'-Dimethoxytrityl chloride (281 mg, 0.830 mmol, 0.50 equiv.) was added upon which the reaction mixture turned yellow, and the solution was stirred at 0°C. After 4 h, more 4,4'-dimethoxytrityl chloride (281 mg, 0.830 mmol, 0.50 equiv.) was added and further stirred overnight while slowly warming to room temperature. After 24 h, more 4,4'-dimethoxytrityl chloride (112 mg, 0.332 mmol, 0.20 equiv.) was added at 0°C and the reaction mixture allowed to warm to room temperature. After a total of 48 h, the reaction mixture was diluted with methanol (150 mL), upon which the solution turned colourless, and stirred at room temperature for 15 min. Saturated aqueous sodium bicarbonate solution (20 mL) was added until precipitate formed. The mixture was concentrated *in vacuo*, and diluted with ethyl acetate (250 mL), water (100 mL) and saturated aqueous sodium bicarbonate solution (30 mL). The phases were separated, and the

aqueous phase was extracted with ethyl acetate (3 x 80 mL). The combined organic layers were washed with saturated aqueous ammonium chloride solution (2 x 100 mL) as well as saturated aqueous sodium chloride solution (100 mL), and dried over magnesium sulfate. The dried solution was filtered, and the filtrate concentrated *in vacuo*. The crude product was purified *via* flash column chromatography (20% to 80% ethyl acetate in hexanes) to afford 5'-protected chloronucleoside **8** (726 mg, 1.27 mmol, 77%) as a yellowish solid.

^{1}H NMR (700 MHz, DMSO-d$_6$): δ 7.96 (d, *J* = 5.7 Hz, 1H), 7.71 (d, *J* = 5.8 Hz, 1H), 7.68 (d, *J* = 3.4 Hz, 1H), 7.29 (d, *J* = 7.3 Hz, 2H), 7.20 (dt, *J* = 13.6, 6.8 Hz, 3H), 7.17 – 7.14 (m, 4H), 6.77 (dd, *J* = 10.6, 8.8 Hz, 4H), 6.60 (d, *J* = 3.4 Hz, 1H), 6.44 (t, *J* = 6.2 Hz, 1H), 5.44 (d, *J* = 5.0 Hz, 1H), 4.42 (dt, *J* = 9.7, 4.8 Hz, 1H), 3.95 (q, *J* = 4.6 Hz, 1H), 3.71 (s, 6H), 3.13 (dd, *J* = 10.4, 3.2 Hz, 1H), 3.08 (dd, *J* = 10.4, 5.6 Hz, 1H), 2.61 (dt, *J* = 12.9, 6.3 Hz, 1H), 2.39 – 2.34 (m, 1H).

^{13}C NMR (176 MHz, DMSO-*d$_6$*): δ 158.1, 158.1, 144.9, 142.5, 140.6, 139.9, 135.5, 135.5, 129.8, 129.8, 127.9, 127.8, 127.8, 126.8, 123.5, 113.2, 106.8, 101.1, 85.6, 85.5, 85.1, 70.3, 63.8, 55.1, 55.1, 39.4.

HR-MS (ESI): calcd for $(C_{33}H_{32}ClN_2O_5)^+$ [M+H]$^+$: 571.1994, found: 571.1976.

6-Chloro-1-(2-deoxy-*b*-D-*erythro*-pentofuranosyl)-5'-*O*-(4,4'-dimethoxytrityl)-3'-*O*-phenoxythiocarbonyl-1*H*-pyrrolo[3,2-*c*]pyridine (**9**)

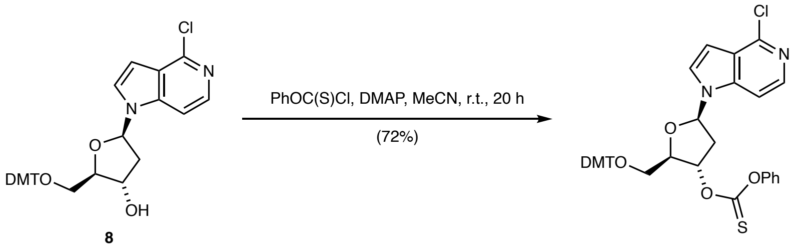

According to a modified literature procedure[2].

5'-Protected chloronucleoside **8** (595 mg, 1.04 mmol, 1 equiv.) and *N,N*-dimethylaminopyridine (600 mg, 4.91 mmol, 4.70 equiv.) were dissolved in anhydrous acetonitrile (15 mL), *O*-phenylchlorothionocarbonate (0.32 mL, 2.3 mmol, 2.2 equiv.) was added dropwise, and the yellow solution stirred at room temperature overnight. After 20 h, the reaction mixture was diluted with ethyl acetate (50 mL) and saturated aqueous sodium bicarbonate solution (20 mL) as well as water (20 mL) were added upon which the biphasic mixture was stirred at room temperature for 15 min. The layers were separated and the aqueous layer was extracted with ethyl acetate (3 x 30 mL). The combined organic layers were washed with saturated aqueous ammonium chloride solution (50 mL) and saturated aqueous sodium chloride solution (50 mL) and dried over magnesium sulphate. The dried solution was filtered and the filtrate concentrated *in vacuo*. The crude product was purified *via* flash column chromatography (20% to 100% ethyl acetate in hexanes) to afford 3'-thionocarbonate **9** (532 mg, 0.752 mmol, 72%).

^{1}H NMR (700 MHz, DMSO-d$_6$): δ 7.90 (d, *J* = 5.8 Hz, 1H), 7.80 (dd, *J* = 5.9, 0.9 Hz, 1H), 7.71 (d, *J* = 3.5 Hz, 1H), 7.49 (dd, *J* = 8.6, 7.5 Hz, 2H), 7.37 – 7.33 (m, 3H), 7.29 – 7.24 (m, 3H), 7.23 – 7.20 (m, 7H), 7.07 (d, *J* = 8.8 Hz, 1H), 6.83 (dd, *J* = 8.9, 7.7 Hz, 5H), 6.65 (dd, *J* = 3.4, 0.9 Hz, 1H), 6.56 (dd, *J* = 8.7, 5.6 Hz, 1H), 5.93 (d, *J* = 5.9 Hz, 1H), 4.43 (t, *J* = 5.2 Hz, 1H), 3.31 (dd, *J* = 10.5, 3.3 Hz, 1H), [second H-5' beneath water peak], 3.01 – 2.96 (m, 1H), 2.85 (dd, *J* = 14.3, 5.7 Hz, 1H).

^{13}C NMR (176 MHz, DMSO-*d$_6$*): δ 193.8, 158.2, 153.0, 144.6, 142.5, 140.6, 140.3, 135.3, 135.2, 129.9, 129.8, 129.8, 129.7, 129.0, 128.0, 127.8, 127.7, 127.7, 127.0, 126.9, 123.5, 122.0, 121.9, 121.3, 113.4, 113.3, 113.3, 113.2, 113.1, 112.8, 106.9, 101.5, 86.1, 85.7, 84.2, 82.8, 63.8, 55.1, 36.8.

HR-MS (ESI): calcd for $(C_{40}H_{36}ClN_2O_6S)^+$ [M+H]$^+$: 707.1977, found: 707.1971.

6-Chloro-1-(2,3-dideoxy-*b*-D-*erythro*-pentofuranosyl)-1*H*-pyrrolo[3,2-*c*]pyridine (**10**)

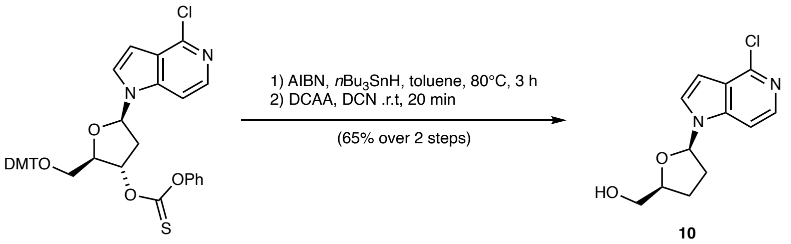

9 **10**

According to a modified literature procedure[2].

Deoxygenation: 3'-Thionocarbonate **9** (465 mg, 0.658 mmol, 1 equiv.) was dissolved in anhydrous toluene and 2,2'-azobis(2-methylpropionitrile) (54.0 mg, 0.329 mmol, 0.50 equiv.) was added. Tri-*n*-butyltin hydride (0.39 mL, 1.45 mmol, 2.2 equiv.) was added dropwise and the reaction solution was heated at 80°C for 3 h. The mixture was allowed to cool to room temperature and concentrated *in vacuo*. The crude product was purified *via* flash column chromatography (20% to 50% ethyl acetate in hexanes) to produce the deoxygenated intermediate as a foamy, colourless solid (ca 330 mg).

5'-Deprotection: The purified intermediate was then dissolved in a solution of dichloroacetic acid in dichloromethane (5%, 10 mL) and the resulting red-orange solution was stirred at room temperature for 20 min. Methanol (10 mL) was added followed by dropwise addition of saturated aqueous sodium bicarbonate solution (20 mL). The biphasic mixture was stirred at room temperature for 10 min and then diluted with water (20 mL). The layers were separated, and the aqueous layer was extracted with ethyl acetate (5 x 30 mL). The combined organic layers were washed with saturated aqueous sodium bicarbonate solution (30 mL) and saturated aqueous sodium chloride solution (40 mL), and dried over magnesium sulphate. The dried solution was filtered, and the filtrate concentrated *in vacuo*. The crude product was purified *via* flash column chromatography (0% to 5% methanol in dichloromethane) to afford chlorodideoxynucleoside **10** (108 mg, 0.428 mmol, 65% over two steps) as a colourless solid.

^{1}H NMR (400 MHz, DMSO-d$_6$): δ = 8.01 (d, J = 5.8 Hz, 1H), 7.83 (d, J = 3.4 Hz, 1H), 7.66 (d, J = 5.8 Hz, 1H), 6.61 (d, J = 3.3 Hz, 1H), 6.32 (dd, J = 6.8, 4.0 Hz, 1H), 4.90 (t, J = 5.4 Hz, 1H), 4.09 (ddt, J = 8.3, 6.7, 4.4 Hz, 1H), 3.55 (ddd, J = 11.5, 5.7, 4.1 Hz, 1H), 3.47 (dt, J = 11.6, 5.0 Hz, 1H), 2.42 (dtd, J = 13.1, 8.6, 6.8 Hz, 1H), 2.23 (ddt, J = 12.9, 8.5, 4.4 Hz, 1H), 2.10 – 1.93 (m, 2H).

^{13}C NMR (101 MHz, DMSO-*d$_6$*): δ = 142.3, 140.2, 139.6, 127.7, 123.1, 106.3, 100.7, 85.7, 81.1, 63.0, 31.7, 26.0.

HR-MS (ESI): calcd for $(C_{12}H_{14}ClN_2O_2)^+$ $[M+H]^+$: 253.0738, found: 253.0750.

3,7-Dideaza-2',3'-dideoxyadenosine (**11**)

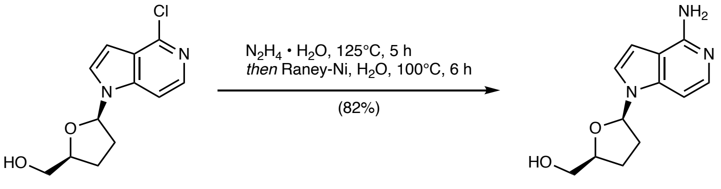

10 **11**

According to a modified literature procedure[2].

Chlorodideoxynucleoside **10** (64.8 mg, 0.256 mmol. 1 equiv.) was suspended in hydrazine monohydrate (15 mL) and the suspension was heated at 125°C for 5 h. The yellow mixture was allowed to cool to room temperature and concentrated *in vacuo* to ¼ of the original volume. The resulting yellow solution was diluted with degassed water (15 mL), a slurry of Raney-nickel in water (2 mL) was added and the reaction suspension was refluxed for 6 h. The mixture was allowed to

cool to room temperature and filtered through a plug of celite. The filter cake was washed with hot ethanol (60°C, 60 mL) and the filtrate concentrated *in vacuo*. The crude product was purified *via* flash column chromatography (50% to 80% **B** in dichloromethane; **B**: 85% chloroform, 14% methanol, 1% concentrated ammonium hydroxide solution) to afford 3,7-dideaza-2',3'-dideoxyadenosine **11** (49.1 mg, 0.210 mmol, 82%) as a colourless solid.

^{1}H NMR (500 MHz, DMSO-d$_6$): δ = 7.85 (s, 2H), 7.70 (d, J = 3.4 Hz, 1H), 7.58 (d, J = 7.0 Hz, 1H), 7.19 (d, J = 7.0 Hz, 1H), 6.99 (d, J = 3.3 Hz, 1H), 6.27 (dd, J = 6.8, 3.9 Hz, 1H), 4.89 (t, J = 5.4 Hz, 1H), 4.09 (ddt, J = 8.3, 6.6, 4.4 Hz, 1H), 3.55 (ddd, J = 11.7, 5.5, 4.1 Hz, 1H), 3.47 (dt, J = 11.6, 4.9 Hz, 1H), 2.42 (dtd, J = 13.2, 8.7, 6.8 Hz, 1H), 2.19 (ddt, J = 12.8, 8.4, 4.2 Hz, 1H), 2.10 – 1.89 (m, 2H).

^{13}C NMR (126 MHz, DMSO-*d$_6$*): δ = 149.8, 138.9, 128.9, 125.5, 109.8, 103.2, 99.0, 85.6, 81.3, 62.9, 31.9, 25.9.

HR-MS (ESI): calcd for $(C_{12}H_{16}N_3O_2)^+$ [M+H]$^+$: 234.1237, found: 234.1228.

3,7-Dideaza-2',3'-dideoxyadenosine-5'-monophosphate triethylammonium salt (**12**)

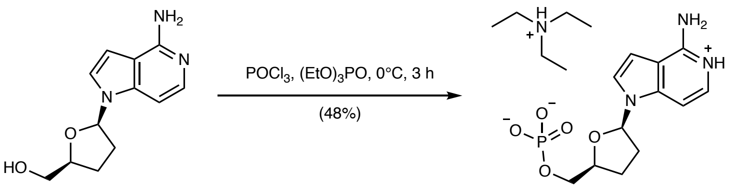

11 **12**

According to a modified literature procedure[6].

3,7-Dideaza-2',3'-dideoxyadenosine **11** (66.7 mg, 0.286 mmol, 1 equiv.) was dissolved in triethyl phosphate (1.0 mL) and cooled to 0°C. A freshly prepared solution of phosphoryl chloride in triethyl phosphate (50%, 0.16 mL, 0.86 mmol, 3.0 equiv.) was added dropwise and the solution stirred at 0°C. After 2 h, more of the phosphoryl chloride solution in triethyl phosphate (50%, 55 µL, 0.29 mmol, 1.0 equiv.) was added and the mixture stirred at 0°C for another 1 h. After 3 h in total, the reaction solution was neutralised with aqueous triethylammonium acetate buffer pH 7.0 (0.1 M, 30 mL) and stirred for another 15 min. The solution was concentrated by lyophilisation and the crude product was purified *via* HPLC (5% to 8% buffer **B** over 12 min; buffer **A** at pH 7.0) to afford 3,7-dideaza-2',3'-dideoxyadenosine-5'-monophosphate triethylammonium salt **12** (89 wt%, 78.8 mg, 0.136 mmol, 48%) with minor contaminations of triethylammonium acetate.

^{1}H NMR (500 MHz, D$_2$O): δ = 7.58 (d, J = 3.6 Hz, 1H), 7.31 (d, J = 7.2 Hz, 1H), 6.96 (d, J = 7.2 Hz, 1H), 6.71 (d, J = 3.5 Hz, 1H), 6.21 (dd, J = 7.1, 3.9 Hz, 1H), 4.46 – 4.38 (m, 1H), 4.08 (ddd, J = 11.5, 5.2, 2.9 Hz, 1H), 3.92 (dt, J = 11.2, 5.4 Hz, 1H), 3.20 (q, J = 7.3 Hz, 6H), 2.56 (dtd, J = 13.5, 8.8, 7.0 Hz, 1H), 2.34 (ddt, J = 13.1, 8.3, 4.2 Hz, 1H), 2.29 – 2.13 (m, 2H), 1.28 (t, J = 7.3 Hz, 9H).

^{13}C NMR (126 MHz, D$_2$O): δ = 148.7, 139.3, 126.8, 125.8, 109.5, 103.0, 98.8, 85.8, 80.4 (d), 65.8 (d), 46.6, 31.5, 25.6, 8.2.

^{31}P NMR (162 MHz, D$_2$O): δ = 2.17.

HR-MS (ESI): calcd for $(C_{12}H_{15}N_3O_5P)^-$ [M–H]$^-$: 312.0755, found: 312.0756.

3,7-Dideaza-2′,3′-dideoxyadenosine-5′-triphosphate triethylammonium salt (**ddDTP**)

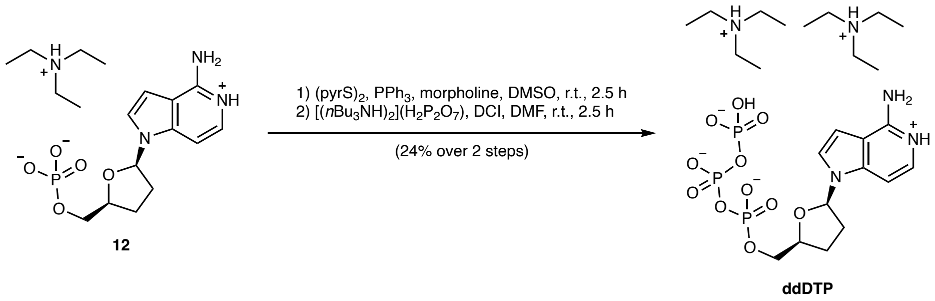

According to modified literature procedures[6,7].

Morpholidation[6]: 3,7-Dideaza-2′,3′-dideoxyadenosine-5′-monophosphate triethyl ammonium salt **12** (89 wt%, 6.2 mg, 13 µmol, 1 equiv.) was suspended in anhydrous dimethylsulfoxide (0.5 mL) and the suspension was co-evaporated with anhydrous *N,N*-dimethylformamide (2 x 0.4 mL). The remaining solid was re-suspended in anhydrous dimethylsulfoxide (0.5 mL), anhydrous morpholine (17 µL, 0.20 mmol, 15 equiv.) was added, and the suspension was stirred at room temperature for 5 min. Triphenylphosphine (34.1 mg, 0.130 mmol, 10.0 equiv.) and 2,2′-dipyridyl disulphide (28.6 mg, 0.130 mmol, 10.0 equiv.) were added and the resulting yellow solution was stirred at room temperature. After 2.5 h, a solution of sodium iodide in acetone (0.1 M, 10 mL) was added and the mixture was cooled at –80°C overnight. The formed precipitate was collected *via* centrifugation and washed with –20°C-cold acetone (2 x 3 mL). The crude product was dried under high vacuum for one hour and directly used for the next step.

Pyrophosphorylation[7]: The crude 3,7-dideaza-2′-deoxyadenosine-5′-phosphomorpholidate was dissolved in anhydrous *N,N*-dimethylformamide (0.5 mL) and bis(triethylammonium) dihydrogenpyrophosphate (17.8 mg, 32.5 µmol, 2.5 equiv.) followed by 4,5-dicyanoimidazole (9.2 mg, 78 µmol, 6.0 equiv.) were added. The reaction solution turned turbid after 5 min and was further stirred at room temperature for 2.5 h. The crude product was dried under high vacuum for 2 h. The crude product was re-dissolved in aqueous sodium chloride solution (0.3 M, 1.0 mL), ethanol (10 mL) was added and the resulting suspension cooled to –80°C for 1.5 h for precipitation. The precipitate was collected *via* centrifugation and purified *via* HPLC (5% to 8% buffer **B** over 12 min; buffer **A** at pH 9.5) to afford 3,7-dideaza-2′-deoxyadenosine-5′-triphosphate triethylammonium salt **ddDTP** (74 wt% neutral triphosphoric acid, 2.0 mg, 3.1 µmol, 24% over 2 steps) with minor contaminations of triethylammonium acetate.

^{1}H NMR (500 MHz, D$_2$O): *δ* = 7.67 (d, *J* = 3.5 Hz, 1H), 7.48 (s, 1H), 7.16 (d, *J* = 6.2 Hz, 1H), 6.90 (d, *J* = 3.5 Hz, 1H), 6.32 (dd, *J* = 6.8, 4.5 Hz, 1H), 4.51 – 4.44 (m, 1H), 4.21 (ddd, *J* = 11.4, 5.7, 3.1 Hz, 1H), 4.12 – 4.03 (m, 1H), 3.21 (q, *J* = 7.3 Hz, 2H, Et_3NH^+), 3.1 – 3.05 (m, 2H, Et_3NH^+), 2.57 (dq, *J* = 15.3, 8.2 Hz, 1H), 2.42 (ddt, *J* = 13.5, 9.3, 4.9 Hz, 1H), 2.33 – 2.17 (m, 2H), 1.31 – 1.25 (m, 6H, Et_3NH^+), 1.02 – 0.95 (m, 2H Et_3NH^+).

^{13}C NMR (126 MHz, D$_2$O): *δ* = 149.1, 139.7, 126.9, 126.2, 110.1, 103.3, 99.4, 86.2, 79.8 (d), 67.3 (d), 46.6 (Et_3NH^+), 31.4, 25.6, 10.4 (Et_3NH^+), 8.2 (Et_3NH^+).

^{31}P NMR (162 MHz, D$_2$O): *δ* = –10.3 (d, *J* = 19.6 Hz), –11.1 (d, *J* = 19.6 Hz), –23.1 (t, *J* = 19.7 Hz).

HR-MS (ESI): calcd for $(C_{12}H_{17}N_3O_{11}P_3)^-$ $[M–H]^-$: 472.0081, found: 472.0102.

Supplementary references

1. Fulmer, G. R. *et al.* NMR chemical shifts of trace impurities: Common laboratory solvents, organics, and gases in deuterated solvents relevant to the organometallic chemist. *Organometallics* **29**, 2176–2179 (2010).
2. Seela, F. & Bourgeois, W. Synthesis of 3,7-Dideaza-2'-deoxyadenosine and Related Pyrrolo[3,2-c]pyridine 2'-deoxyribo- and 2',3'-Dideoxyribonucleosides. *Synthesis* **12**, 938–943 (1988).
3. McBride, L. J., Kierzek, R., Beaucage, S. L. & Caruthers, M. H. Amidine Protecting Groups for Oligonucleotide Synthesis. *J. Am. Chem. Soc.* **108**, 2040–2048 (1986).
4. Saneyoshi, H., Michel, B. Y., Choi, Y., Strazewski, P. & Marquez, V. E. Synthesis of conformationally locked versions of puromycin analogues. *J. Org. Chem.* **73**, 9435–9438 (2008).
5. Riml, C., Lusser, A., Ennifar, E. & Micura, R. Synthesis, Thermodynamic Properties, and Crystal Structure of RNA Oligonucleotides Containing 5-Hydroxymethylcytosine. *J. Org. Chem.* **82**, 7939–7945 (2017).
6. Zhang, B., Bailey, V. C. & Potter, B. V. L. Chemoenzymatic synthesis of 7-deaza cyclic adenosine 5′-diphosphate ribose analogues, membrane-permeant modulators of intracellular calcium release. *J. Org. Chem.* **73**, 1693–1703 (2008).
7. Sun, Q. *et al.* Efficient synthesis of 5-hydroxymethyl-, 5-formyl-, and 5-carboxyl-2′-deoxycytidine and their triphosphates. *RSC Adv.* **4**, 36036–36039 (2014).

NMR spectra

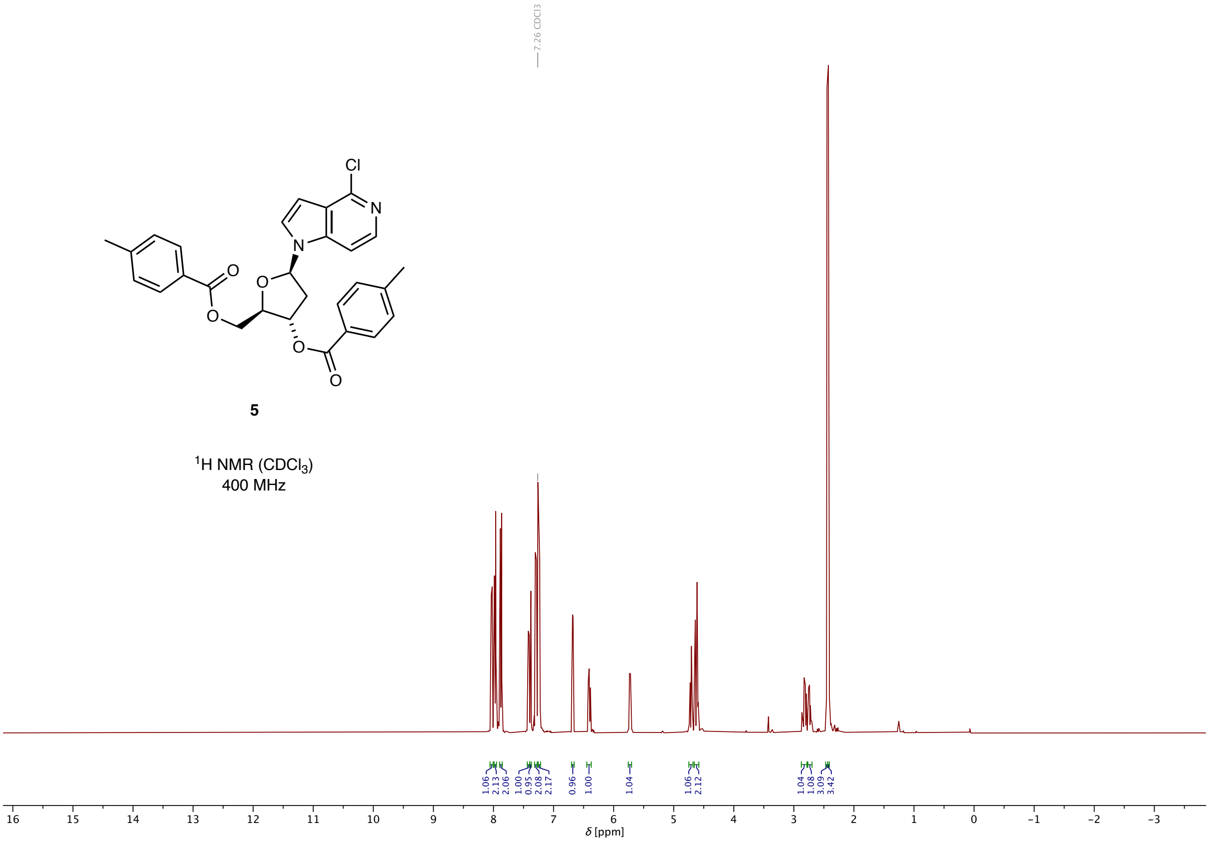
7.26 CDCl3
Cl
N
O
N
O
O
O
O
5
1H NMR (CDCl3)
400 MHz
1.06
2.13
2.06
1.00
0.95
2.08
2.17
0.96
1.00
1.04
1.06
2.12
1.04
1.08
3.09
3.42
16 15 14 13 12 11 10 9 8 7 6 5 4 3 2 1 0 −1 −2 −3
δ [ppm]

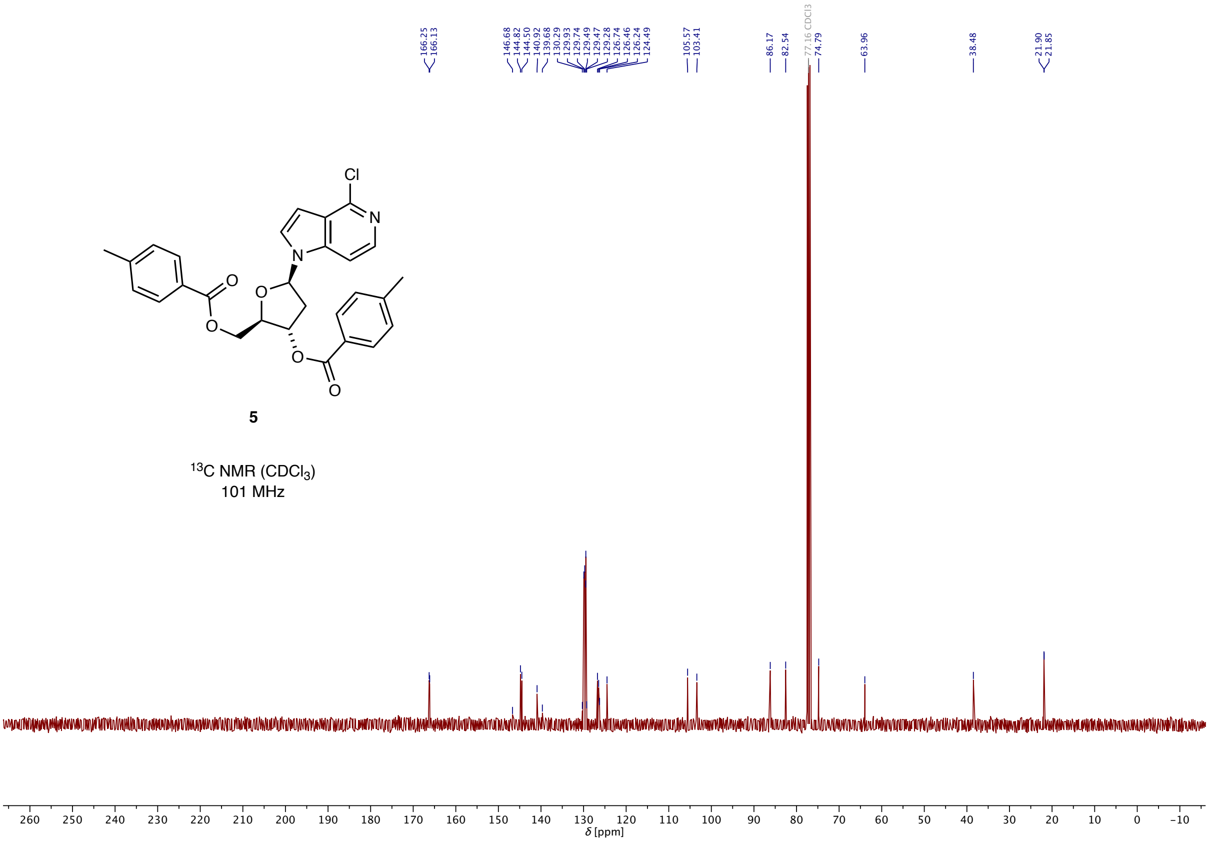
166.25
166.13
146.68
144.82
144.50
140.92
139.68
130.29
129.93
129.74
129.49
129.47
129.28
126.74
126.46
126.24
124.49
105.57
103.41
86.17
82.54
77.16 CDCl3
74.79
63.96
38.48
21.90
21.85
Cl
N
O
N
O
O
O
O
5
13C NMR (CDCl3)
101 MHz
260 250 240 230 220 210 200 190 180 170 160 150 140 130 120 110 100 90 80 70 60 50 40 30 20 10 0 −10
δ [ppm]

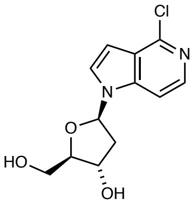

4

^{1}H NMR (DMSO-d_6)
400 MHz

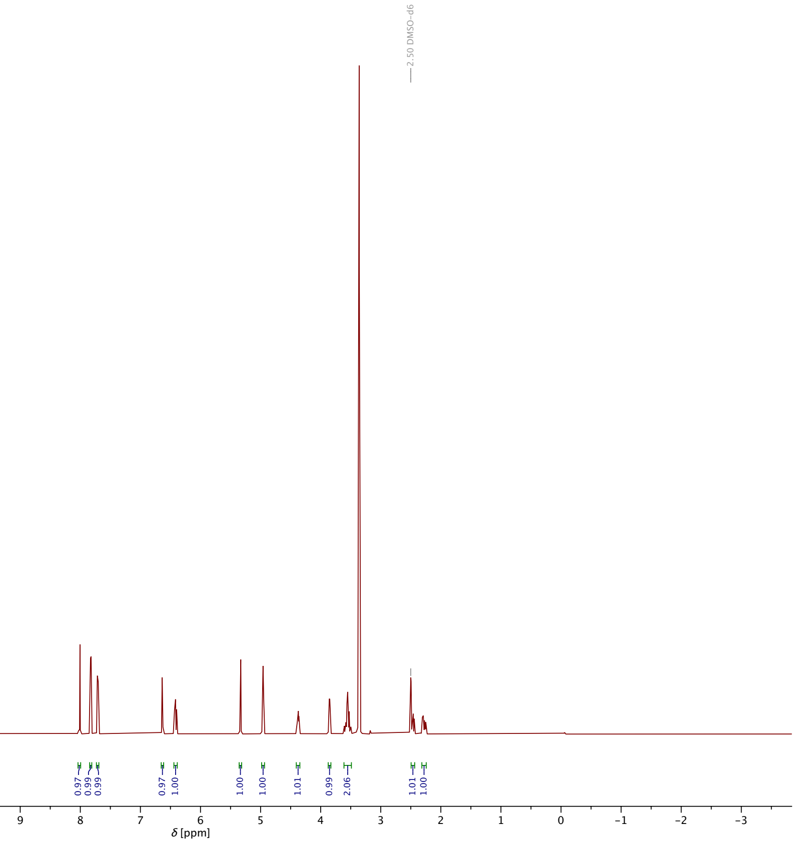

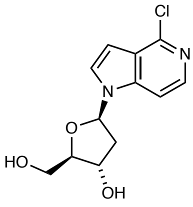

4

^{13}C NMR (DMSO-d_6)
101 MHz

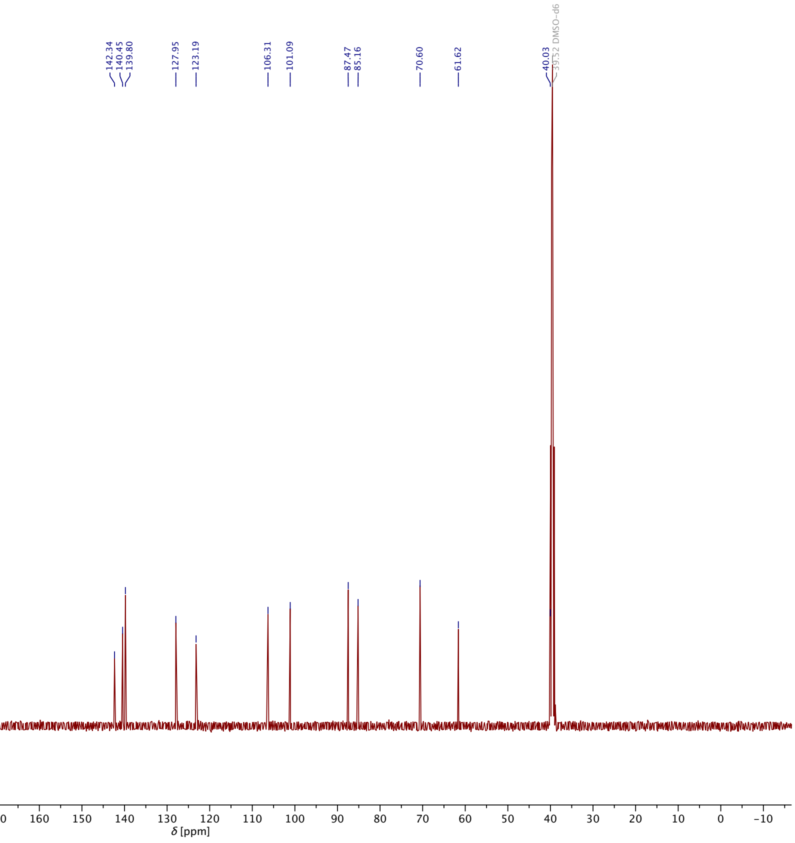

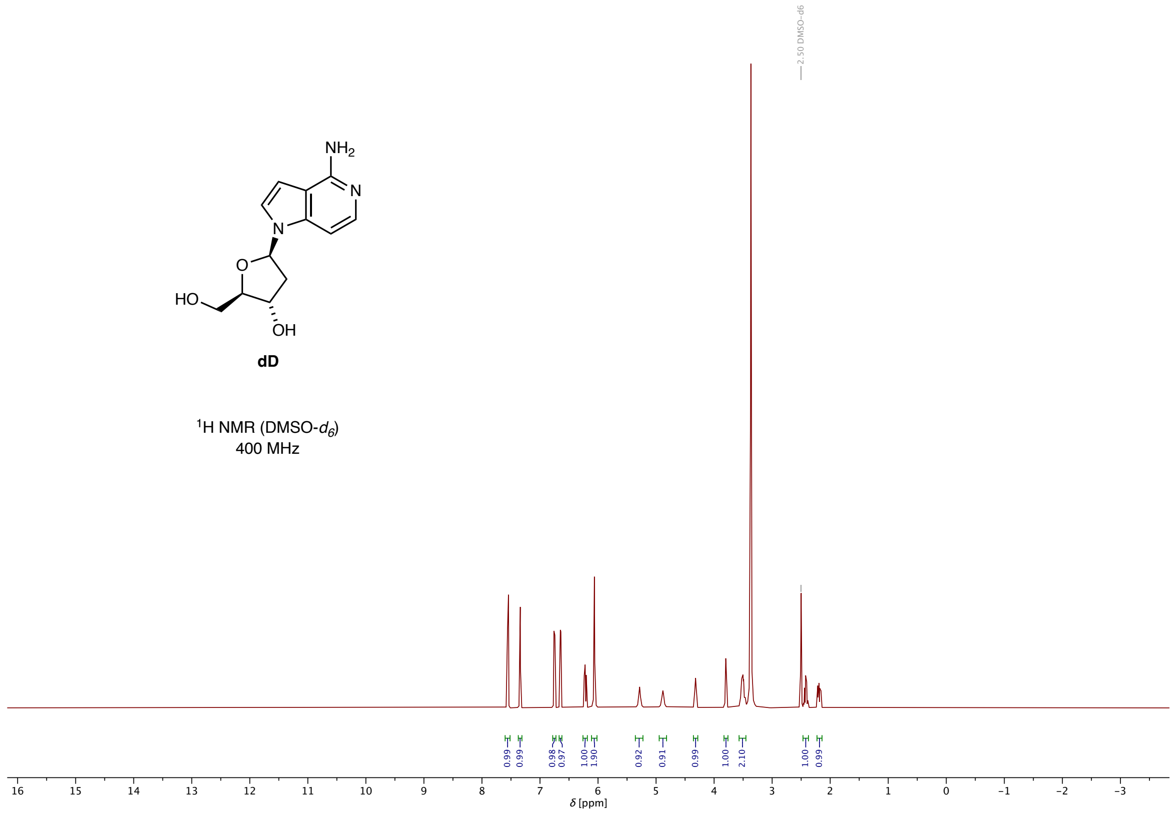
NH2
N
N
O
HO
OH
dD
1H NMR (DMSO-d6)
400 MHz
2.50 DMSO-d6
0.99
0.99
0.98
0.97
1.00
1.90
0.92
0.91
0.99
1.00
2.10
1.00
0.99
δ [ppm]

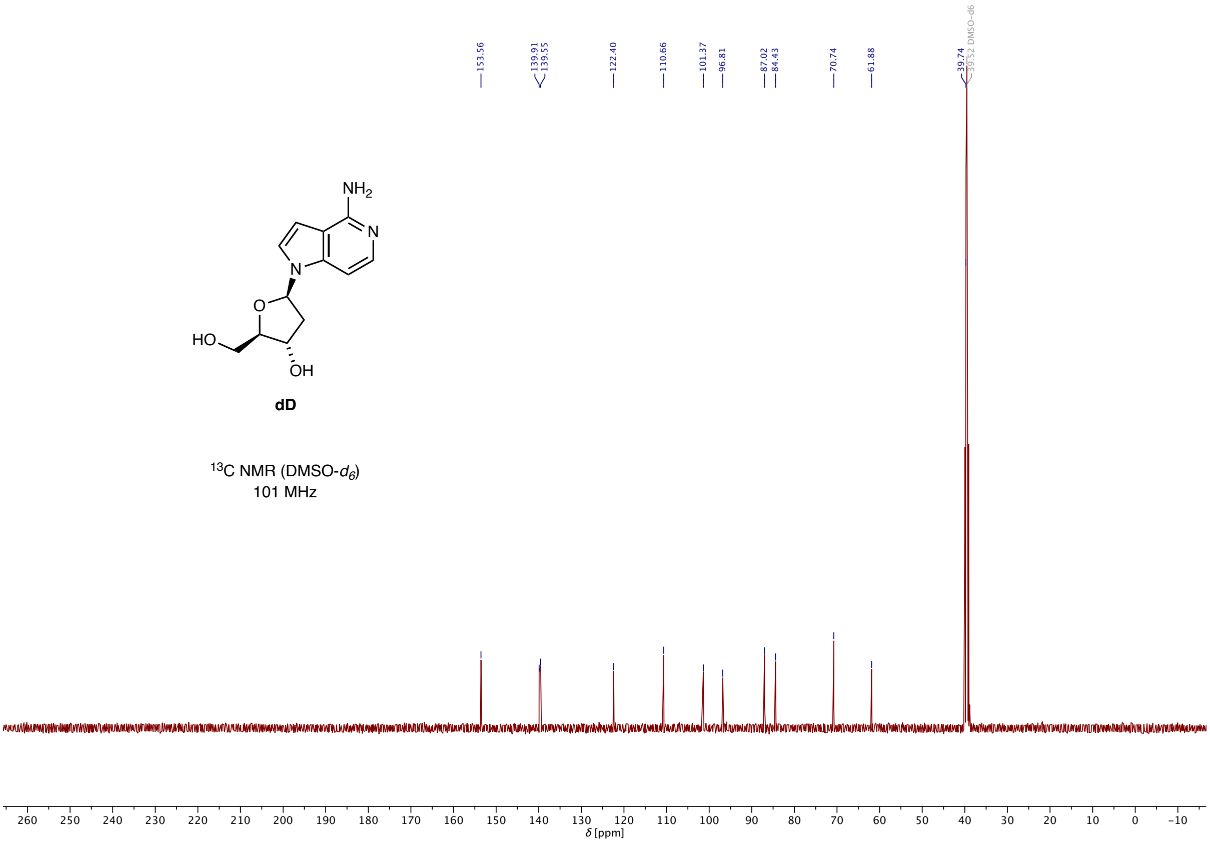
153.56
139.91
139.55
122.40
110.66
101.37
96.81
87.02
84.43
70.74
61.88
39.74
39.52 DMSO-d6
NH2
N
N
O
HO
OH
dD
13C NMR (DMSO-d6)
101 MHz
δ [ppm]

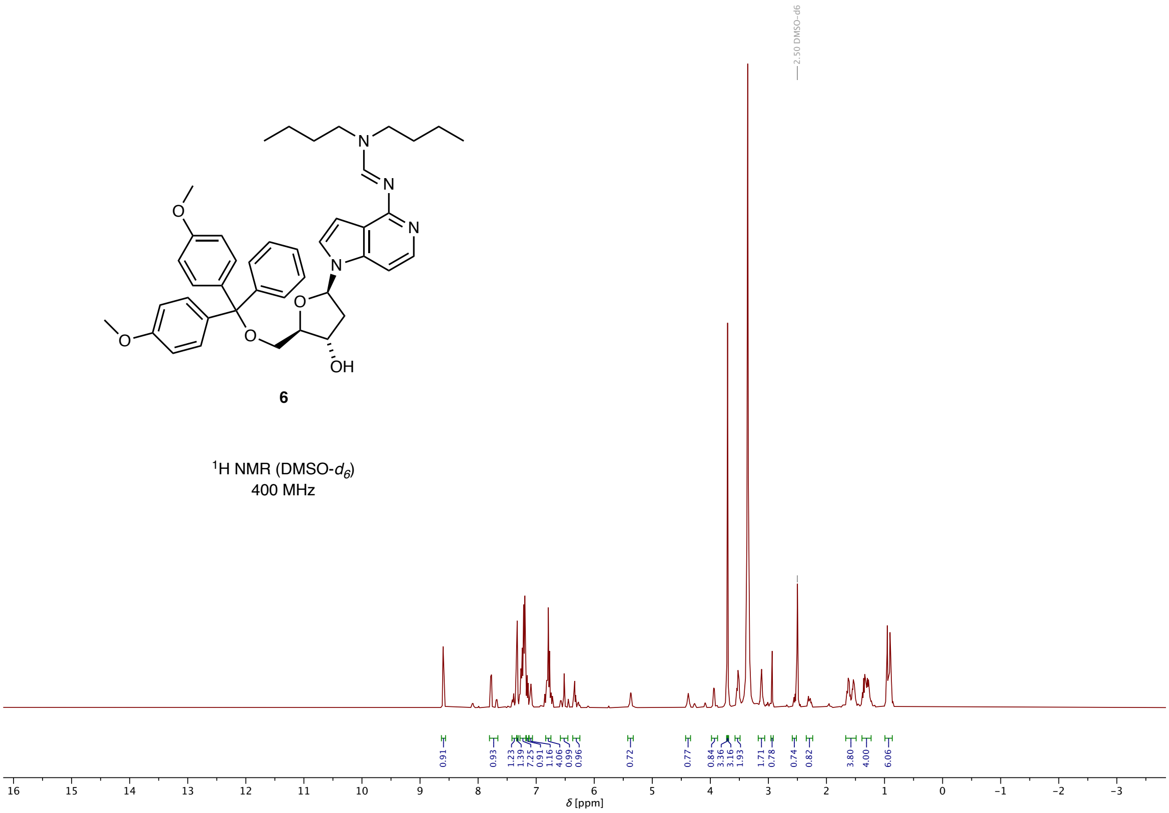

N
N
N
N
O
O
O
O
OH
6
¹H NMR (DMSO-d6)
400 MHz
2.50 DMSO-d6
δ [ppm]

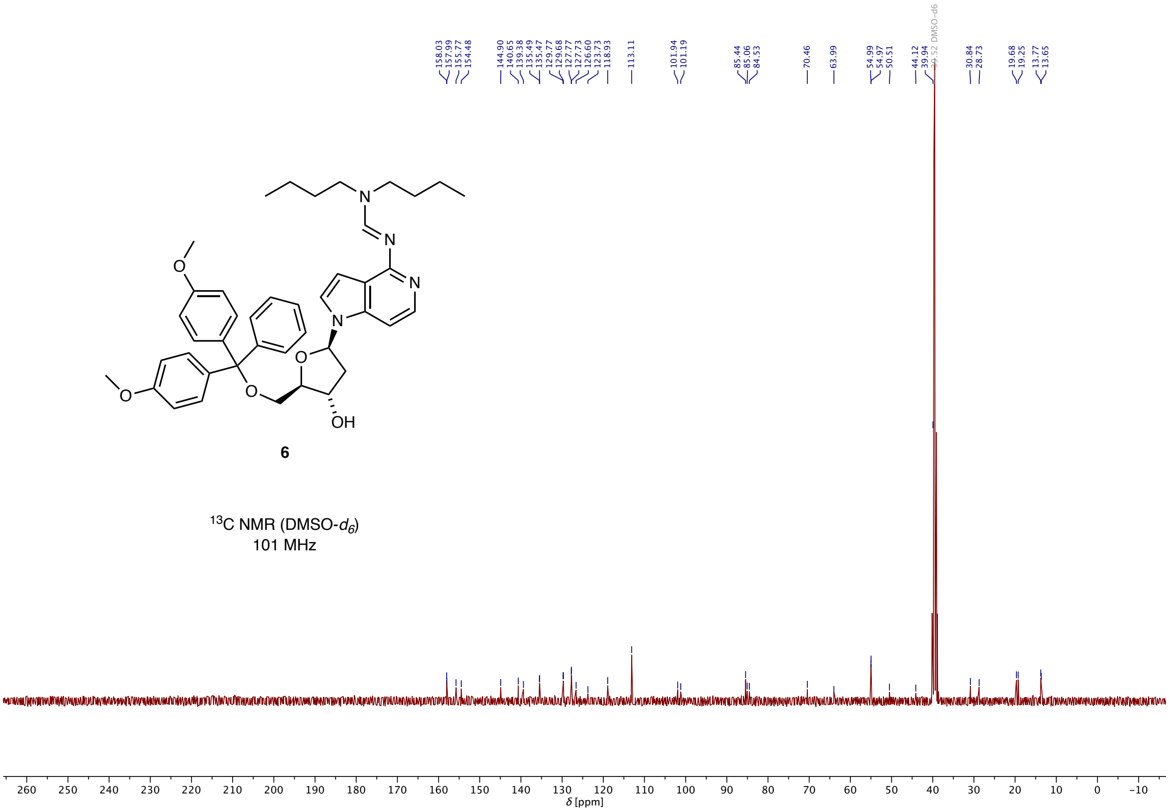

N
N
N
N
O
O
O
O
OH
6
¹³C NMR (DMSO-d6)
101 MHz
158.03
157.99
155.77
154.48
144.90
140.65
139.38
135.49
135.47
129.77
129.68
127.77
127.73
126.60
123.73
118.93
113.11
101.94
101.19
85.44
85.06
84.53
70.46
63.99
54.99
54.97
50.51
44.12
39.94
39.52 DMSO-d6
30.84
28.73
19.68
19.25
13.77
13.65
δ [ppm]

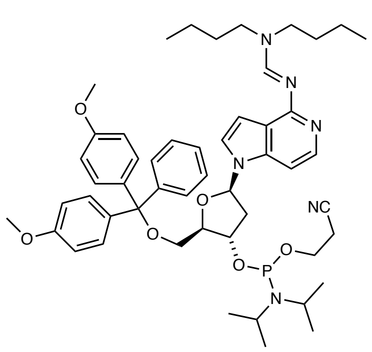

1

^{1}H NMR ($CDCl_3$)
500 MHz

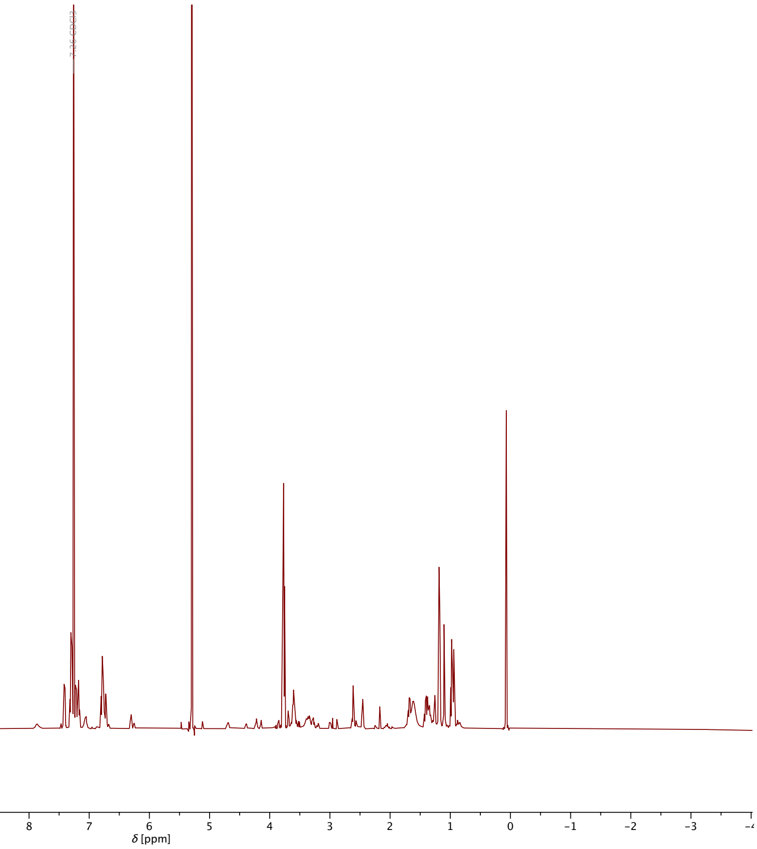

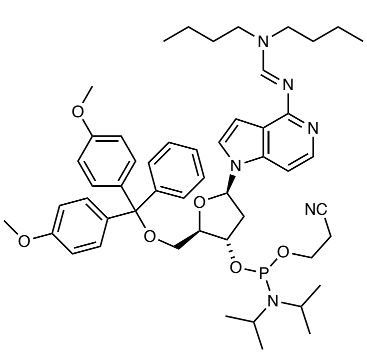

1

^{13}C NMR ($CDCl_3$)
126 MHz

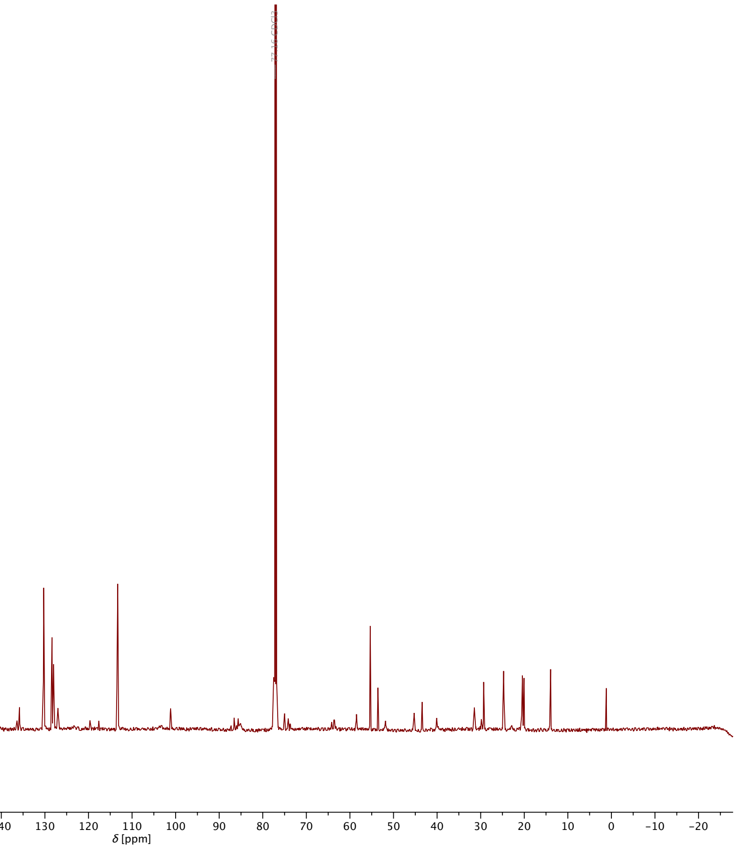

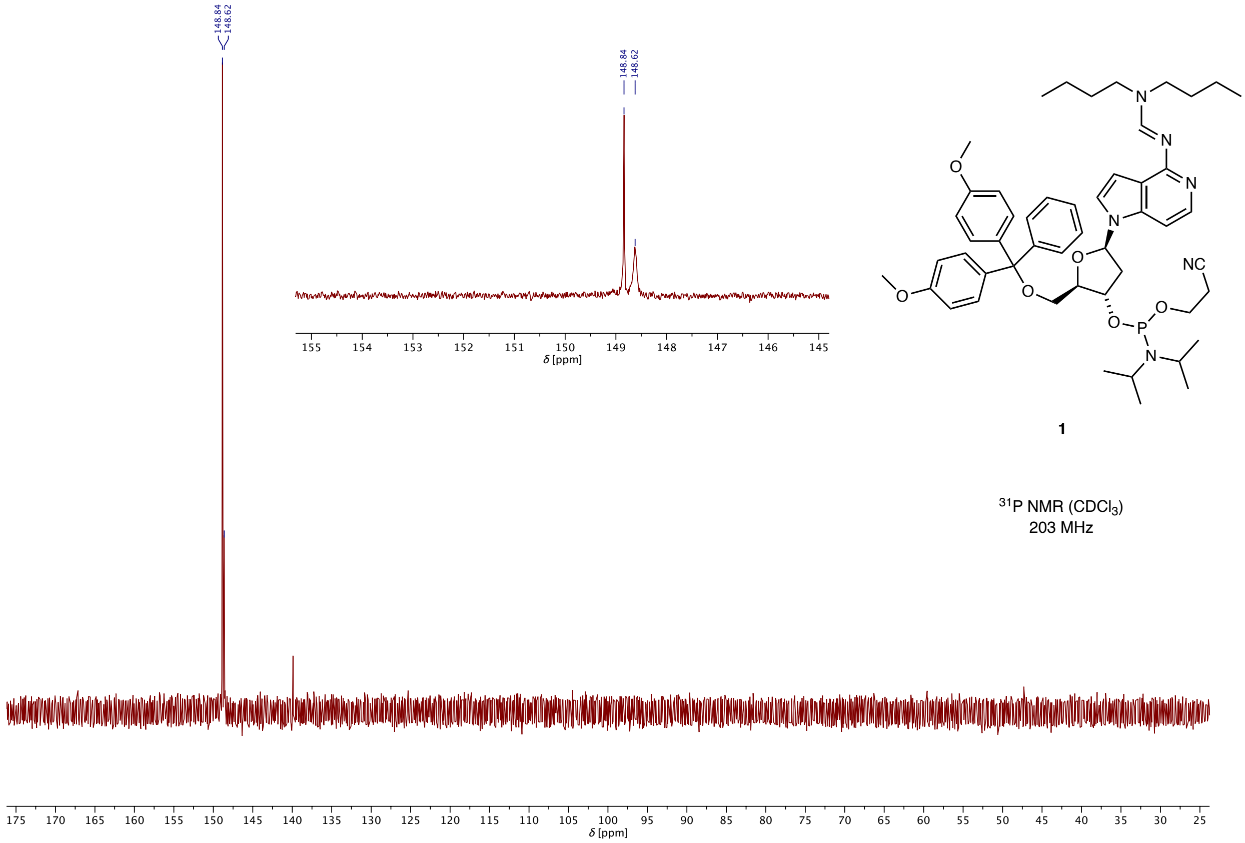
148.84
148.62
δ [ppm]
NC
O
P
N
1
31P NMR (CDCl3)
203 MHz

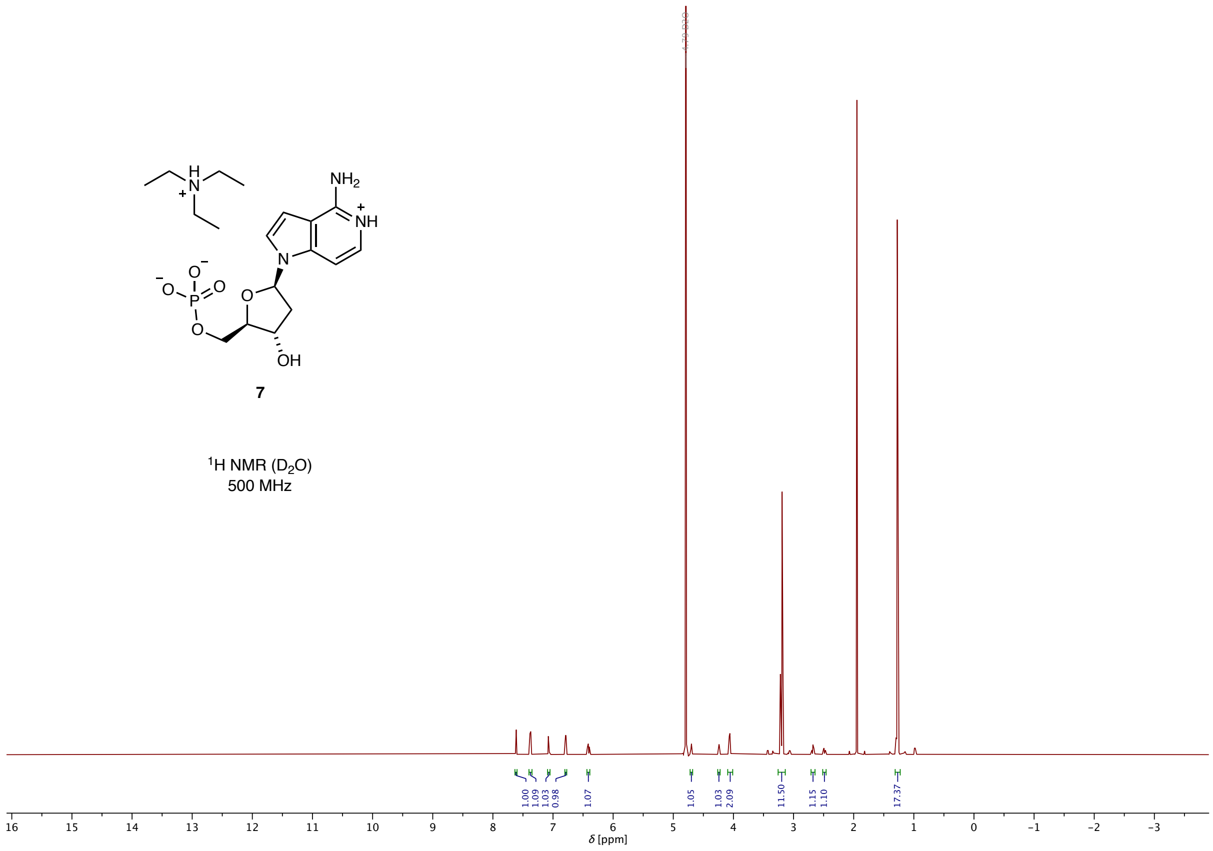

NH2
NH
N
O
P
O
OH
7
1H NMR (D2O)
500 MHz
1.00
1.09
1.03
0.98
1.07
1.05
1.03
2.09
11.50
1.15
1.10
17.37
δ [ppm]

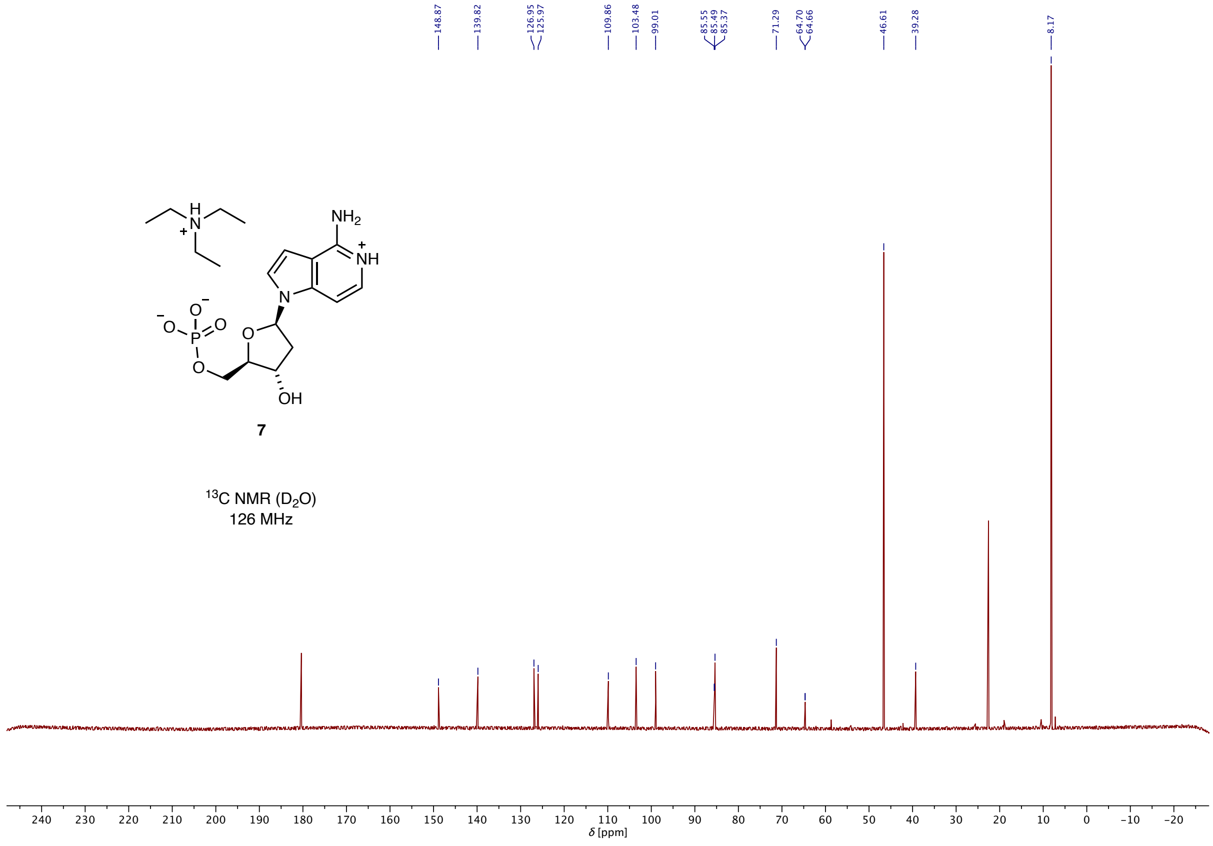

148.87
139.82
126.95
125.97
109.86
103.48
99.01
85.55
85.49
85.37
71.29
64.70
64.66
46.61
39.28
8.17
NH2
NH
N
O
P
O
OH
7
13C NMR (D2O)
126 MHz
δ [ppm]

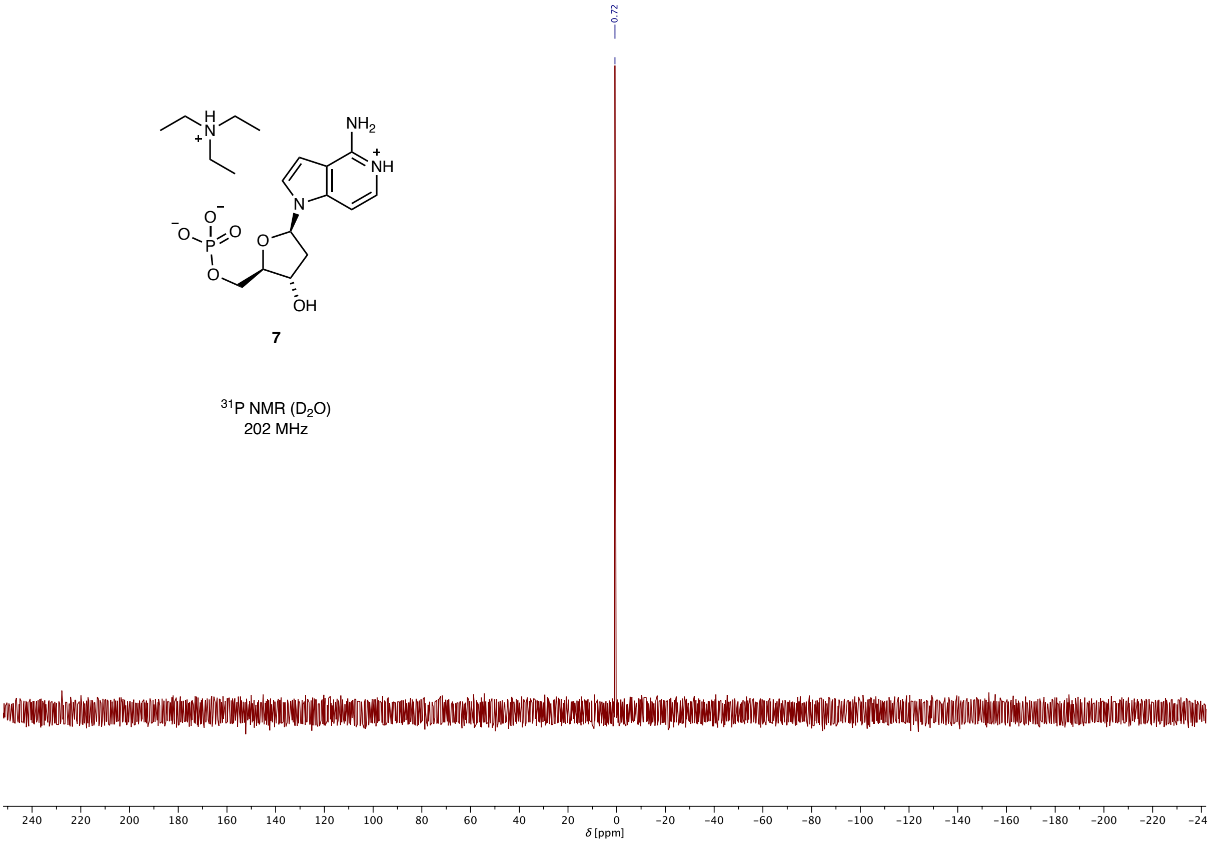
NH2
NH
N
O
P
OH
7
31P NMR (D2O)
202 MHz
0.72
δ [ppm]

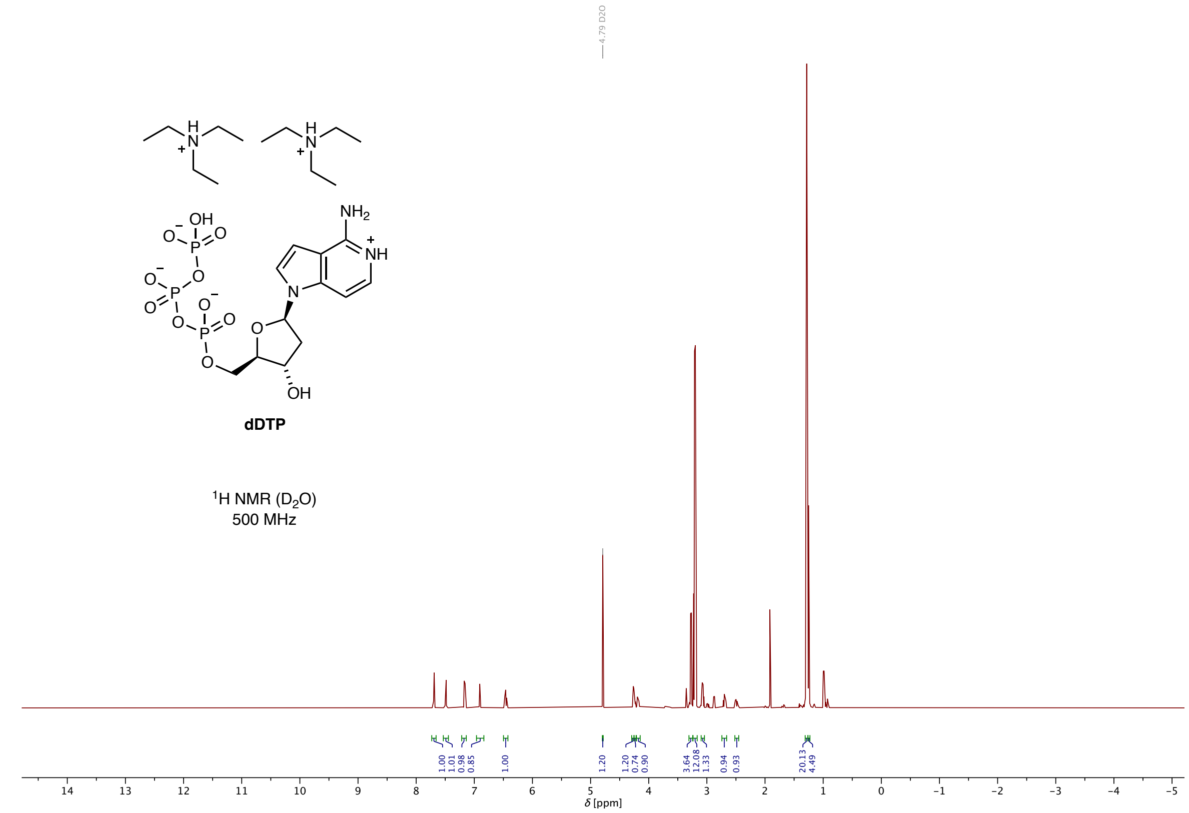
4.79 D2O
NH2
NH
OH
dDTP
1H NMR (D2O)
500 MHz
1.00 1.01 0.98 0.85 1.00 1.20 1.20 0.74 0.90 3.64 12.08 1.33 0.94 0.93 20.13 4.49
δ [ppm]

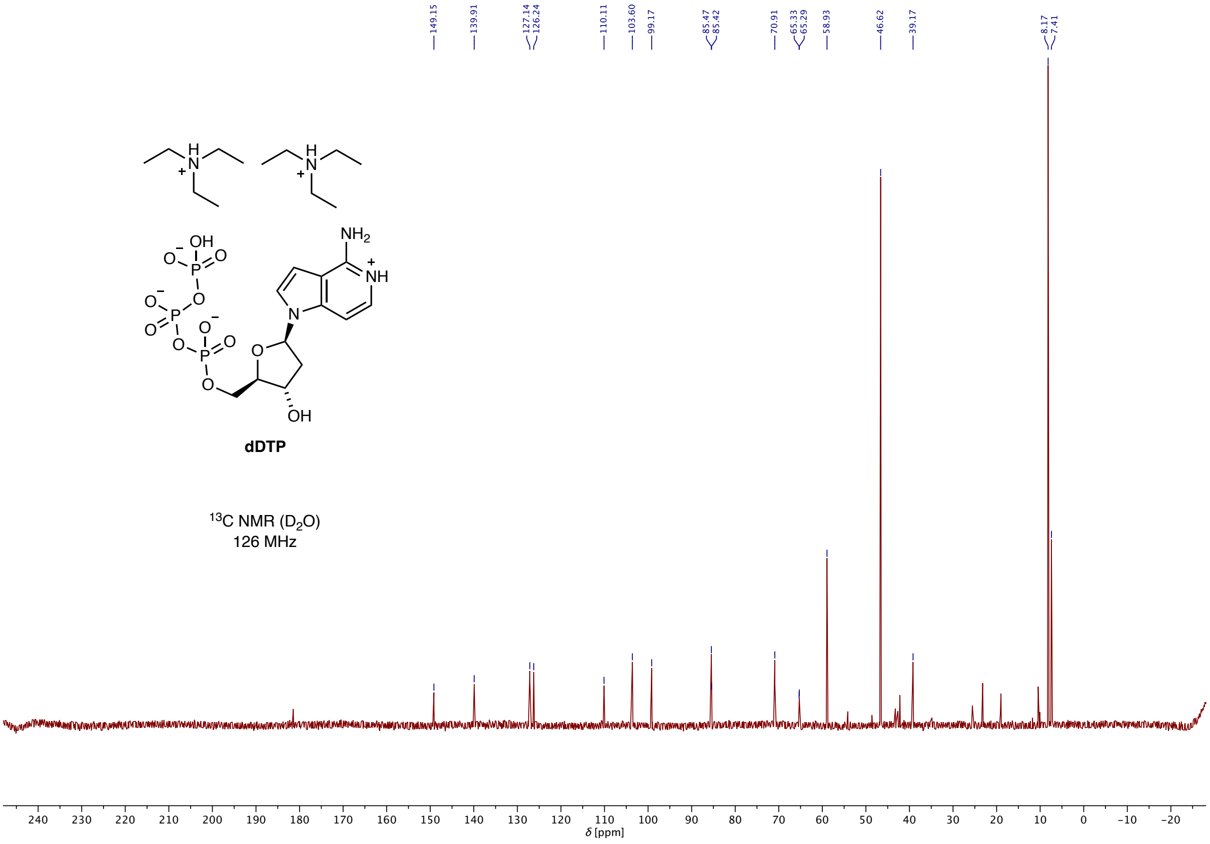
149.15
139.91
127.14
126.24
110.11
103.60
99.17
85.47
85.42
70.91
65.33
65.29
58.93
46.62
39.17
8.17
7.41
NH2
NH
OH
dDTP
13C NMR (D2O)
126 MHz
δ [ppm]

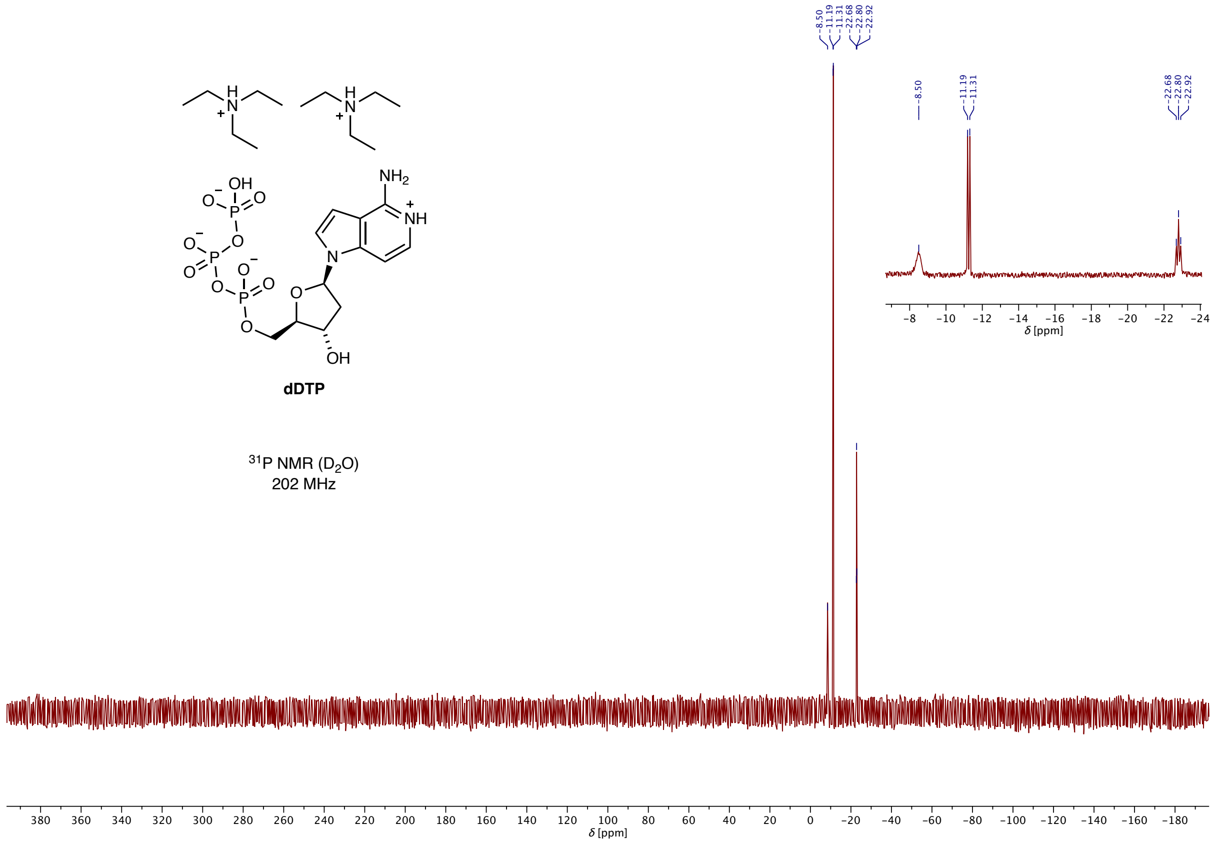

dDTP

^{31}P NMR (D_2O)
202 MHz

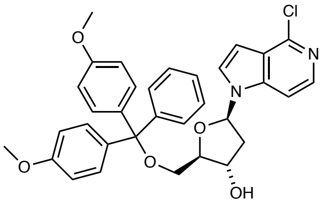

8

^{1}H NMR (DMSO-d_6)
700 MHz

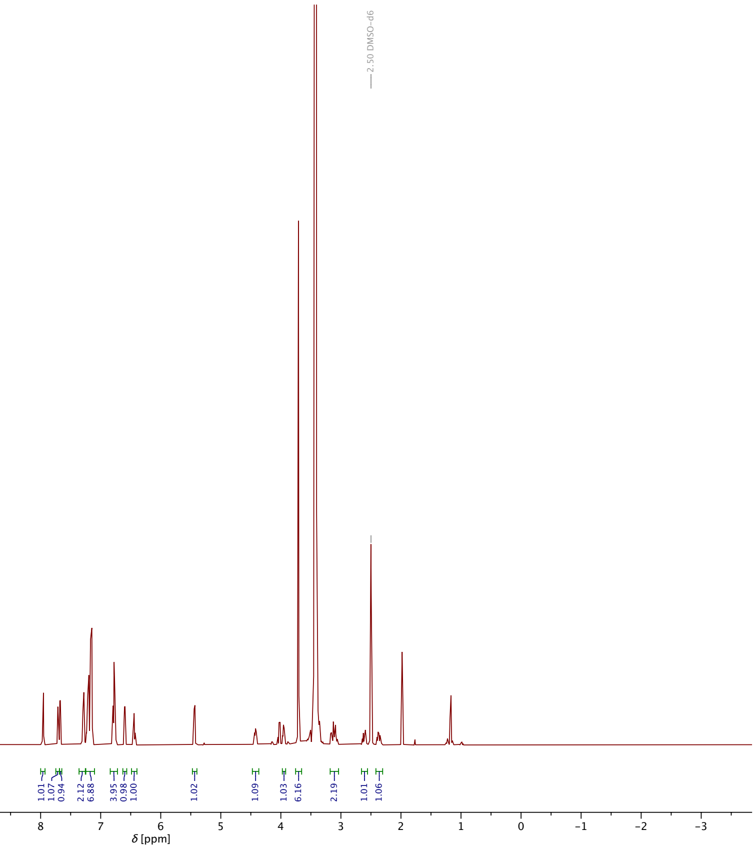

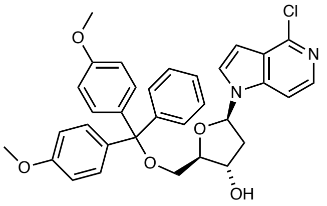

8

^{13}C NMR (DMSO-d_6)
176 MHz

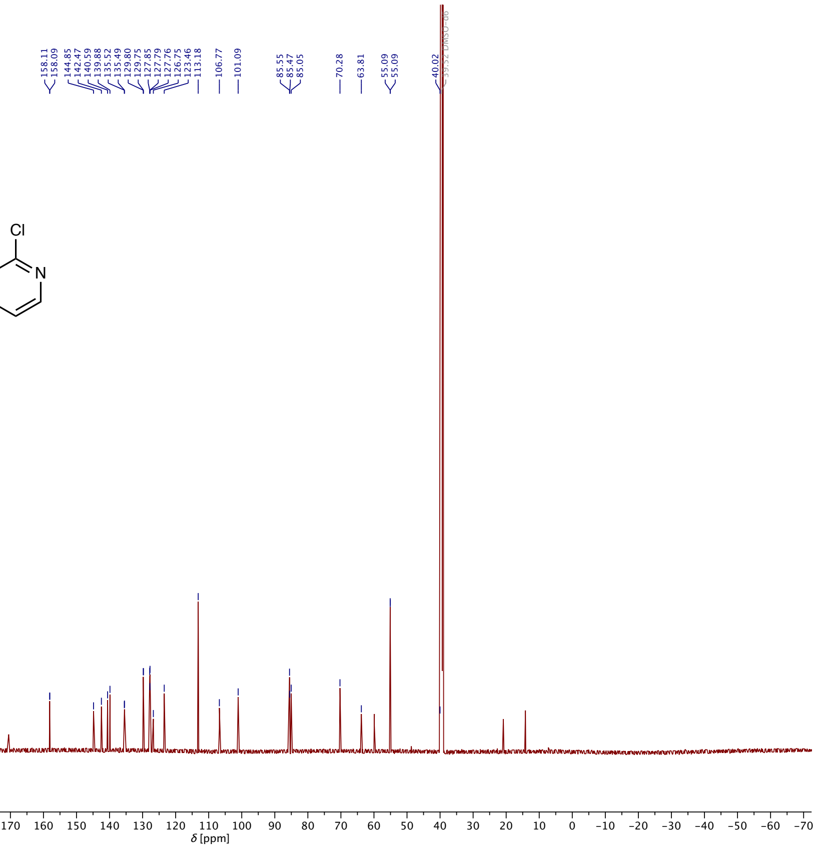

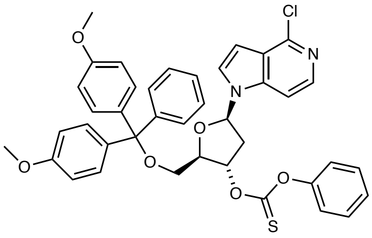

9

^{1}H NMR (DMSO-d_6)
700 MHz

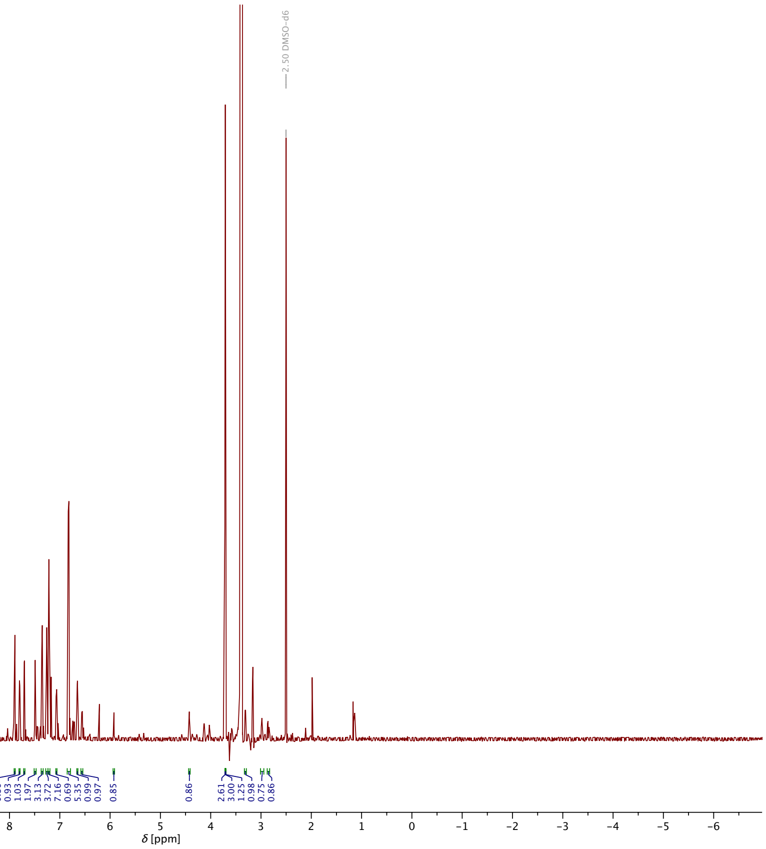

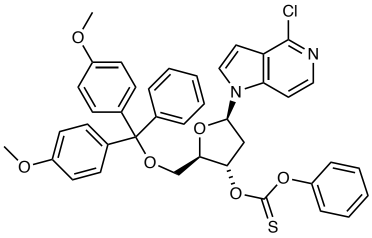

9

^{13}C NMR (DMSO-d_6)
176 MHz

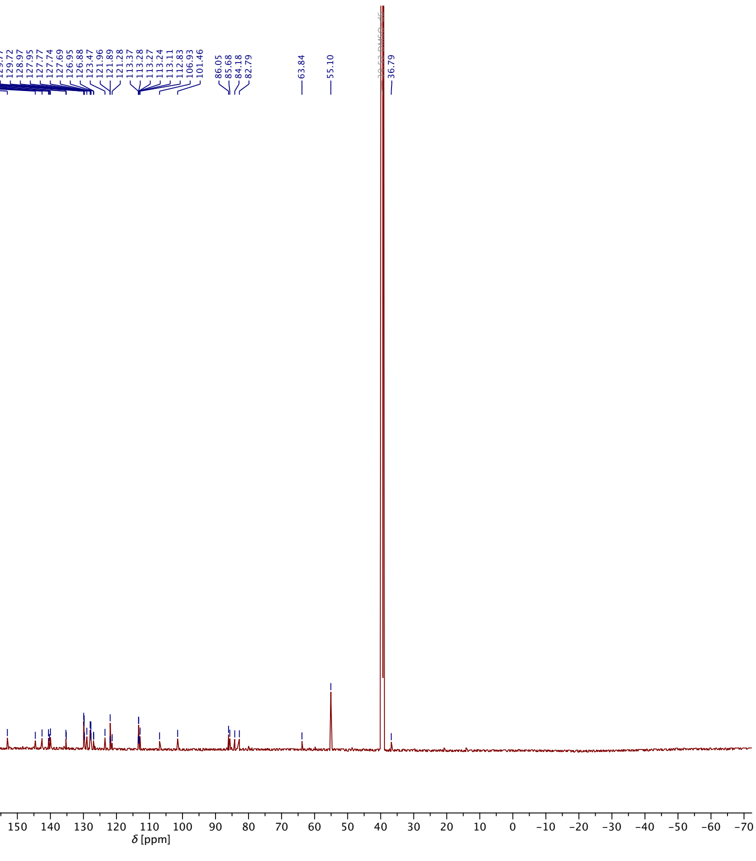

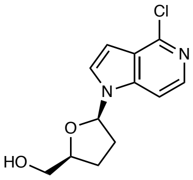

10

^{1}H NMR (DMSO-d_6)
400 MHz

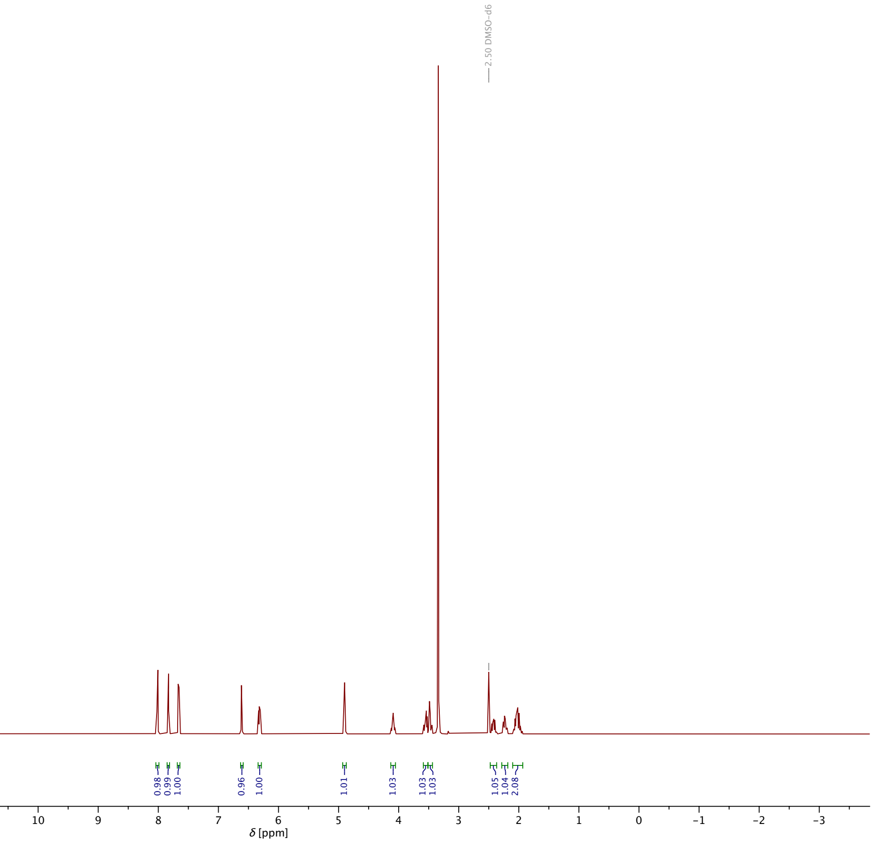

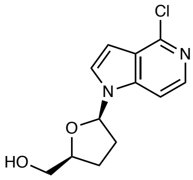

10

^{13}C NMR (DMSO-d_6)
101 MHz

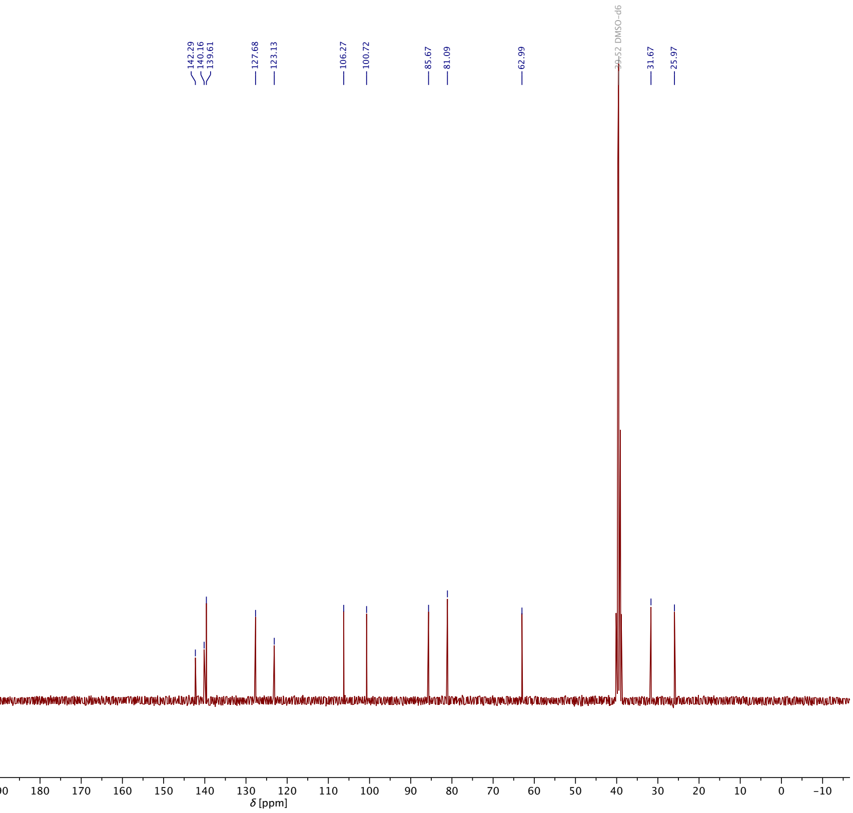

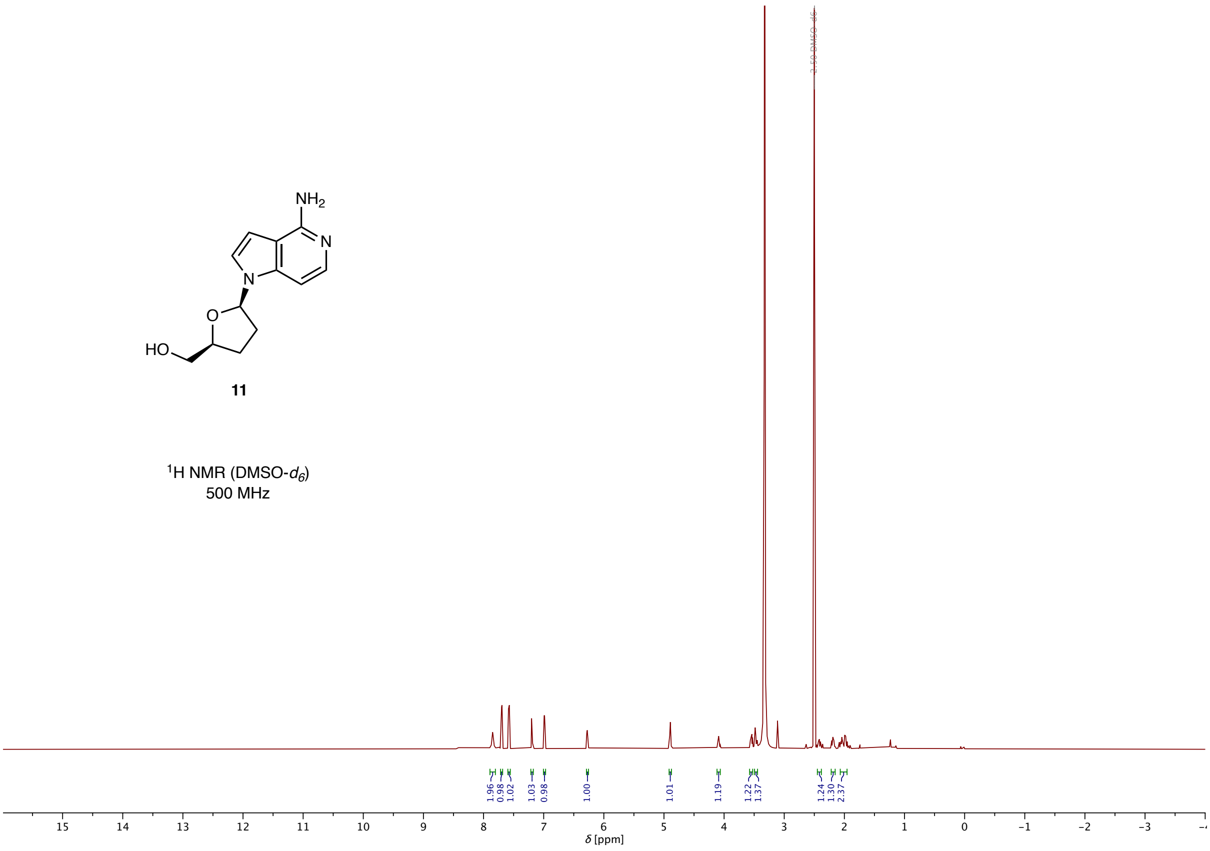
NH2
N
N
O
HO
11
1H NMR (DMSO-d6)
500 MHz
1.96
0.98
1.02
1.03
0.98
1.00
1.01
1.19
1.22
1.37
1.24
1.30
2.37
δ [ppm]

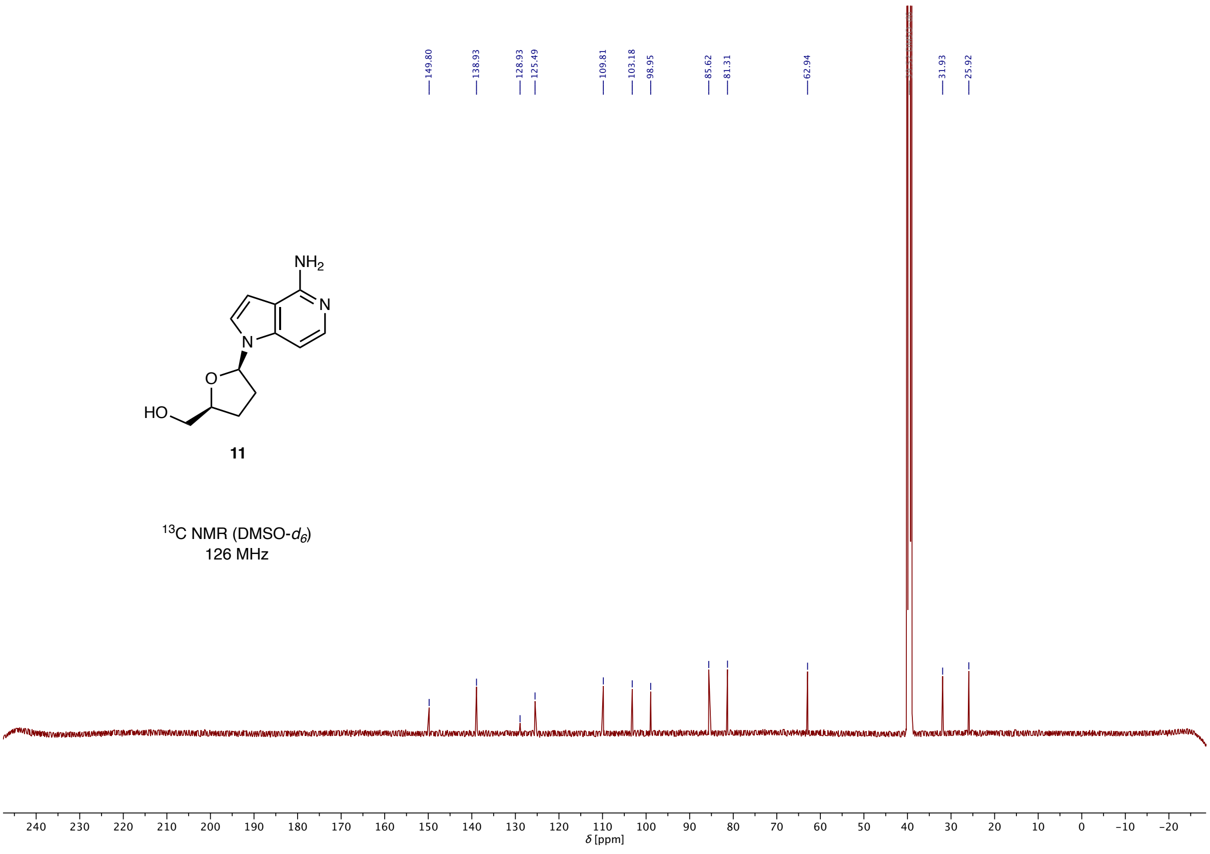
149.80
138.93
128.93
125.49
109.81
103.18
98.95
85.62
81.31
62.94
31.93
25.92
NH2
N
N
O
HO
11
13C NMR (DMSO-d6)
126 MHz
δ [ppm]

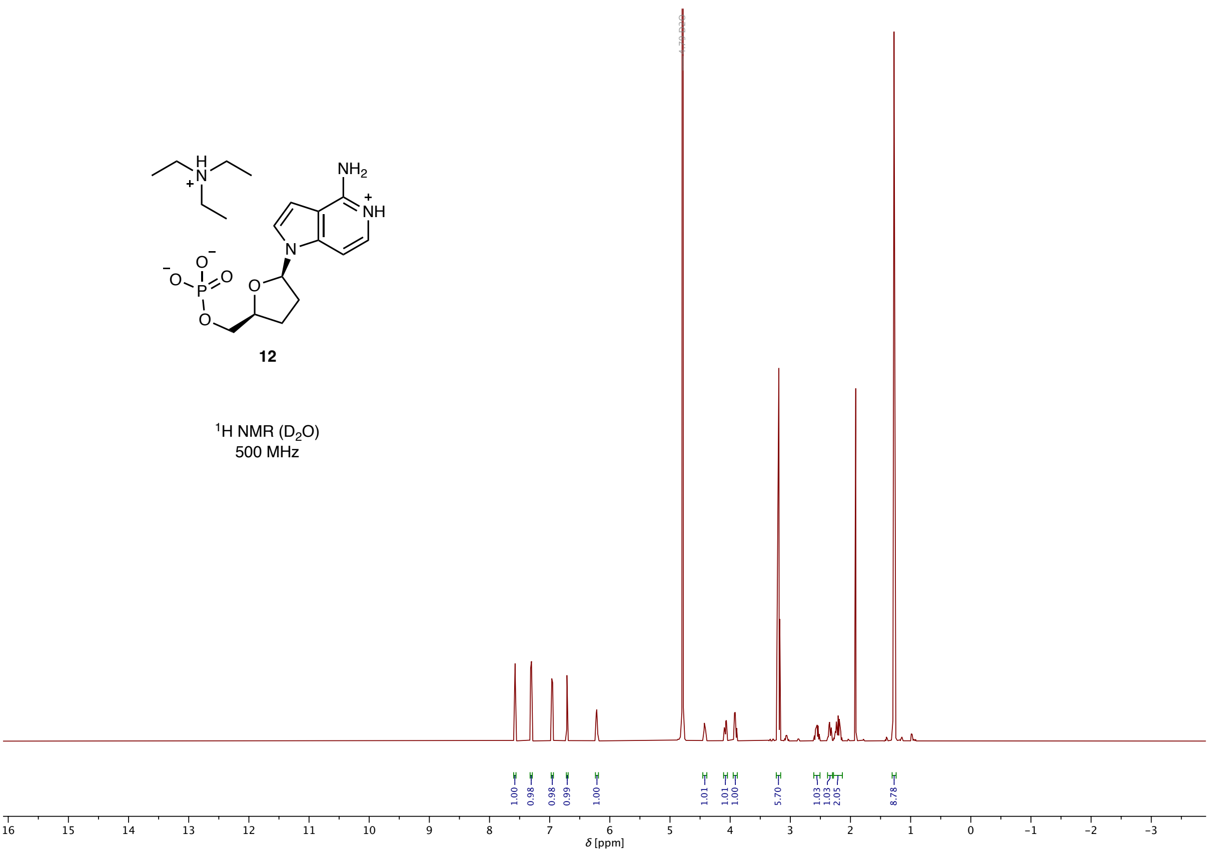
12
1H NMR (D2O)
500 MHz
1.00
0.98
0.98
0.99
1.00
1.01
1.01
1.00
5.70
1.03
1.03
2.05
8.78
δ [ppm]

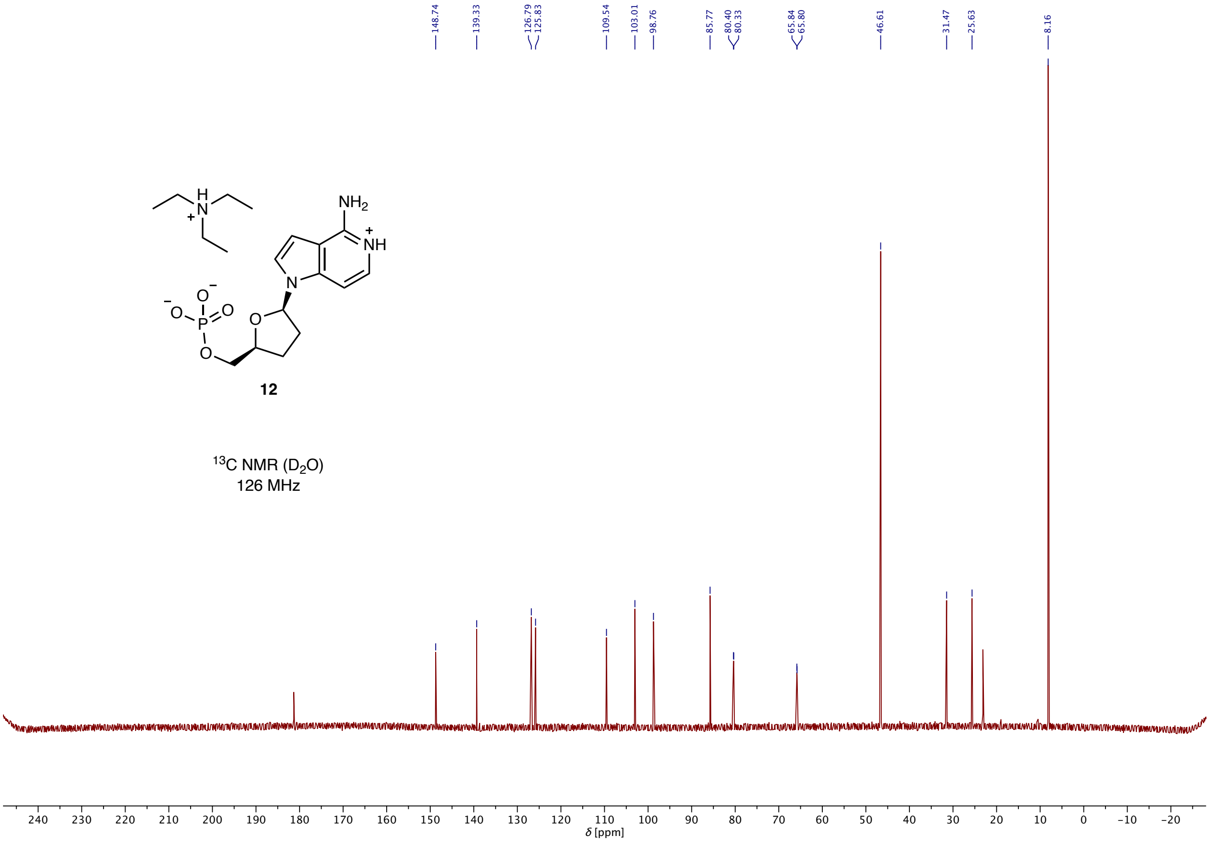
148.74
139.33
126.79
125.83
109.54
103.01
98.76
85.77
80.40
80.33
65.84
65.80
46.61
31.47
25.63
8.16
12
13C NMR (D2O)
126 MHz
δ [ppm]

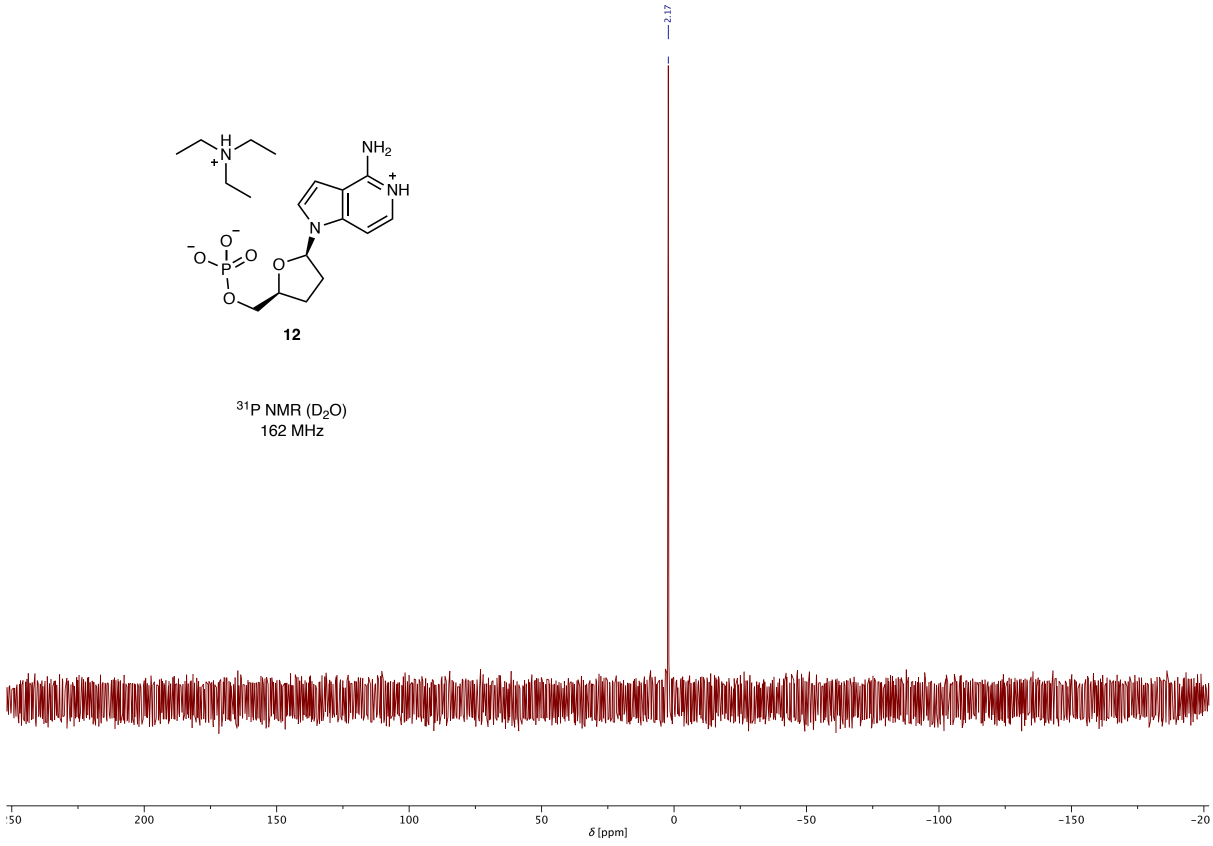

12

^{31}P NMR (D_2O)
162 MHz

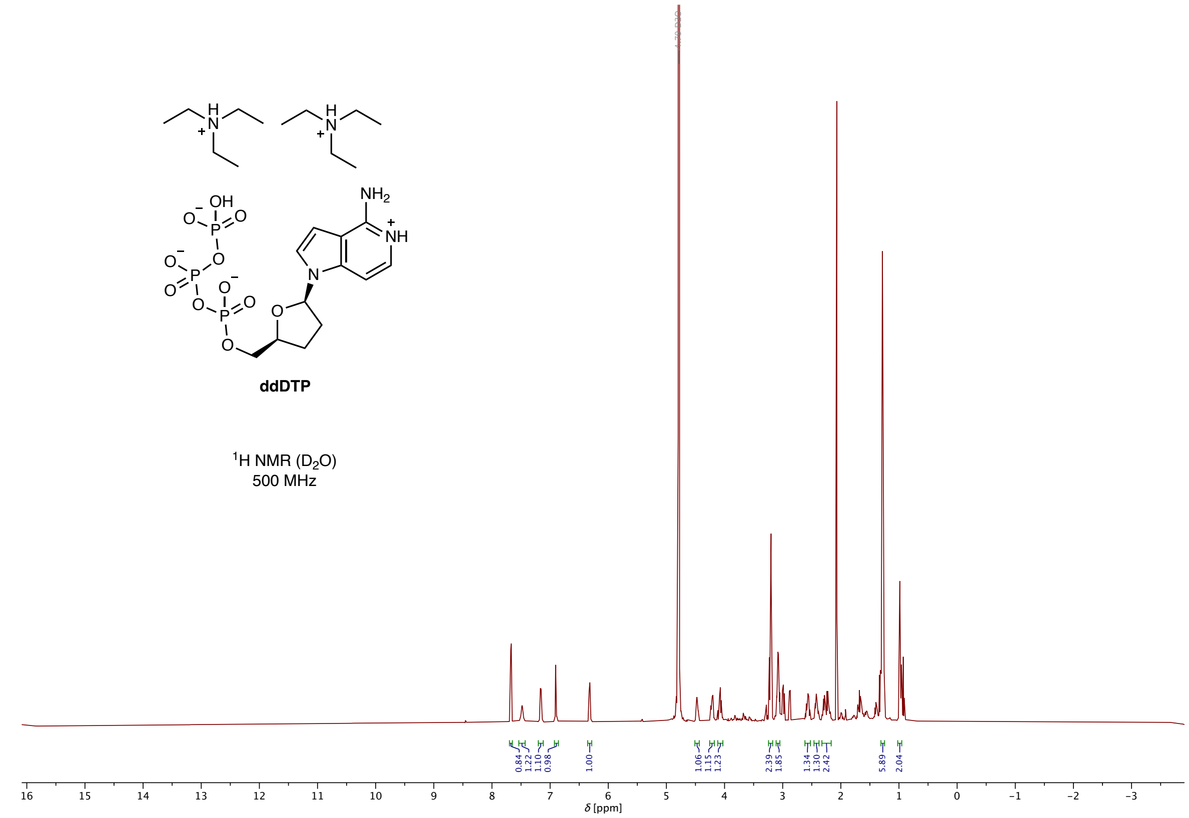
ddDTP
1H NMR (D2O)
500 MHz
0.84
1.22
1.10
0.98
1.00
1.06
1.15
1.23
2.39
1.85
1.34
1.30
2.42
5.89
2.04
δ [ppm]

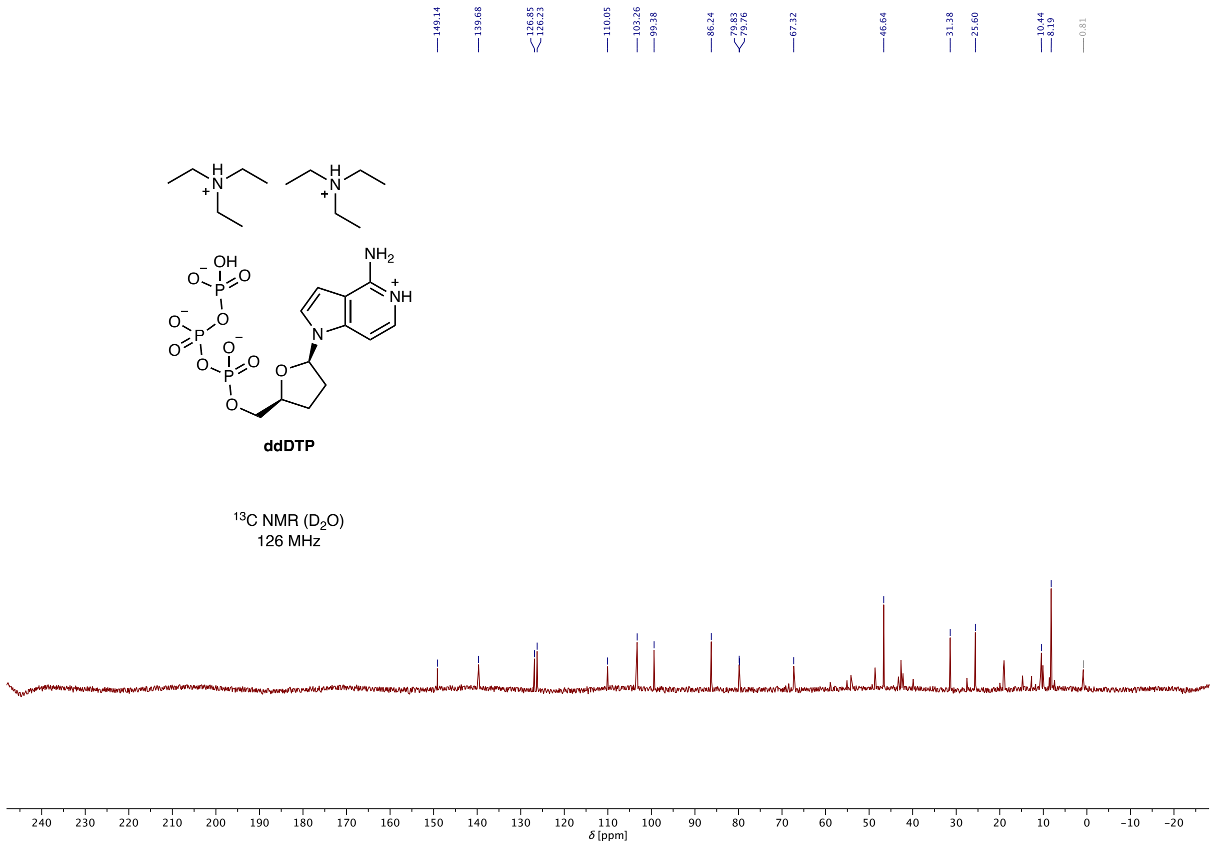
ddDTP
13C NMR (D2O)
126 MHz
149.14
139.68
126.85
126.23
110.05
103.26
99.38
86.24
79.83
79.76
67.32
46.64
31.38
25.60
10.44
8.19
0.81
δ [ppm]

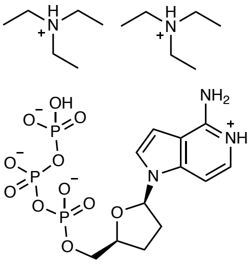

ddDTP

^{31}P NMR (D_2O)
162 MHz

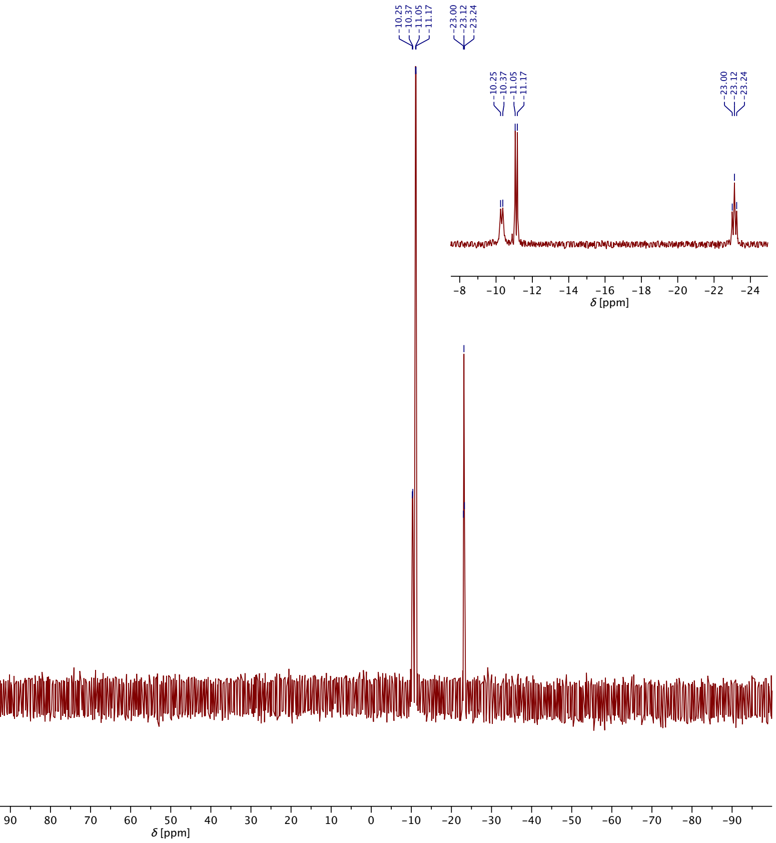

Supplementary Information Source Data

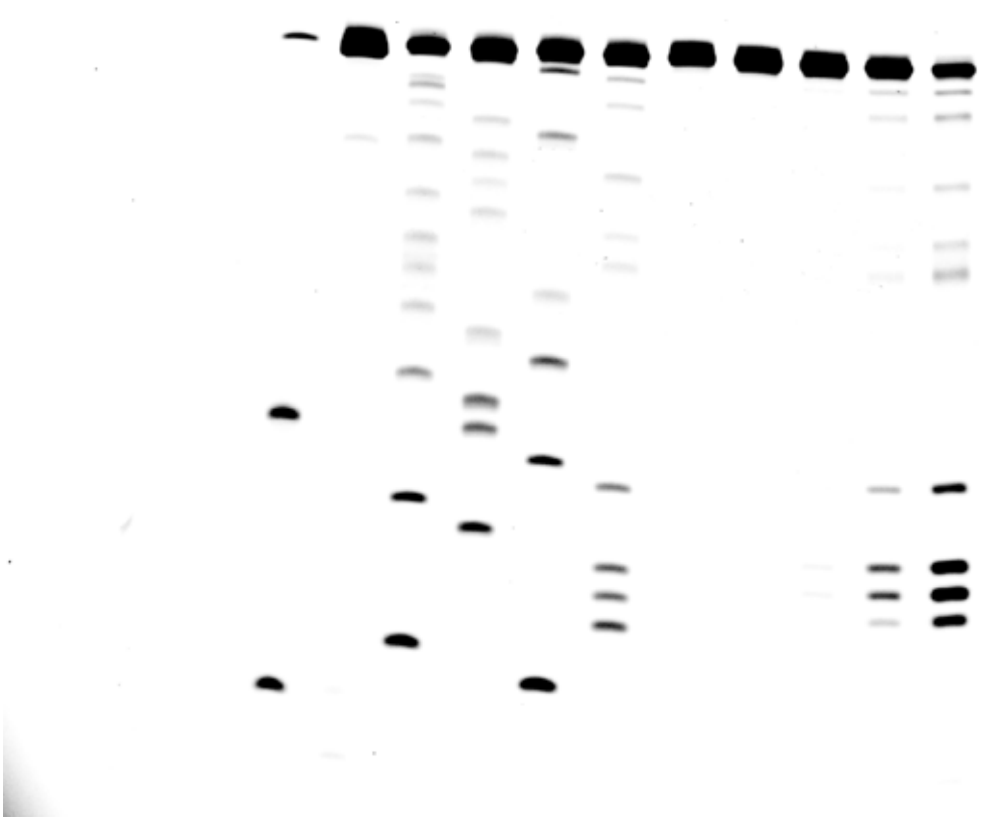

Source Data for Supplementary Fig. 19|Uncropped polyacrylamide gel.

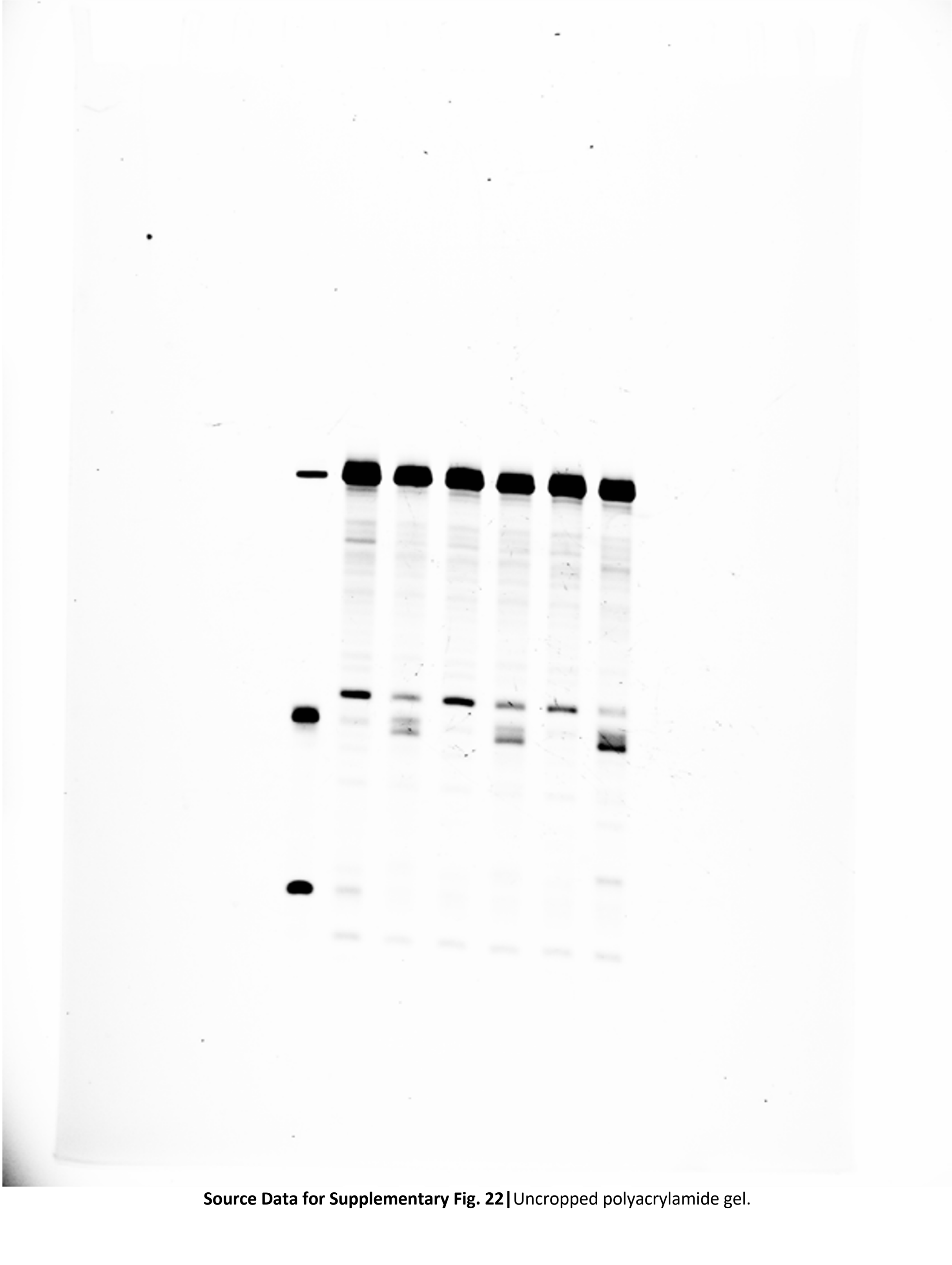

Source Data for Supplementary Fig. 22| Uncropped polyacrylamide gel.

R script for the processing of UV melting data

```
### Load packages required for script
library(tidyverse)
library(dplyr)
library(qPLEXanalyzer)
library(gridExtra)
library(gtools)
library(data.table)
library(ggplot2)
library(reshape2)
library(ggVennDiagram)
library("gplots")
library(ggcorrplot)
library(rstatix)
library(ggResidpanel)
library(plotly)
library(nlme)
library(MASS)
library(mgcv)
library(purrr)
library(stringr)
### Import all raw data files for each base pair and pH.
### Note the capacity of the UV-vis spectrometer meant two sets of triplicate
### values could be measured in a single run
files <- list.files(path="raw_data/",
                    pattern=".5.csv", full.names=TRUE)
ldf <- lapply(files, read.csv) # read csv files into a list of dataframes
names(ldf) <- str_sub(files, 11, -5) # name each dataframe for easier navigatio
n
ldf <- lapply(ldf, function(x) x[-c(1),]) # remove first row (field titles) fro
m each dataframe
ldf <- lapply(ldf, function(x) lapply(x, as.numeric)) # convert string values t
o numeric
ldf <- lapply(ldf, as.data.frame) # convert each double to a dataframe
ldf <- lapply(ldf, function(x) x[,-c(1,3,5,7,9,11,13,15,17,19,21,23)]) # remove
 repeated temperature value columns
abs_data <- lapply(ldf, function(x) x[,c(1:6)]) # separate absolute and derivat
ive absorbance values
deriv_data <- lapply(ldf, function(x) x[c(1:288),c(7:12)]) # separate absolute
and derivative absorbance values
abs_avg <- lapply(abs_data, function(x) data.frame(rowMeans(x[,1:3]), rowMeans(
x[,4:6])))
deriv_avg <- lapply(deriv_data, function(x) data.frame(rowMeans(x[,1:3]), rowMe
ans(x[,4:6])))
### Function to concatenate the separate runs at different pH into one df for a
 given base pair.
ph_concatenate <- function(input_list){
  count <- 1
  for (i in 1:length(input_list)){
    if (count == 1){
    }
    if (count == 2){
```

```
      input_list[[i-1]] <- cbind(input_list[[i-1]], input_list[[i]])
    }
    if (count == 3){
      input_list[[i-2]] <- cbind(input_list[[i-2]], input_list[[i]])
      count <- 0
    }
    count <- count + 1
  }
  del_vec <- c()
  for (i in 1:length(input_list)){
    if (ncol(input_list[[i]]) < ncol(input_list[[1]])){
      del_vec <- append(del_vec, i)
    }
  }
  input_list[del_vec] <- NULL
  names(input_list) <- str_sub(names(input_list), 1, -9)
  return(input_list)
}
### Apply this function to the absolute and derivative datasets
abs_data <- ph_concatenate(abs_data)
deriv_data <- ph_concatenate(deriv_data)
### set column titles as pH values
ph_names <- c("7.0","","", "7.5","","", "8.0","","", "8.5","","", "9.0","","",
"9.5","","")
abs_data <- lapply(abs_data, setNames, nm = ph_names)
deriv_data <- lapply(deriv_data, setNames, nm = ph_names)

### add temperature column
abs_temps <- data.frame("Temperature" = seq(20.0, 79.8, by = 0.2))
deriv_temps <- data.frame("Temperature" = seq(21.2, 78.6, by = 0.2))
abs_data <- lapply(abs_data, function(x) cbind(abs_temps, x))
deriv_data <- lapply(deriv_data, function(x) cbind(deriv_temps, x))
abs_avg <- ph_concatenate(abs_avg)
deriv_avg <- ph_concatenate(deriv_avg)
ph_names <- c("7.0", "7.5", "8.0", "8.5", "9.0", "9.5")
abs_avg <- lapply(abs_avg, setNames, nm = ph_names)
deriv_avg <- lapply(deriv_avg, setNames, nm = ph_names)
abs_avg <- lapply(abs_avg, function(x) cbind(abs_temps, x))
deriv_avg <- lapply(deriv_avg, function(x) cbind(deriv_temps, x))
ssDNA_abs_long <- read_csv("raw_data/ssDNA new abs.csv")
ssDNA_deriv_long <- read_csv("raw_data/ssDNA new deriv.csv")
#ssDNA_abs_long <- ssDNA_abs_long[,-c(4,5)]
#ssDNA_deriv_long <- ssDNA_deriv_long[,-c(4,5)]
ssDNA_abs <- data.frame("Temperature" = 0,
                        "7.0" = ssDNA_abs_long$r1[1:300],
                        ssDNA_abs_long$r1[1:300],
                        ssDNA_abs_long$r1[1:300],
                        "7.5" = ssDNA_abs_long$r1[301:600],
                        ssDNA_abs_long$r1[301:600],
                        ssDNA_abs_long$r1[301:600],
                        "8.0" = ssDNA_abs_long$r1[601:900],
                        ssDNA_abs_long$r1[601:900],
                        ssDNA_abs_long$r1[601:900],
                        "8.5" = ssDNA_abs_long$r1[901:1200],
```

```
                         ssDNA_abs_long$r1[901:1200],
                         ssDNA_abs_long$r1[901:1200],
                         "9.0" = ssDNA_abs_long$r1[1201:1500],
                         ssDNA_abs_long$r1[1201:1500],
                         ssDNA_abs_long$r1[1201:1500],
                         "9.5" = ssDNA_abs_long$r1[1501:1800],
                         ssDNA_abs_long$r1[1501:1800],
                         ssDNA_abs_long$r1[1501:1800])
ssDNA_deriv <- data.frame("Temperature" = 0,
                         "7.0" = ssDNA_deriv_long$r1[1:288],
                         ssDNA_deriv_long$r1[1:288],
                         ssDNA_deriv_long$r1[1:288],
                         "7.5" = ssDNA_deriv_long$r1[289:576],
                         ssDNA_deriv_long$r1[289:576],
                         ssDNA_deriv_long$r1[289:576],
                         "8.0" = ssDNA_deriv_long$r1[577:864],
                         ssDNA_deriv_long$r1[577:864],
                         ssDNA_deriv_long$r1[577:864],
                         "8.5" = ssDNA_deriv_long$r1[865:1152],
                         ssDNA_deriv_long$r1[865:1152],
                         ssDNA_deriv_long$r1[865:1152],
                         "9.0" = ssDNA_deriv_long$r1[1153:1440],
                         ssDNA_deriv_long$r1[1153:1440],
                         ssDNA_deriv_long$r1[1153:1440],
                         "9.5" = ssDNA_deriv_long$r1[1441:1728],
                         ssDNA_deriv_long$r1[1441:1728],
                         ssDNA_deriv_long$r1[1441:1728])
### background subtract
abs_data_bkgd <- lapply(abs_data, function(x) x - ssDNA_abs)
deriv_data_bkgd <- lapply(deriv_data, function(x) x - ssDNA_deriv)
ssDNA_abs <- data.frame("Temperature" = 0,
                         "7.0" = ssDNA_abs_long$r1[1:300],
                         "7.5" = ssDNA_abs_long$r1[301:600],
                         "8.0" = ssDNA_abs_long$r1[601:900],
                         "8.5" = ssDNA_abs_long$r1[901:1200],
                         "9.0" = ssDNA_abs_long$r1[1201:1500],
                         "9.5" = ssDNA_abs_long$r1[1501:1800])
ssDNA_deriv <- data.frame("Temperature" = 0,
                           "7.0" = ssDNA_deriv_long$r1[1:288],
                           "7.5" = ssDNA_deriv_long$r1[289:576],
                           "8.0" = ssDNA_deriv_long$r1[577:864],
                           "8.5" = ssDNA_deriv_long$r1[865:1152],
                           "9.0" = ssDNA_deriv_long$r1[1153:1440],
                           "9.5" = ssDNA_deriv_long$r1[1441:1728])
### background subtract
abs_avg_bkgd <- lapply(abs_avg, function(x) x - ssDNA_abs)
deriv_avg_bkgd <- lapply(deriv_avg, function(x) x - ssDNA_deriv)
for (i in 1:length(abs_data_bkgd)){
  filename <- paste("processed_data/",
                    names(abs_data_bkgd)[[i]],
                    ".csv", sep = "")
  write_csv(abs_data_bkgd[[i]], filename)
}
```

```
abs_long_temps <- rbind(abs_temps, abs_temps, abs_temps, abs_temps, abs_temps,
abs_temps)
deriv_long_temps <- rbind(deriv_temps, deriv_temps, deriv_temps, deriv_temps, d
eriv_temps, deriv_temps)
abs_long_ph <- rep(c(7.0, 7.5, 8.0, 8.5, 9.0, 9.5), each = 300)
deriv_long_ph <- rep(c(7.0, 7.5, 8.0, 8.5, 9.0, 9.5), each = 288)
abs_bkgd_long <- lapply(abs_avg_bkgd, function(x) x <- data.frame("Temperature"
 = abs_long_temps,
                                                                  "pH" = abs_lo
ng_ph,
                                                                  "A260" = c(x$
`7.0`,x$`7.5`,x$`8.0`,x$`8.5`,x$`9.0`,x$`9.5`)))
deriv_bkgd_long <- lapply(deriv_avg_bkgd, function(x) x <- data.frame("Temperat
ure" = deriv_long_temps,
                                                                  "pH" = deriv_
long_ph,
                                                                  "A260" = c(x$
`7.0`,x$`7.5`,x$`8.0`,x$`8.5`,x$`9.0`,x$`9.5`)))
### use absolute data as input
abs_plotter <- function(input_list){
  for (i in 1:length(input_list)){
    base_pair <- names(input_list)[[i]]
    plot_title <- paste(base_pair," absolute A260 vs temperature (ssDNA backgro
und subtracted)" , sep = "")
    p <- ggplot(input_list[[i]], aes(x = Temperature, y = A260, group = pH, col
or = factor(pH))) +
      geom_point() +
      guides(color = guide_legend(override.aes = list(size = 5))) +
      xlab("Temperature / \U00B0 C") +
      ylab("A260") +
      labs(color = "pH") +
      lims(x = c(25, 80)) +
      ggtitle(plot_title) +
      theme(legend.key.size = unit(1.5, 'cm'),
            legend.title = element_text(size=20),
            legend.text = element_text(size=15))
    save_title <- paste("plots/", base_pair, sep = "")
    save_title <- paste(save_title, " abs bkgd.pdf", sep = "")
    ggsave(save_title, p)
    print(ggplotly(p))
  }
}

abs_plotter(abs_bkgd_long)
### use derivative data as input
deriv_plotter <- function(input_list){
  for (i in 1:length(input_list)){
    base_pair <- names(input_list)[[i]]
    name <- paste(base_pair, " deriv bkgd", sep = "")
    title <- paste(base_pair, " d(A260)/dT vs temperature (background corrected
)", sep = "")
    p <- ggplot(input_list[[i]], aes(x = Temperature, y = A260, group = pH, col
or = factor(pH))) +
      geom_point() +
```

```
      guides(color = guide_legend(override.aes = list(size = 5))) +
      xlab("Temperature / \U00B0 C") +
      ylab("d(A260)/dT") +
      labs(color = "pH") +
      lims(x = c(35, 80), y = c(-0.005, 0.01)) +
      theme(legend.key.size = unit(1.5, 'cm'),
            legend.title = element_text(size=20),
            legend.text = element_text(size=15)) +
      ggtitle(title)
    save_as <- paste("plots/", base_pair, " deriv bkgd.pdf", sep = "")
    ggsave(save_as, p)
    print(ggplotly(p))
  }
}
deriv_plotter(abs_bkgd_long)
```
