# Supplementary material for: An unnatural base pair for the detection of epigenetic cytosine modifications in DNA
Source: Nat Chem. 2025 Aug 20;17(11):1732–41. doi: 10.1038/s41557-025-01925-6 (PMC12580329; doi:10.1038/s41557-025-01925-6)
Supplement: Supplementary file 2 — Reporting Summary [file 41557_2025_1925_MOESM2_ESM.pdf]

nature portfolio

Corresponding author(s): Shankar Balasubramanian

Last updated by author(s): Jul 12, 2025

Reporting Summary

Nature Portfolio wishes to improve the reproducibility of the work that we publish. This form provides structure for consistency and transparency in reporting. For further information on Nature Portfolio policies, see our Editorial Policies and the Editorial Policy Checklist.

Statistics

For all statistical analyses, confirm that the following items are present in the figure legend, table legend, main text, or Methods section.

n/a	Confirmed	
☐	☒	The exact sample size (*n*) for each experimental group/condition, given as a discrete number and unit of measurement
☐	☒	A statement on whether measurements were taken from distinct samples or whether the same sample was measured repeatedly
☒	☐	The statistical test(s) used AND whether they are one- or two-sided<br>*Only common tests should be described solely by name; describe more complex techniques in the Methods section.*
☒	☐	A description of all covariates tested
☒	☐	A description of any assumptions or corrections, such as tests of normality and adjustment for multiple comparisons
☐	☒	A full description of the statistical parameters including central tendency (e.g. means) or other basic estimates (e.g. regression coefficient) AND variation (e.g. standard deviation) or associated estimates of uncertainty (e.g. confidence intervals)
☒	☐	For null hypothesis testing, the test statistic (e.g. *F*, *t*, *r*) with confidence intervals, effect sizes, degrees of freedom and *P* value noted<br>*Give P values as exact values whenever suitable.*
☒	☐	For Bayesian analysis, information on the choice of priors and Markov chain Monte Carlo settings
☒	☐	For hierarchical and complex designs, identification of the appropriate level for tests and full reporting of outcomes
☒	☐	Estimates of effect sizes (e.g. Cohen's *d*, Pearson's *r*), indicating how they were calculated

Our web collection on statistics for biologists contains articles on many of the points above.

Software and code

Policy information about availability of computer code

Data collection	UV-vis: Agilent Cary 3500 UV-Vis spectrophotometer with Agilent Cary UV Workstation (version 1.1.298; Agilent Technologies).<br>Polyacrylamide gel electrophoresis: run on CBS "Lite" vertical gel system (C.B.S. Scientific) with an EPS 3501 high voltage power supply (Amersham Pharmacia Biotech) ; imaged on BioRad ChemiDoc MP imager with Image Lab Touch (version 2.4.0.03; Bio-Rad Technologies).<br>HPLC-HRMS/MS: (oligonucleotides) Orbitrap Exploris 120 (Thermo Scientific) coupled to a Vanquish HPLC system (Thermo Scientific) using Xcalibur (Thermo Scientific).<br>NMR: 400 MHz Bruker Avance III HD Smart Probe Spectrometer, 500 MHz Bruker Avance III Smart Probe Spectrometer and 700 MHz TXO Cryoprobe Spectrometer.<br>HRMS (small molecules): Waters LCT Premier mass spectrometer (ESI-TOF).

Data analysis	DNA duplex melting analysis: Raw data cleaning, background subtraction, replicate averaging and melting curve calculation in R (RStudio 2024.04.2+764). The R script is included in the Supplementary Information for this article. Melting temperature calculation in Graphpad Prism (version 10.3.1+464).<br>Enzyme kinetics analysis: processing of polyacrylamide gel images in ImageJ (version 1.53t). Calculation of apparent rate constants in Microsoft Excel (version 16.90). Calculation of Michaelis-Menten constants (KM) and turnover numbers (kcat) by non-linear regression (fitting to Michaelis-Menten equation) in Graphpad Prism (version 10.3.1+464).<br>HPLC-HRMS/MS quantification (oligonucleotides): identification and relative quantification of product oligonucleotides in Thermo Scientific BioPharma Finder (version 4.1). Further data processing in Microsoft Excel (version 16.90).<br>Final plotting of all numerical data: Graphpad Prism (version 10.3.1+464).<br>NMR spectra: MestreNova (version 14.2.0-26256).

For manuscripts utilizing custom algorithms or software that are central to the research but not yet described in published literature, software must be made available to editors and reviewers. We strongly encourage code deposition in a community repository (e.g. GitHub). See the Nature Portfolio guidelines for submitting code & software for further information.

Data

Policy information about availability of data

All manuscripts must include a data availability statement. This statement should provide the following information, where applicable:

- Accession codes, unique identifiers, or web links for publicly available datasets
- A description of any restrictions on data availability
- For clinical datasets or third party data, please ensure that the statement adheres to our policy

Source data are provided with this paper. Raw data for the oligonucleotide mass spectrometry analysis (Fig. 5 and Extended Data Fig. 3) have been deposited in Apollo – the University of Cambridge Repository (https://doi.org/10.17863/CAM.119812).

Human research participants

Policy information about studies involving human research participants and Sex and Gender in Research.

Reporting on sex and gender	n/a
Population characteristics	n/a
Recruitment	n/a
Ethics oversight	n/a

Note that full information on the approval of the study protocol must also be provided in the manuscript.

Field-specific reporting

Please select the one below that is the best fit for your research. If you are not sure, read the appropriate sections before making your selection.

☒ Life sciences ☐ Behavioural & social sciences ☐ Ecological, evolutionary & environmental sciences

For a reference copy of the document with all sections, see nature.com/documents/nr-reporting-summary-flat.pdf

Life sciences study design

All studies must disclose on these points even when the disclosure is negative.

Sample size	n/a
Data exclusions	n/a
Replication	n/a
Randomization	n/a
Blinding	n/a

Reporting for specific materials, systems and methods

We require information from authors about some types of materials, experimental systems and methods used in many studies. Here, indicate whether each material, system or method listed is relevant to your study. If you are not sure if a list item applies to your research, read the appropriate section before selecting a response.

Materials & experimental systems

n/a	Involved in the study
☒	☐ Antibodies
☒	☐ Eukaryotic cell lines
☒	☐ Palaeontology and archaeology
☒	☐ Animals and other organisms
☒	☐ Clinical data
☒	☐ Dual use research of concern

Methods

n/a	Involved in the study
☒	☐ ChIP-seq
☒	☐ Flow cytometry
☒	☐ MRI-based neuroimaging
