# Supplementary material for: An unnatural base pair for the detection of epigenetic cytosine modifications in DNA
Source: Nat Chem. 2025 Aug 20;17(11):1732–41. doi: 10.1038/s41557-025-01925-6 (PMC12580329; doi:10.1038/s41557-025-01925-6)
Supplement: Supplementary file 4 — Unprocessed gels (Fig. 4b–e) and readme file providing context regarding no visible edges on gels in Fig. 4b–e. [file 41557_2025_1925_MOESM4_ESM.zip › NCHEM-24102742B_SourceData_Figure4/Readme_forFigure4_SourceData_Schmidletal_NatChem2025.rtf]

This readme file was generated on 2025-07-21 by David Schmidl @ Yusuf Hamied Department of Chemistry, University of Cambridge, United Kingdom.# ADDITIONAL INFORMATION ON THE IMAGING OF GELS FOR SOURCE DATA OF FIGURE 4B–DThe source image for Figure 4B shows the full gel and the edges can be made visible by adjusting the contrast.Figure 4C–D:Source images for these gels (total lane number: 15) do not show the edges of the gels, only the inner 11 lanes, respectively. The full gels were placed on the imager, but only the gel areas visible in the Source Data files were imaged. This zoomed-in imaging mode was employed to increase visibility of the bands in the loaded lanes. The outer gel lanes, which were not imaged, did not contain any loaded samples. The experiments show single nucleotide incorporation reactions into a 25mer primer by exonuclease-deficient DNA polymerases. Any longer DNA fragments produced would be visible as bands within the large area in the upper part of the imaged gel area. Bands in the lower part of the gel, which would indicate shortened primer molecules, are not expected as all polymerases used do not possess exonuclease activity, and such bands would also be visible within the imaged area. Thus, no bands were present in the far upper and lower areas of the gels which were not imaged.
