# Supplementary figures and images for: An unnatural base pair for the detection of epigenetic cytosine modifications in DNA
Source: Nat Chem. 2025 Aug 20;17(11):1732–41. doi: 10.1038/s41557-025-01925-6 (PMC12580329; doi:10.1038/s41557-025-01925-6)

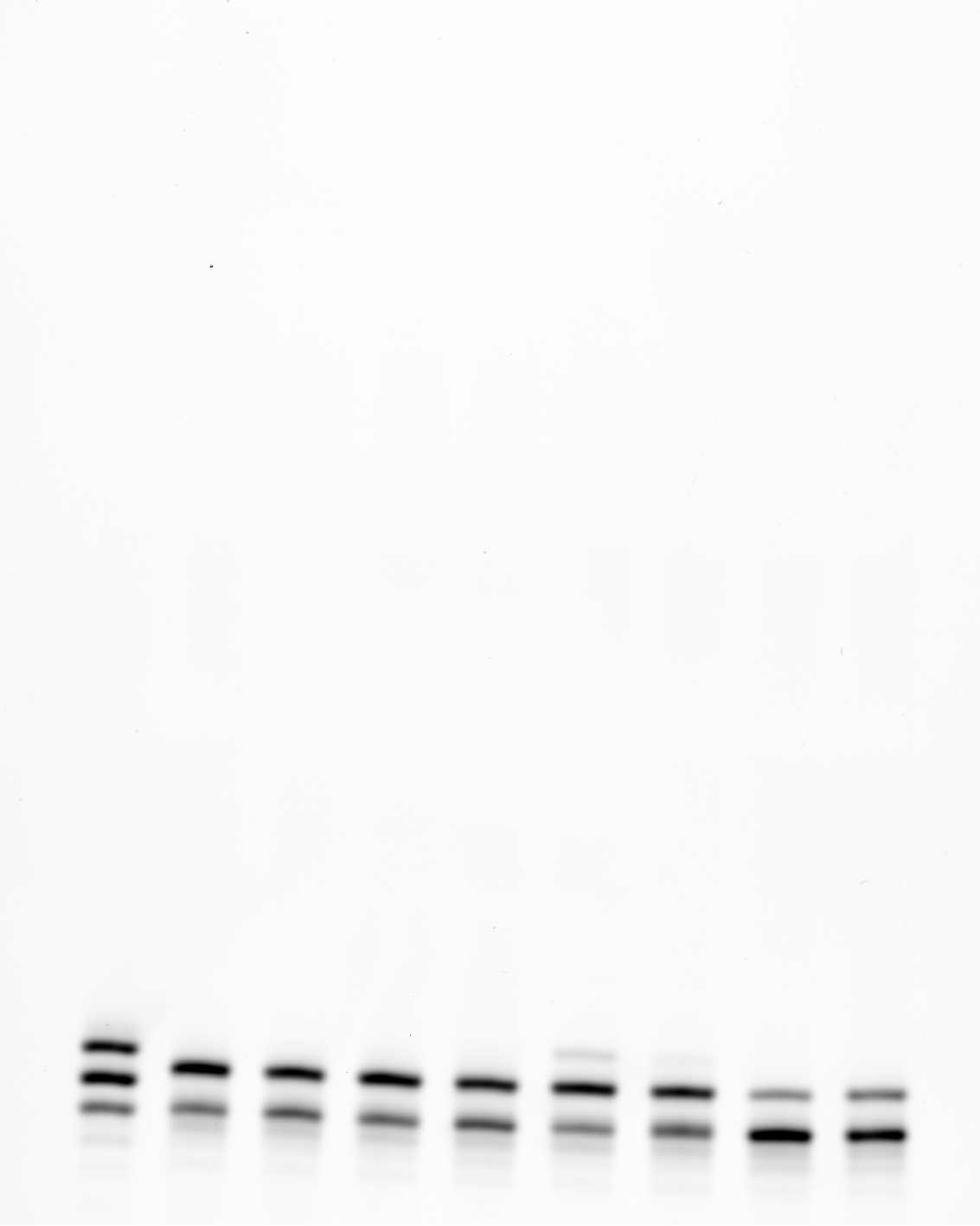

Supplement: Supplementary file 4 — Unprocessed gels (Fig. 4b–e) and readme file providing context regarding no visible edges on gels in Fig. 4b–e. [file 41557_2025_1925_MOESM4_ESM.zip › NCHEM-24102742B_SourceData_Figure4/NCHEM-24102742B_SourceData_Figure4D.tif]

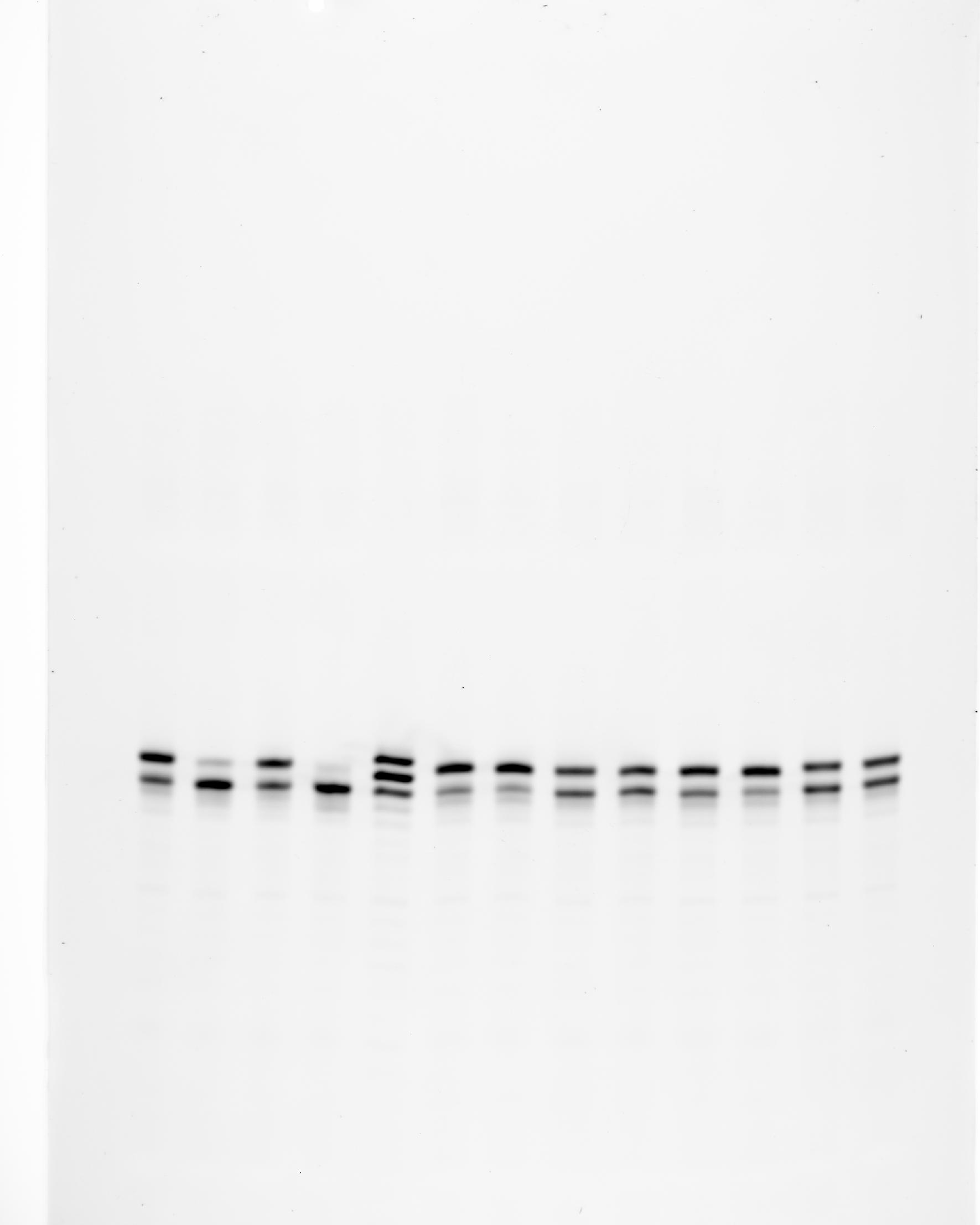

Supplement: Supplementary file 4 — Unprocessed gels (Fig. 4b–e) and readme file providing context regarding no visible edges on gels in Fig. 4b–e. [file 41557_2025_1925_MOESM4_ESM.zip › NCHEM-24102742B_SourceData_Figure4/NCHEM-24102742B_SourceData_Figure4E.tif]

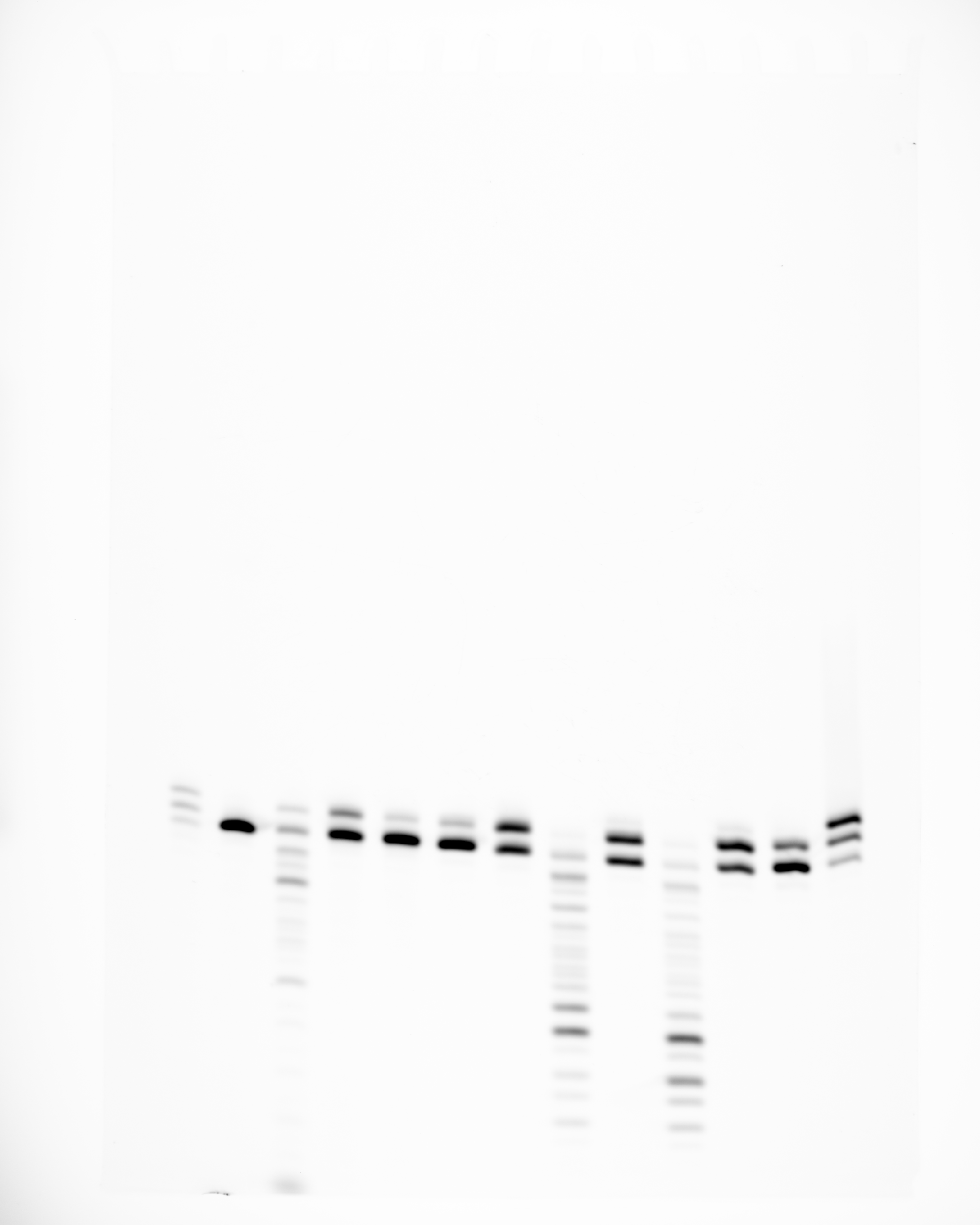

Supplement: Supplementary file 4 — Unprocessed gels (Fig. 4b–e) and readme file providing context regarding no visible edges on gels in Fig. 4b–e. [file 41557_2025_1925_MOESM4_ESM.zip › NCHEM-24102742B_SourceData_Figure4/NCHEM-24102742B_SourceData_Figure4B.tif]

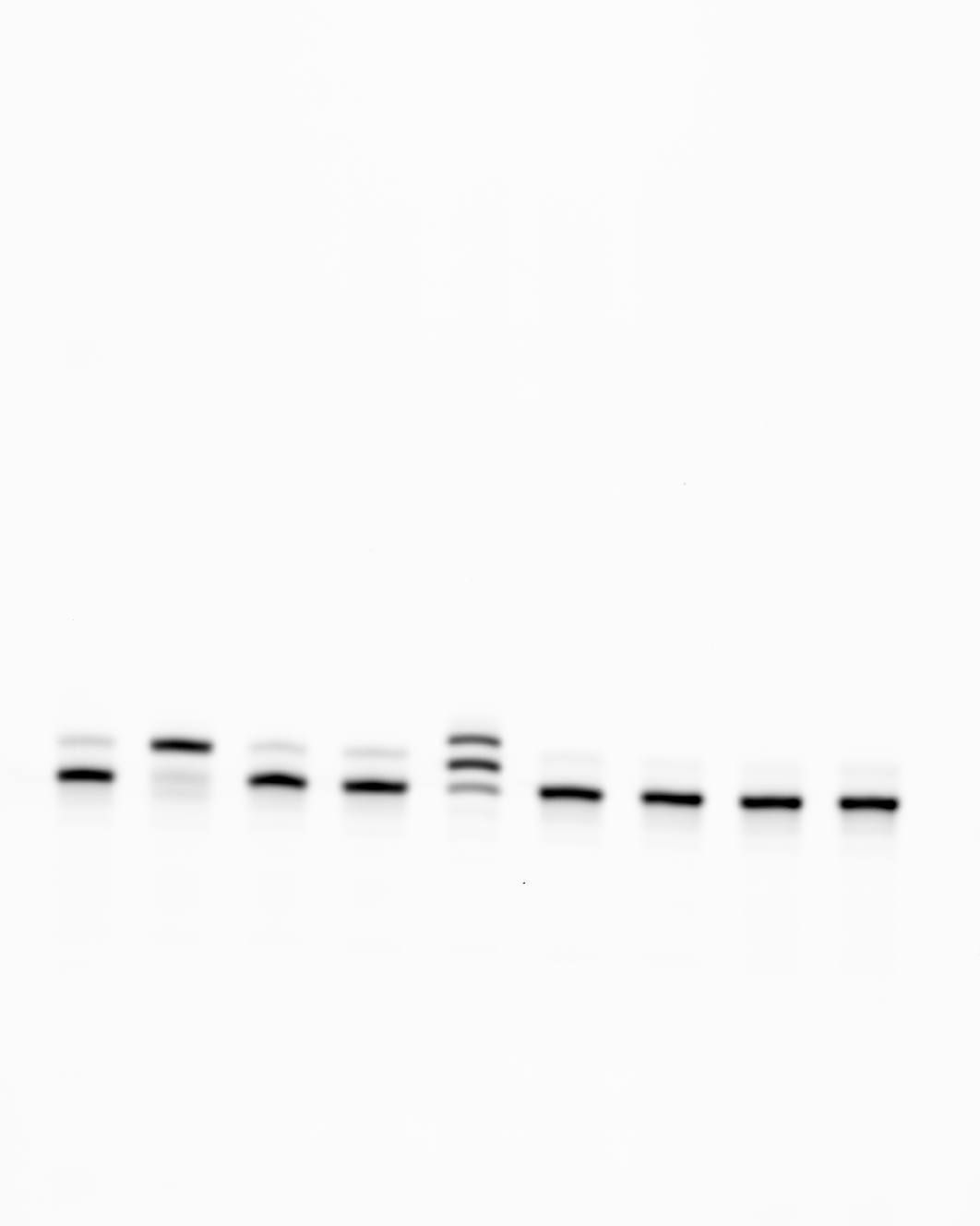

Supplement: Supplementary file 4 — Unprocessed gels (Fig. 4b–e) and readme file providing context regarding no visible edges on gels in Fig. 4b–e. [file 41557_2025_1925_MOESM4_ESM.zip › NCHEM-24102742B_SourceData_Figure4/NCHEM-24102742B_SourceData_Figure4C.tif]

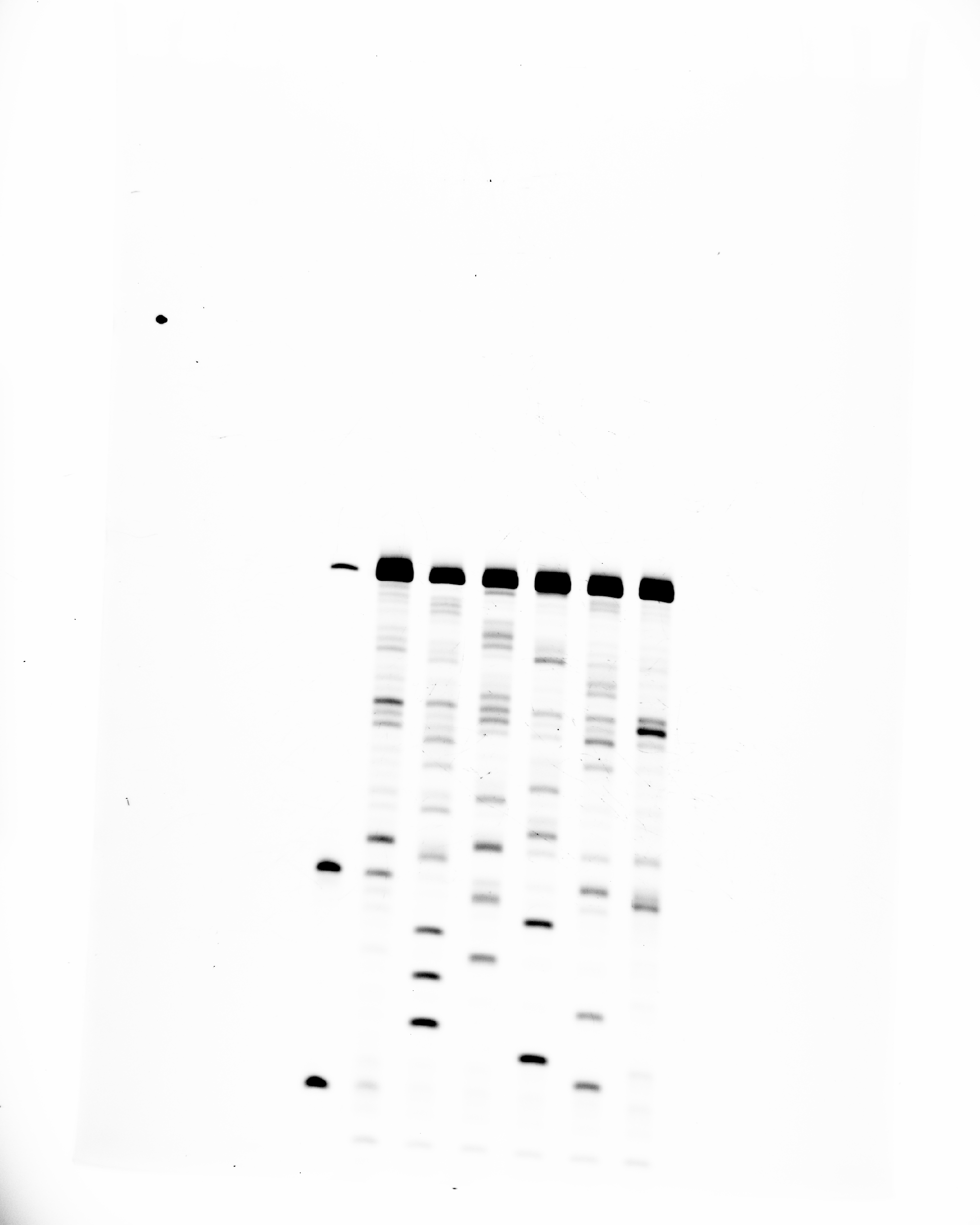

Supplement: Supplementary file 6 — Unprocessed gels (Fig. 6c,d). [file 41557_2025_1925_MOESM6_ESM.zip › NCHEM-24102742B_SourceData_Figure6/NCHEM-24102742B_SourceData_Figure6D.tif]

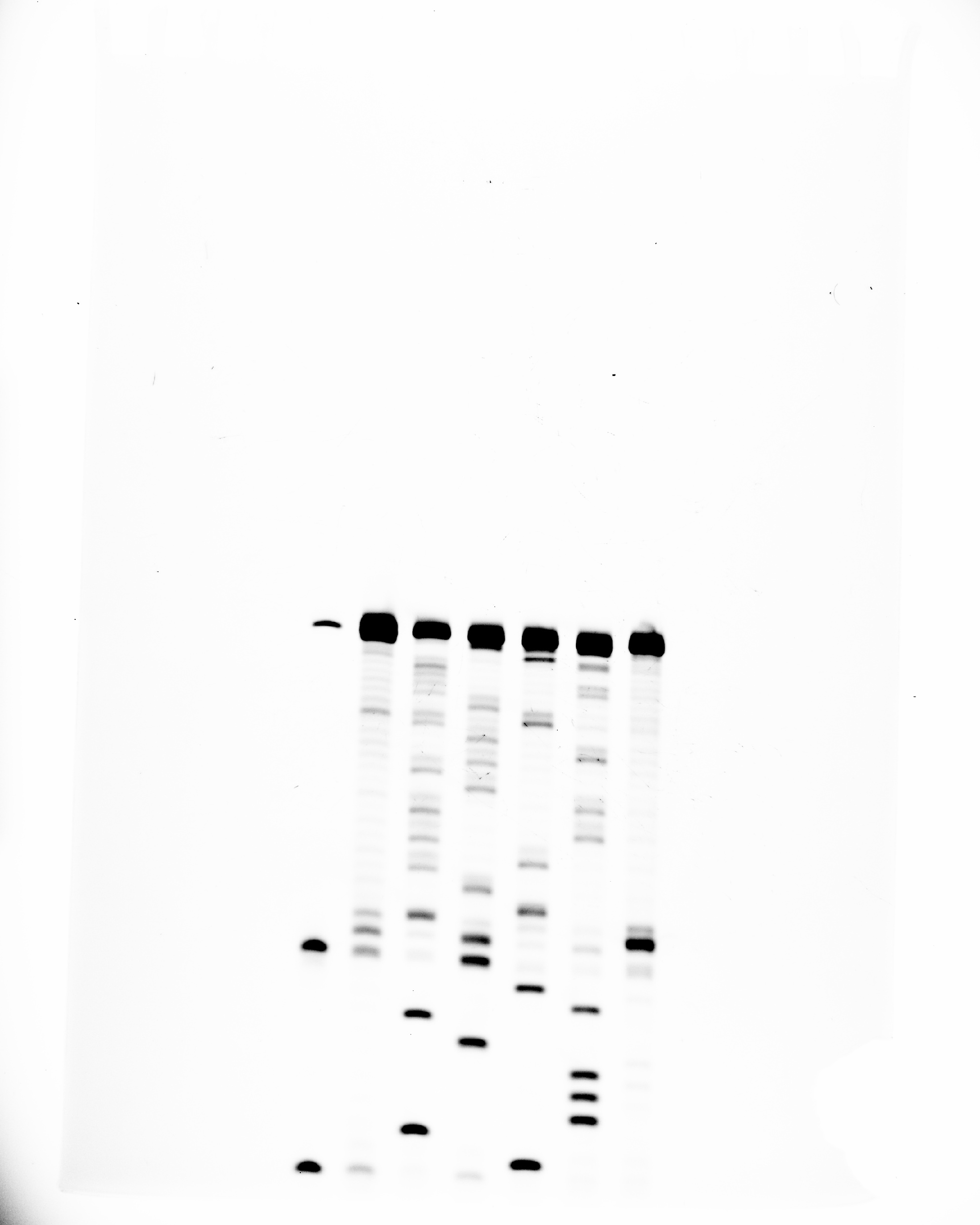

Supplement: Supplementary file 6 — Unprocessed gels (Fig. 6c,d). [file 41557_2025_1925_MOESM6_ESM.zip › NCHEM-24102742B_SourceData_Figure6/NCHEM-24102742B_SourceData_Figure6C.tif]

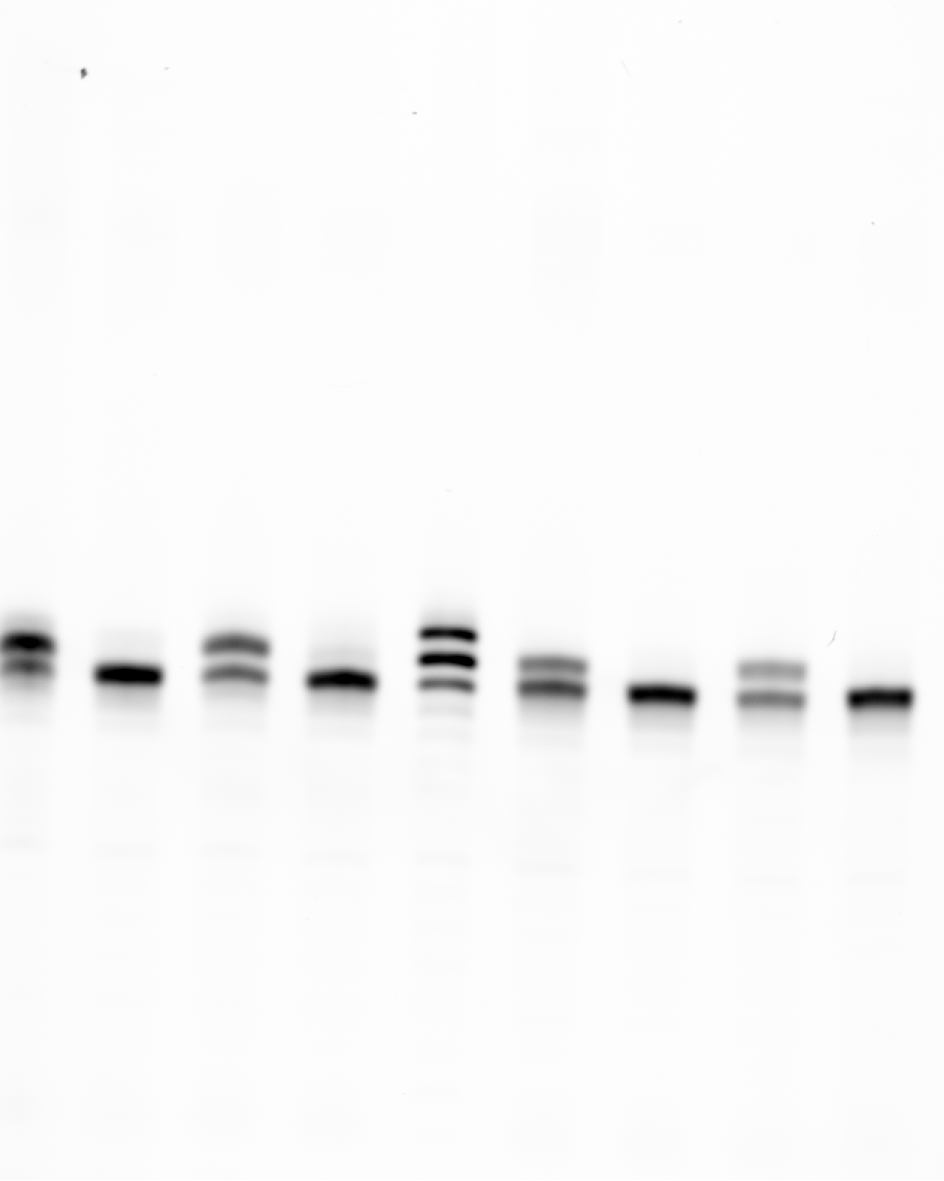

Supplement: Supplementary file 8 — Unprocessed gels for Extended Data Fig. 2a–d and f–h and readme file providing context regarding no visible edges on gels in panels a–d, g and h, and statistical source data for Extended Data Fig. 2e. [file 41557_2025_1925_MOESM8_ESM.zip › NCHEM-24102742B_SourceData_ExtendedDataFigure2/NCHEM-24102742B_SourceData_ExtendedDataFigure2H.tif]

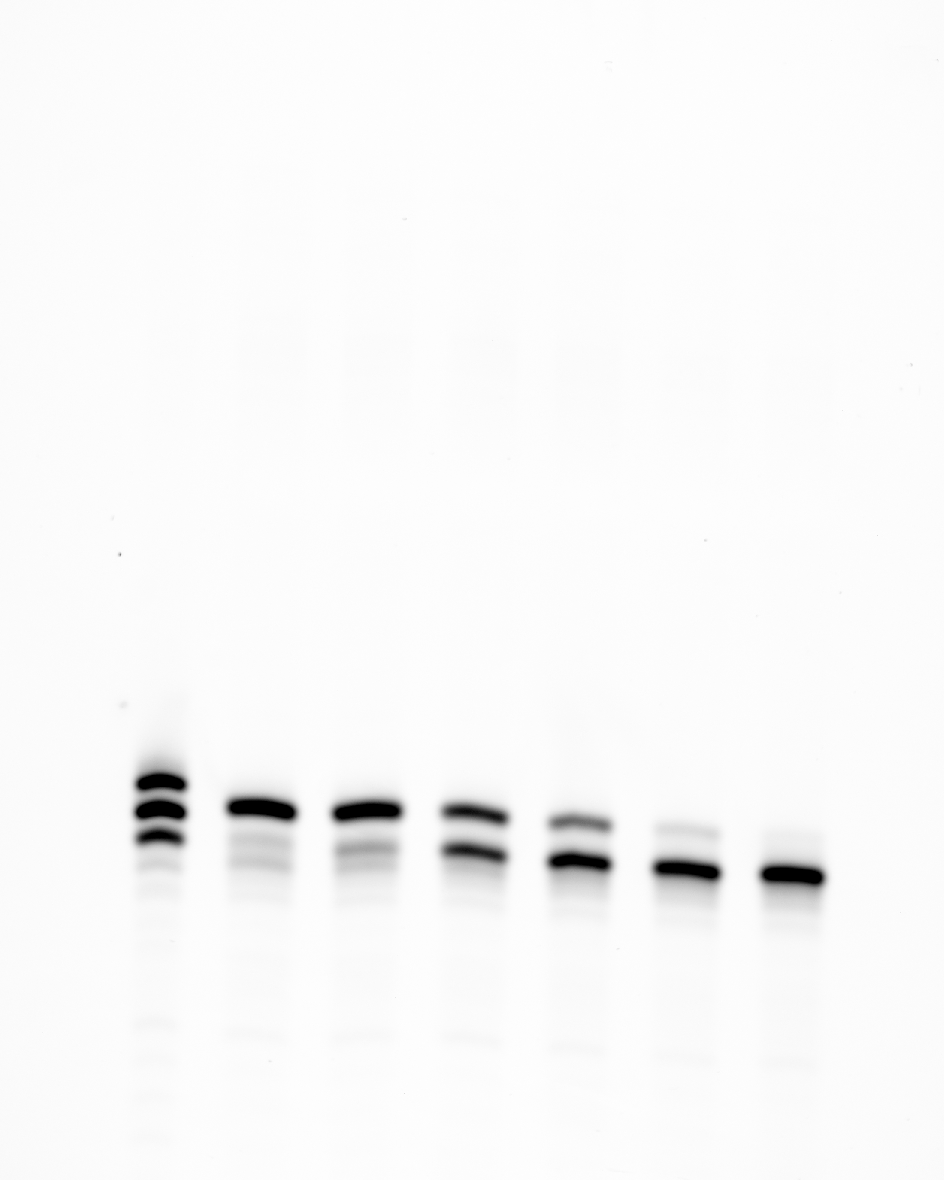

Supplement: Supplementary file 8 — Unprocessed gels for Extended Data Fig. 2a–d and f–h and readme file providing context regarding no visible edges on gels in panels a–d, g and h, and statistical source data for Extended Data Fig. 2e. [file 41557_2025_1925_MOESM8_ESM.zip › NCHEM-24102742B_SourceData_ExtendedDataFigure2/NCHEM-24102742B_SourceData_ExtendedDataFigure2G_T-template.tif]

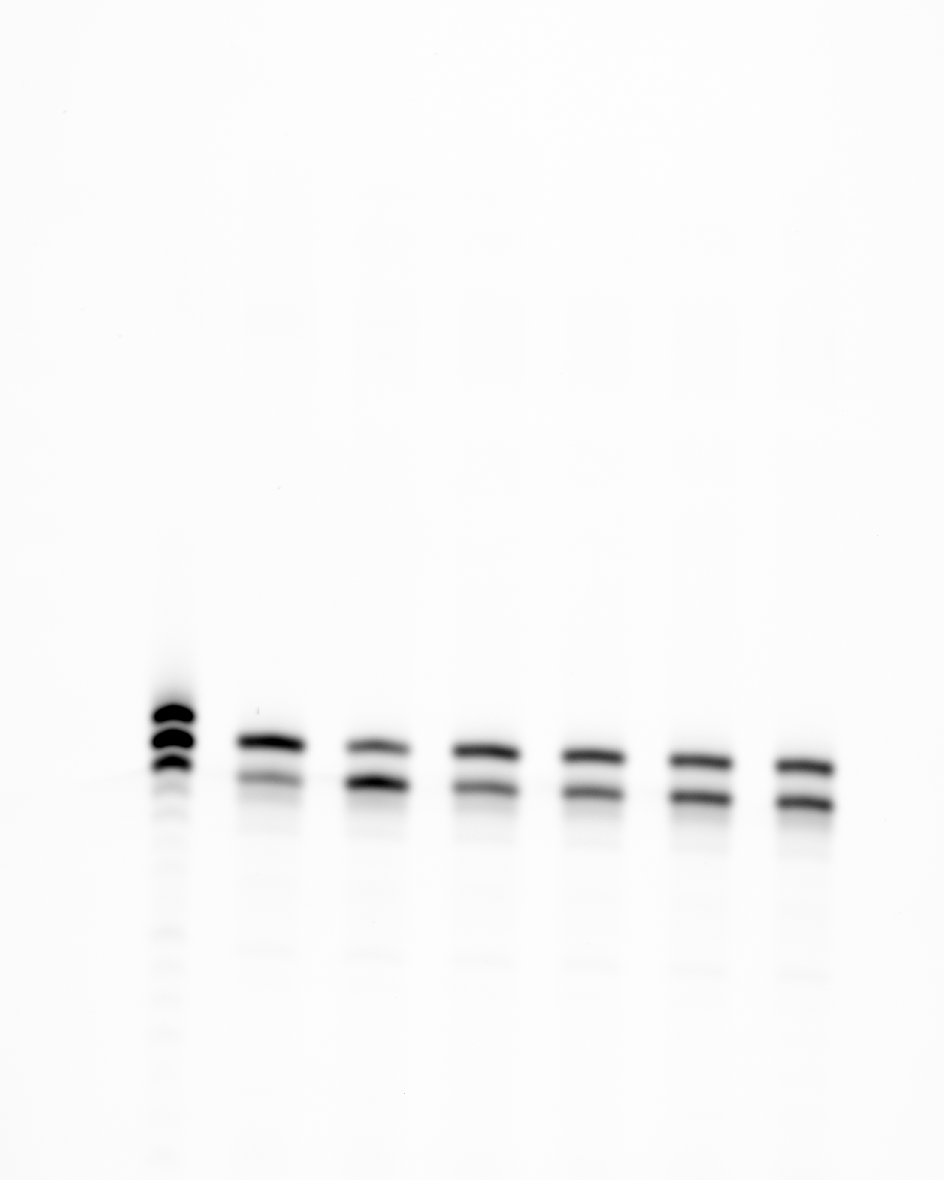

Supplement: Supplementary file 8 — Unprocessed gels for Extended Data Fig. 2a–d and f–h and readme file providing context regarding no visible edges on gels in panels a–d, g and h, and statistical source data for Extended Data Fig. 2e. [file 41557_2025_1925_MOESM8_ESM.zip › NCHEM-24102742B_SourceData_ExtendedDataFigure2/NCHEM-24102742B_SourceData_ExtendedDataFigure2G_MfC-template.tif]

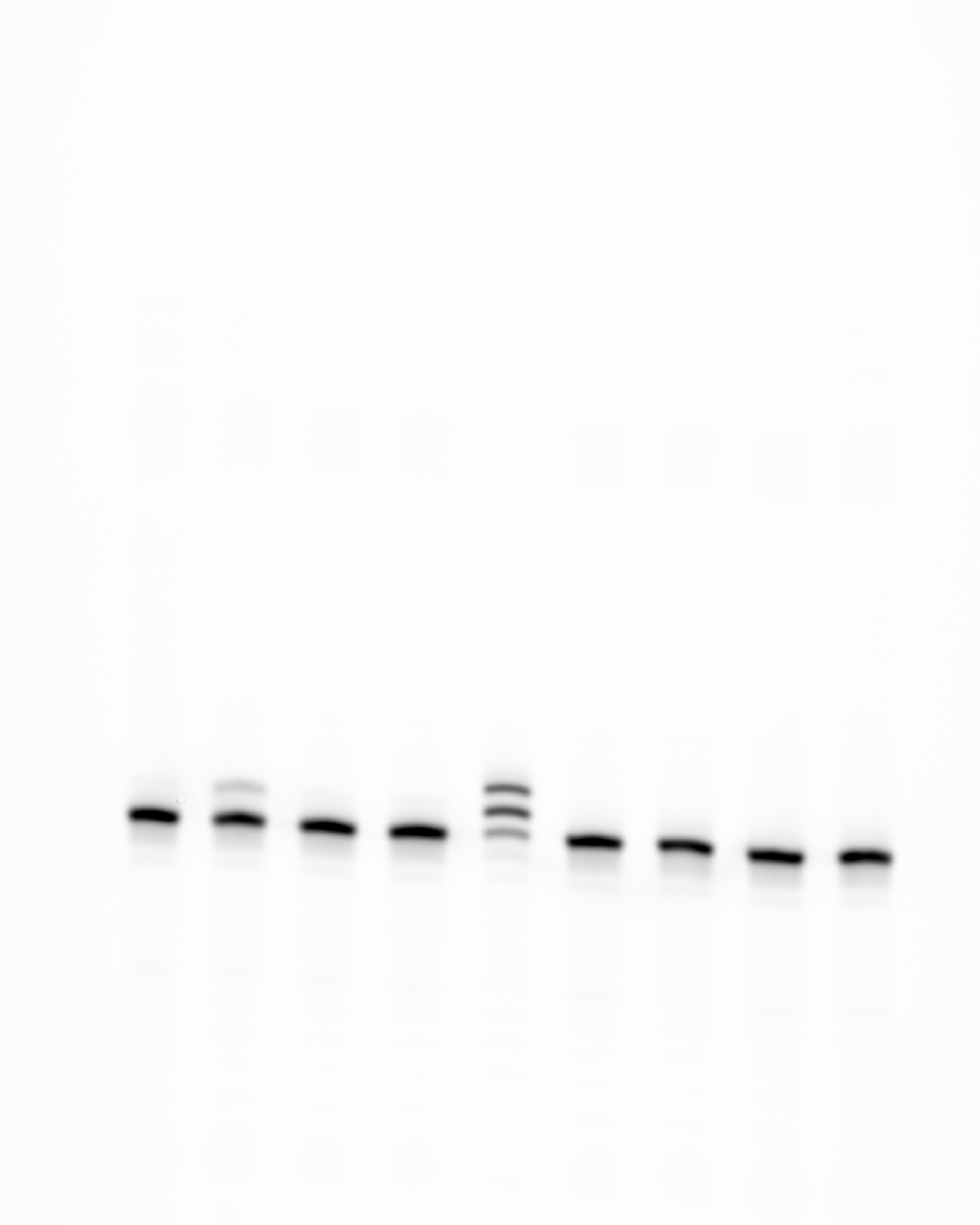

Supplement: Supplementary file 8 — Unprocessed gels for Extended Data Fig. 2a–d and f–h and readme file providing context regarding no visible edges on gels in panels a–d, g and h, and statistical source data for Extended Data Fig. 2e. [file 41557_2025_1925_MOESM8_ESM.zip › NCHEM-24102742B_SourceData_ExtendedDataFigure2/NCHEM-24102742B_SourceData_ExtendedDataFigure2C.tif]

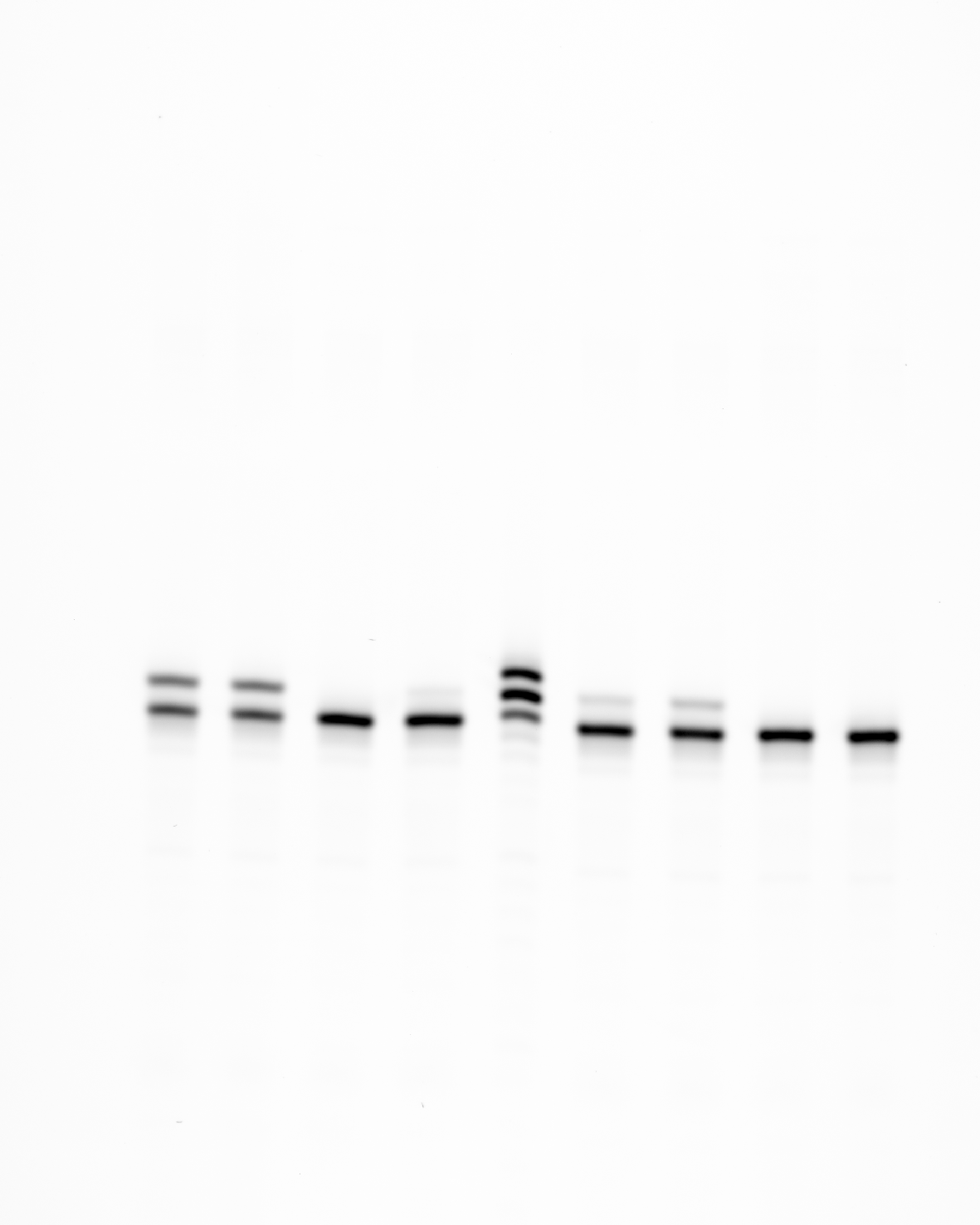

Supplement: Supplementary file 8 — Unprocessed gels for Extended Data Fig. 2a–d and f–h and readme file providing context regarding no visible edges on gels in panels a–d, g and h, and statistical source data for Extended Data Fig. 2e. [file 41557_2025_1925_MOESM8_ESM.zip › NCHEM-24102742B_SourceData_ExtendedDataFigure2/NCHEM-24102742B_SourceData_ExtendedDataFigure2B.tif]

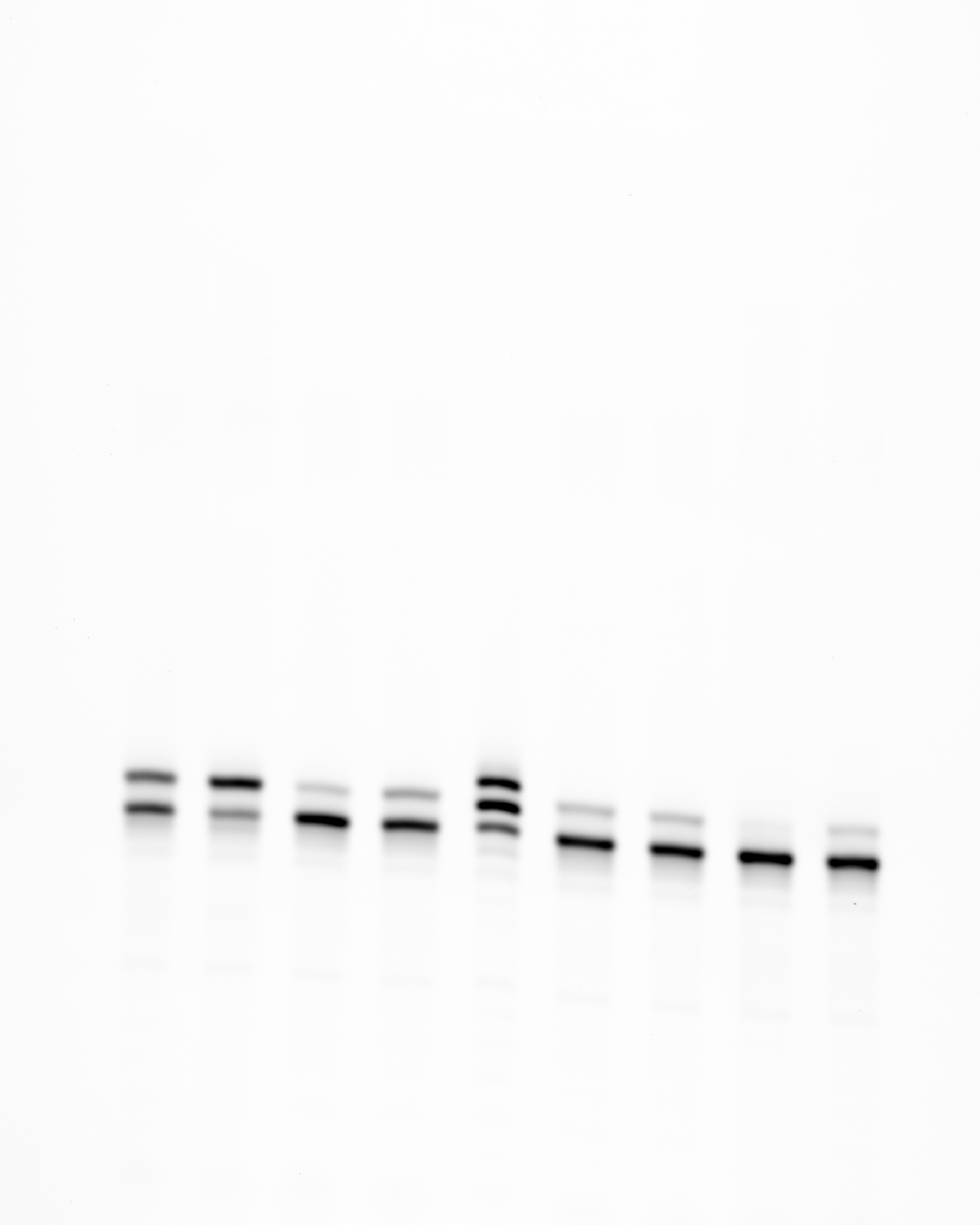

Supplement: Supplementary file 8 — Unprocessed gels for Extended Data Fig. 2a–d and f–h and readme file providing context regarding no visible edges on gels in panels a–d, g and h, and statistical source data for Extended Data Fig. 2e. [file 41557_2025_1925_MOESM8_ESM.zip › NCHEM-24102742B_SourceData_ExtendedDataFigure2/NCHEM-24102742B_SourceData_ExtendedDataFigure2A.tif]

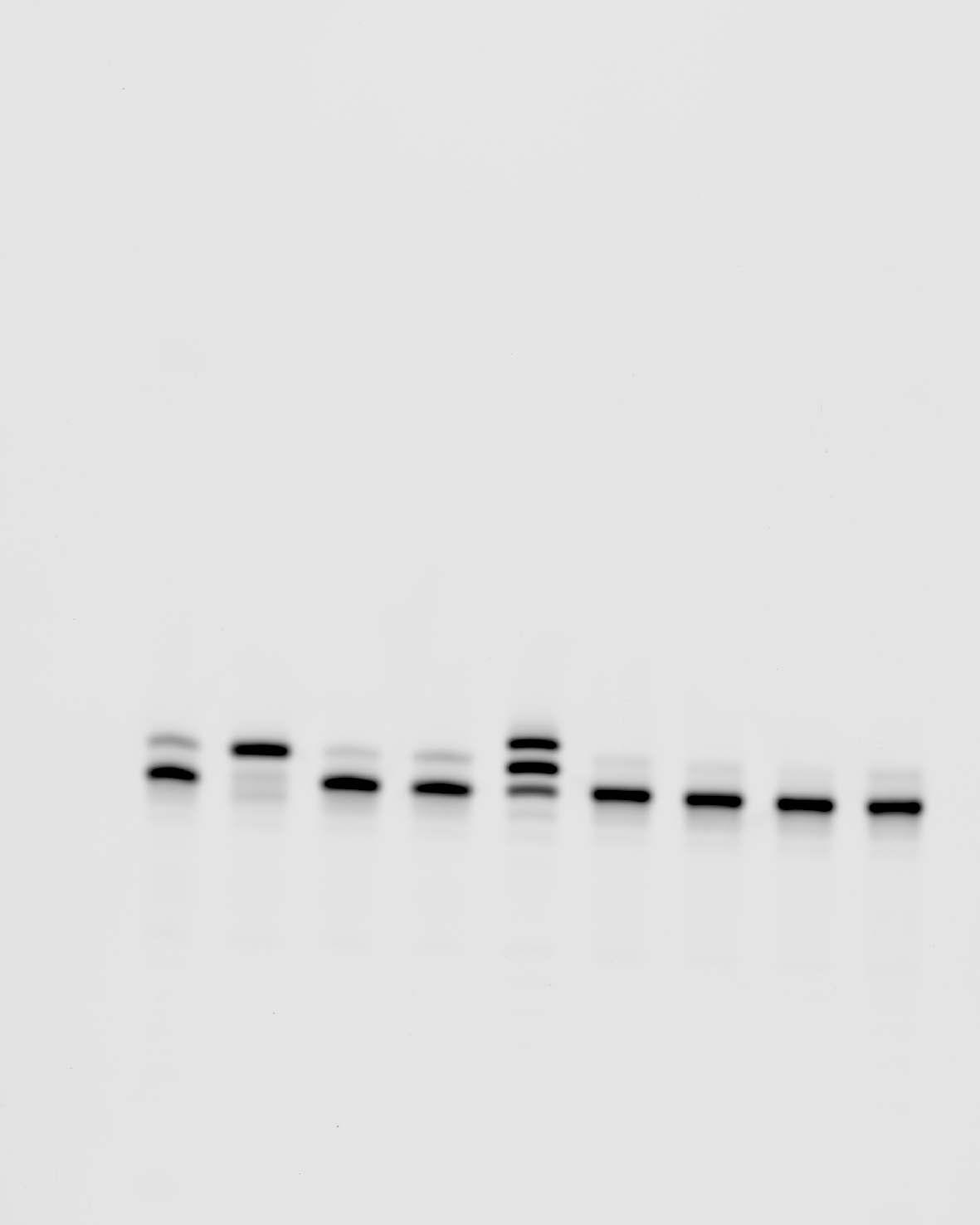

Supplement: Supplementary file 8 — Unprocessed gels for Extended Data Fig. 2a–d and f–h and readme file providing context regarding no visible edges on gels in panels a–d, g and h, and statistical source data for Extended Data Fig. 2e. [file 41557_2025_1925_MOESM8_ESM.zip › NCHEM-24102742B_SourceData_ExtendedDataFigure2/NCHEM-24102742B_SourceData_ExtendedDataFigure2D.tif]

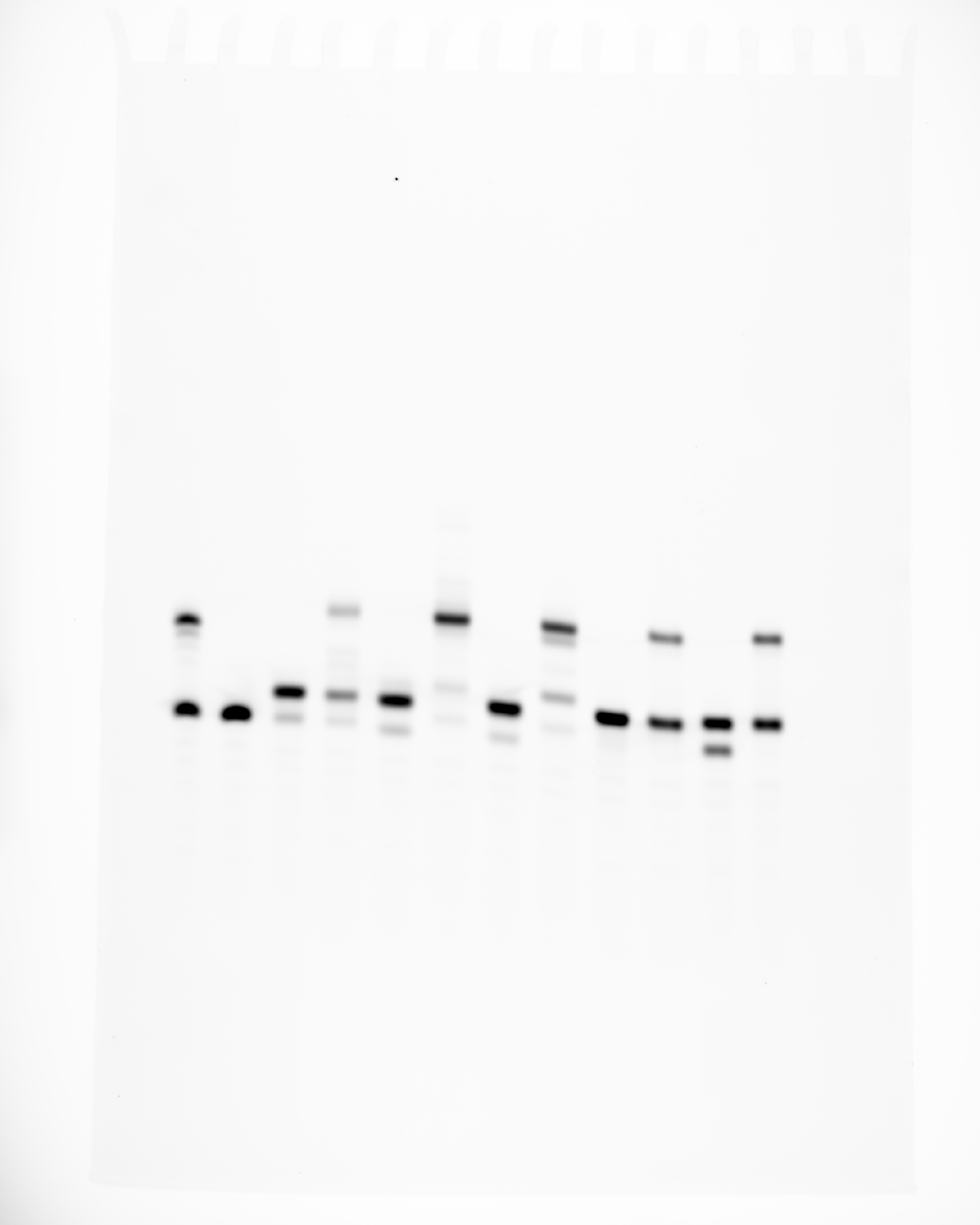

Supplement: Supplementary file 8 — Unprocessed gels for Extended Data Fig. 2a–d and f–h and readme file providing context regarding no visible edges on gels in panels a–d, g and h, and statistical source data for Extended Data Fig. 2e. [file 41557_2025_1925_MOESM8_ESM.zip › NCHEM-24102742B_SourceData_ExtendedDataFigure2/NCHEM-24102742B_SourceData_ExtendedDataFigure2F.tif]

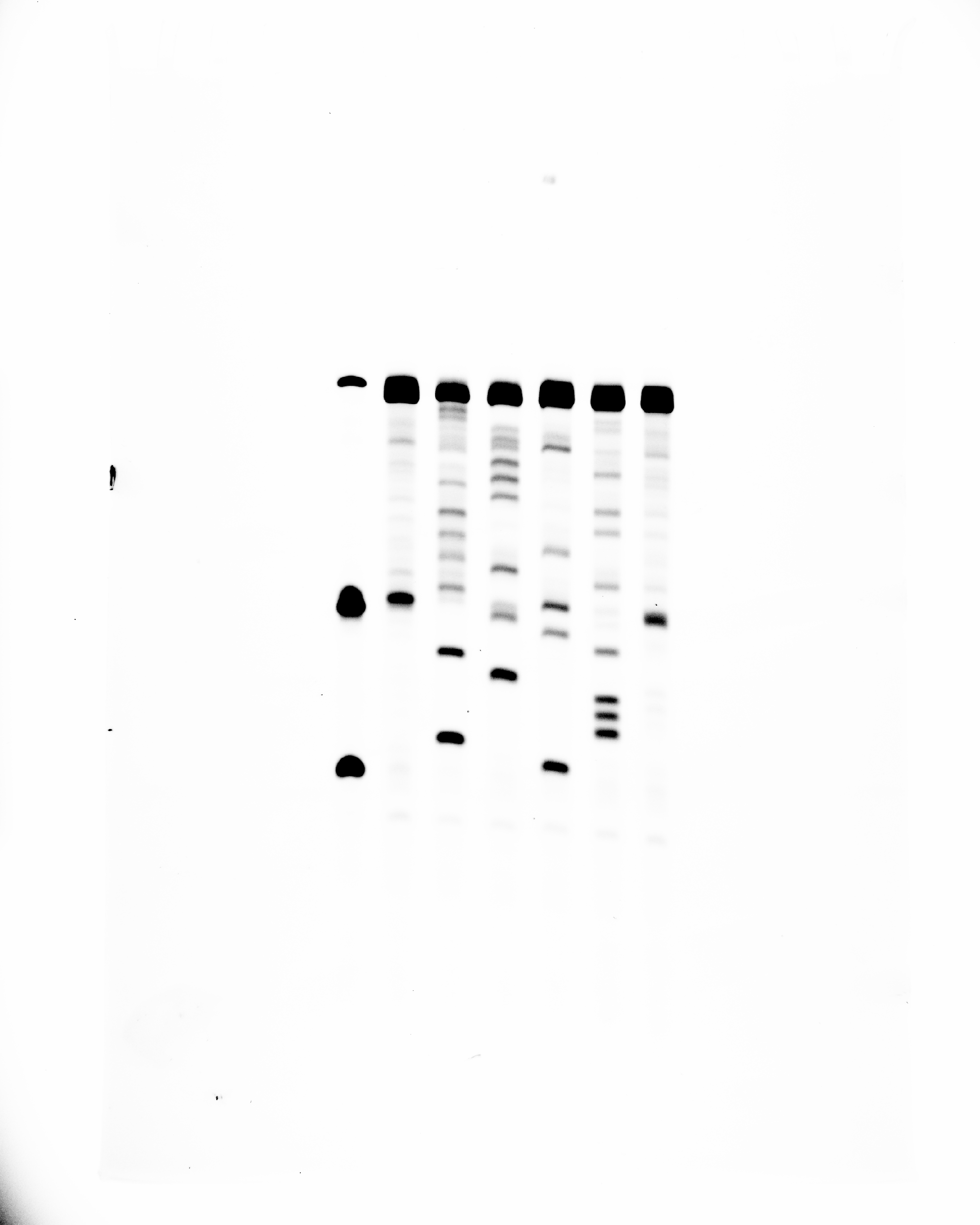

Supplement: Supplementary file 10 — Unprocessed gels. [file 41557_2025_1925_MOESM10_ESM.zip › NCHEM-24102742B_SourceData_ExtendedDataFigure4/NCHEM-24102742B_SourceData_ExtendedDataFigure4A.tif]

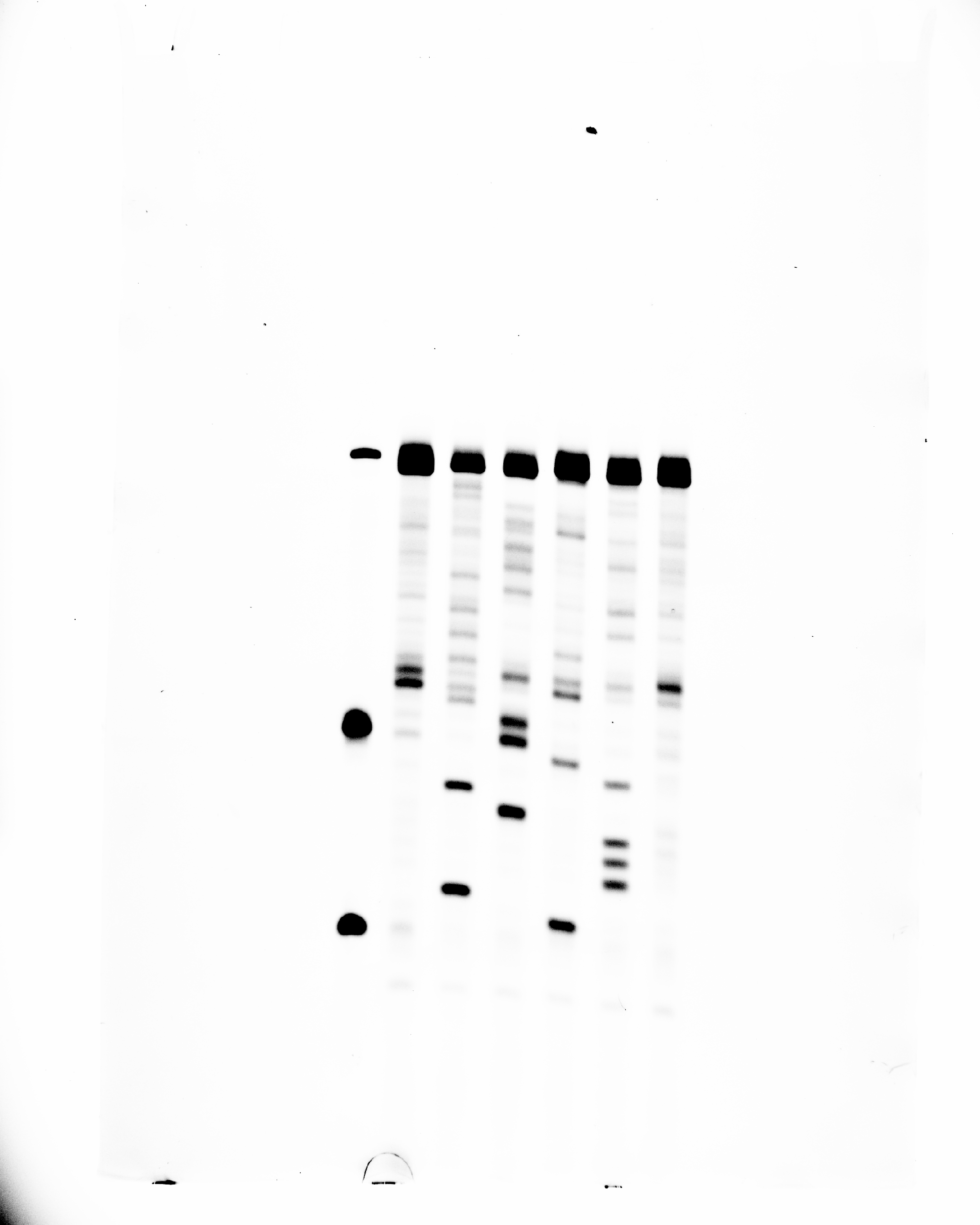

Supplement: Supplementary file 10 — Unprocessed gels. [file 41557_2025_1925_MOESM10_ESM.zip › NCHEM-24102742B_SourceData_ExtendedDataFigure4/NCHEM-24102742B_SourceData_ExtendedDataFigure4B.tif]
